# Aerobic Oxidative N-Heterocyclic Carbene-Catalyzed Formal [3+3] Cyclization for the Synthesis of Tetrasubstituted Benzene Derivatives

**DOI:** 10.1021/acs.orglett.2c03879

**Published:** 2022-12-05

**Authors:** Sara Bacaicoa, Ellymay Goossens, Henrik Sundén

**Affiliations:** Department of Chemistry and Molecular Biology, University of Gothenburg, Kemivägen 10, 412 96 Göteborg, Sweden

## Abstract

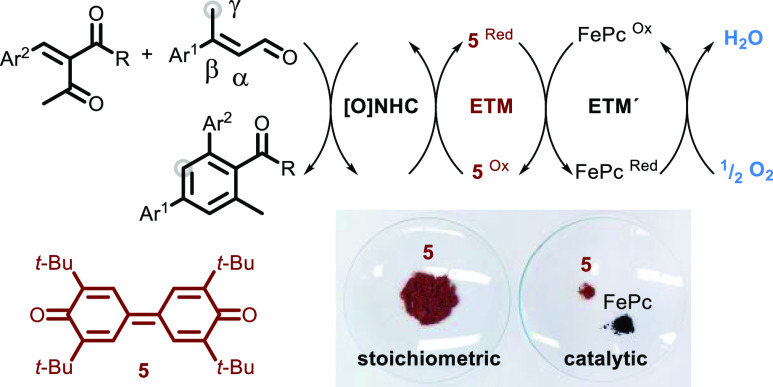

Herein, we present an accessible aerobic N-heterocyclic carbene (NHC)-catalyzed method that efficiently produces tetrasubstituted benzene rings in a single reaction. The method employs atmospheric oxygen (O_2_) as the terminal oxidant in a reaction that requires two oxidative steps. The aerobic oxidation is achieved by a selection of electron transfer mediators orchestrating a redox cascade, turning a high-energy aerobic oxidation reaction pathway into a favorable process.

Within the vibrant field of catalysis using N-heterocyclic carbenes (NHCs),^1−3^ various methodologies have been developed to oxidize the Breslow intermediate (Scheme 1A, **I**) to access the versatile *α,β*-unsaturated acyl azolium (Scheme 1A, **II**). Most commonly, these oxidations rely on the addition of a stoichiometric amount of a high-molecular weight oxidant.^4,5^ However, using a stoichiometric amount of a high-molecular weight oxidant inevitably leads to methodologies with low atom economy, low *E* factors,^6^ and potentially difficult purifications, limiting the scaling up of these protocols.

**Scheme 1:**
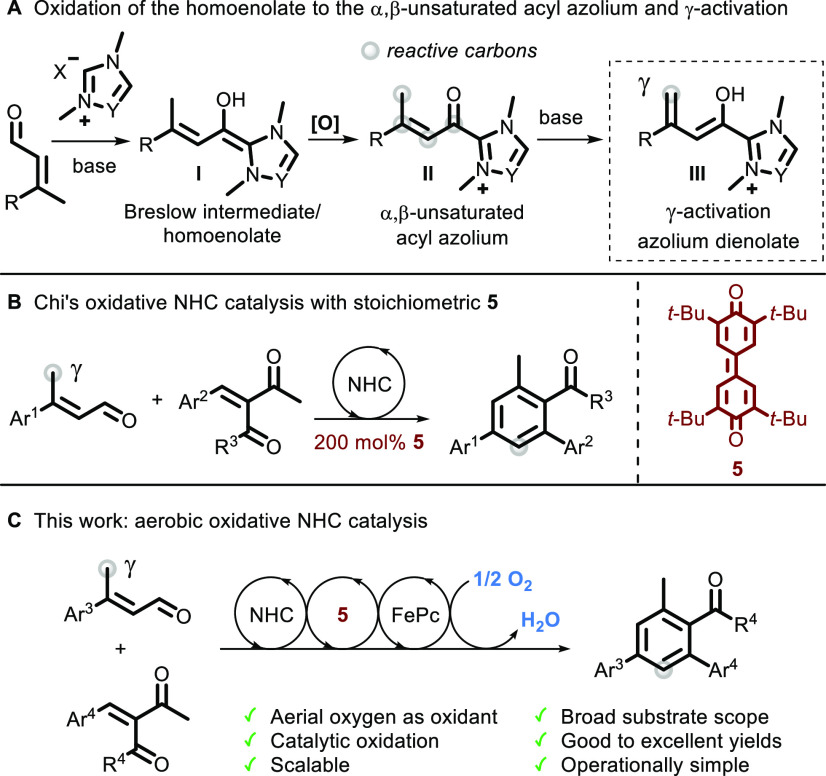
Background Information (A) Oxidation of the homoenolate to the *α,β*-unsaturated acyl azolium and γ-carbon activation. (B) Previous synthetic method using 2 equiv of the Kharasch oxidant. (C) This work using aerial oxygen as the terminal oxidant.

Recently, Chi and co-workers reported the synthesis of highly substituted benzene rings from unsaturated aldehydes and unsaturated ketones (Scheme 1B).^7−9^ The reaction proceeds via an NHC-catalyzed γ-activation of the aldehyde (Scheme 1A, **III**) and involves two oxidation events, thus requiring 2 equiv of the Kharasch oxidant (**5**) (Scheme 1B).

To circumvent the stoichiometric use of high-molecular weight oxidants, reactions that rely on O_2_ as the terminal oxidant have been developed.^10^ Atmospheric oxygen is a highly desirable oxidant as it is nontoxic and inexpensive and generates water as the sole byproduct. However, aerobic oxidative NHC catalysis that uses oxygen alone or in combination with one redox active catalyst generally suffers from poor selectivity,^11^ needs to be performed at high temperatures,^12^ or requires dry conditions with a pure oxygen atmosphere.^13,14^ A way to improve the reaction efficiency of aerobic oxidative NHC-catalyzed methods and avoid laborious reaction setups is to use a system of electron transfer mediators (ETMs).^10,15^ An ETM system consists of a selection of redox active catalysts that act in concert to transfer the electrons from the substrate to O_2_.^16,17^ By using a multistep electron transfer process, large activation energies can be avoided and turn aerobic oxidations into kinetically favored processes. To date, only aerobic NHC-catalyzed esterifications, N-acylations, and lactonizations have been described in the literature using systems of ETMs.^18−24^

Herein, we present an aerobic oxidative NHC-catalyzed γ-activation for the synthesis of arenes (Scheme 1C), which requires neither stoichiometric high-molecular weight oxidants, a pure oxygen atmosphere, nor photochemical activation in the presence of precious metal-containing photocatalysts.^13^ Our method stands out because of its simplicity and mild conditions.

Optimization studies with *α,β*-unsaturated aldehyde **1a** and diketone **2a** revealed that a [3+3] cyclization via γ-carbon activation is readily enabled in the presence of 30 mol % NHC precatalyst **4a**, cesium carbonate, and both redox active catalysts **5** and **6** (each at 10 mol %) under aerobic conditions. The optimal reaction procedure afforded the desired product **3a** in 76% yield (Table 1, entry 1; see the Supporting Information for full optimization data). The presence of ETMs **5** and **6** was key for the conversion to substituted benzene ring **3a**, as the absence of one or both species drastically impacted the reaction efficiency (Table 1, entries 2–4). To optimize the amount of ETMs in the reaction, our conditions were challenged by using both **5** and **6** (each at 5 mol %), resulting in a lower yield (Table 1, entry 5), as well as reducing the level of **6** to only 5 mol % (Table 1, entry 6). Moreover, running the reaction without ETM **5** and using 20 mol % **6** in the presence of oxygen yielded 17% of **3a**, suggesting that iron(II) phthalocyanine **6** in combination with O_2_ can also directly perform the two oxidations in the reaction, but in a less efficient manner (Table 1, entry 7). The role of other reaction parameters was also investigated. Thereby, a decrease in the temperature from the optimal conditions resulted in lower conversion (Table 1, entries 8 and 9), as well as a decrease in the amount of NHC precatalyst to 20 mol % or a decrease in the amount of cesium carbonate to 1.5 equiv (Table 1, entry 10 or 11, respectively). A decreased yield was also observed when the amount of aldehyde **1a** was decreased to 1.5 equiv (Table 1, entry 12). Likewise, having an excess of 2 equiv of diketone **2a** with respect to aldehyde **1a** was detrimental to the reaction performance (Table 1, entry 13). Finally, the solvent 2-methylTHF was tested as an important green alternative to THF, but the yield was lower than under the optimal conditions (Table 1, entry 14).

**Table 1:**
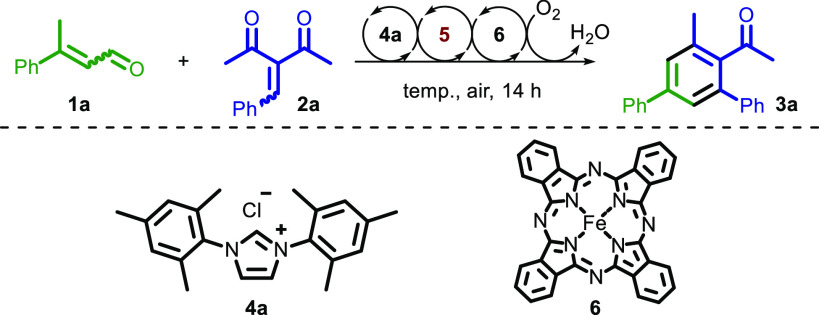
Optimization of NHC-Catalyzed Aerobic [3+3] Cyclization of *α,β*-Unsaturated Aldehydes

entry	change from the optimal conditions	yield (%)^b^
1	no change^a^	76^c^
2	without **5**	8
3	without **6**	8
4	without **5** and **6**, only atmospheric O_2_ as the oxidant	7
5	**5** (5 mol %) and **6** (5 mol %)	52^c^
6	**6** (5 mol %)	30
7	without **5** and with **6** (20 mol %)	17
8	19 °C	70^c^
9	0 °C	27
10	**4a** (20 mol %)	56
11	Cs_2_CO_3_ (1.5 equiv)	68^c^
12	**1a** (1.5 equiv)	57^c^
13	**1a** (0.2 mmol) and **2a** (0.4 mmol)	54
14	2-methylTHF as the solvent	52^c^

a Optimal reaction conditions: **2a** (0.2 mmol), **1a** (0.4 mmol, 2 equiv), **6** (0.02 mmol, 10 mol %), **5** (0.02 mmol, 10 mol %), **4a** (0.06 mmol, 30 mol %), cesium carbonate (0.4 mmol, 2 equiv), THF (4 mL), 25 °C, air, 14 h.

b GC-FID yield.

c Isolated yield.

With our optimized reaction conditions in hand, we tested the compatibility of our method with a variety of unsaturated ketones and aldehydes. Mixtures of *E*/*Z* enals were used for the substrate scope and for the optimization.

In the first part of the substrate scope, different enones and β-keto esters were reacted with enal **1a** (Scheme 2). Electron-rich enones with methyl and methoxy at the *para* position of the enone aromatic ring were well tolerated, affording the products in 54% and 50% yields, respectively (**3f** and **3l**). Halogens were also appropriate candidates for this reaction. For example, *m-*Br and *p-*Cl gave the corresponding halogenated acetophenones in 98%, and 86% yields, respectively (**3c** and **3e**). The presence of fluorine at the *ortho* position did not negatively affect the reaction, providing **3b** in 70% yield. The reaction also proceeded with an ethyl ketone moiety, giving the desired product **3d** in 46% yield. Enal **1a** could be reacted with a heteroaromatic enone containing thiophene to give **3g** in 85% yield. Extended aromatics are also compatible with the reaction finding an example in the product, for example, **3h** that was isolated in 71% yield.

**Scheme 2:**
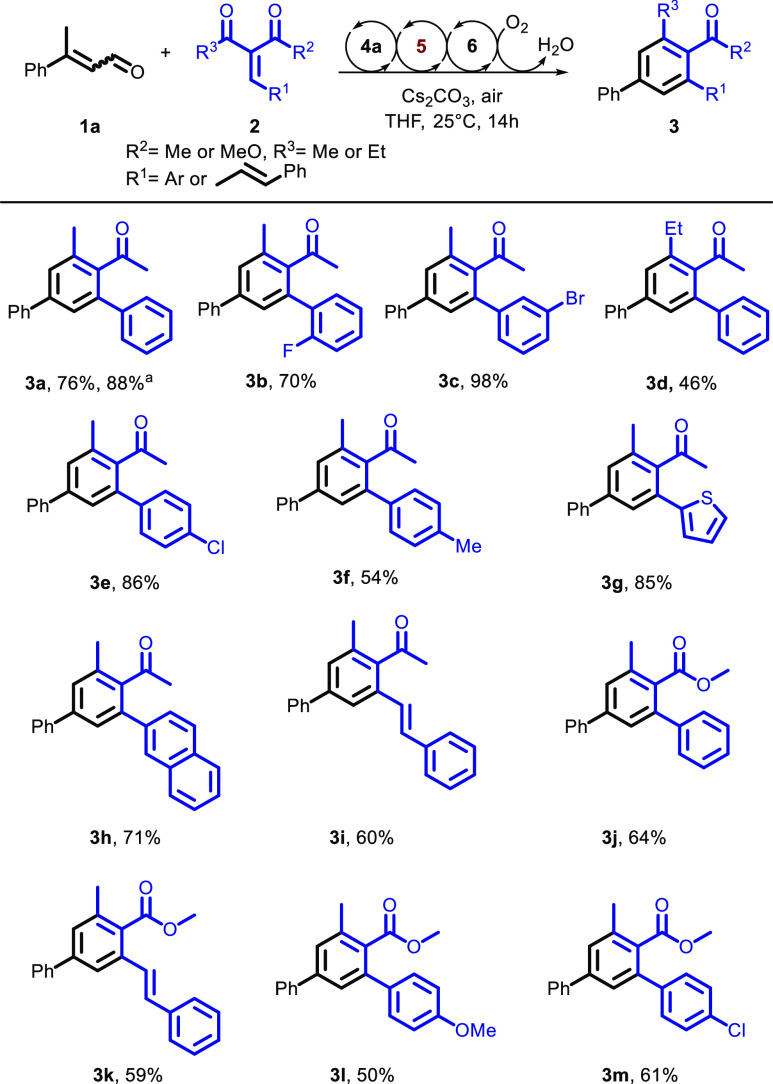
Scope of the Enone for the Aerobic Oxidative NHC Catalysis for the Synthesis of Benzene Derivatives Isolated yield of a 1 mmol scale experiment. Substrate scope determined under the optimal conditions from Table 1.

Reaction with vinyl aromatic dienone resulted in product **3i** in 60% yield. β-Keto esters were also suitable electrophiles in the reaction forming the corresponding aryl benzoates in good yields (entries **3j–3m**). Overall, the reactions carried out with dienone substrates provided yields higher than the yields of those employing β-keto esters, presumably because the latter are less electrophilic Michael acceptors. Accordingly, this explains why electron-donating groups at the *para* position of the aromatic ring on the dienone led to lower yields (**3f** and **3l**). The best result was achieved with bromo-substituted enone at the *meta* position of the aromatic ring, resulting in a yield of 98% (**3c**).

Next, we explored the compatibility of a variety of enals with our method to yield products **3n–3z** (Scheme 3). Two different dienones, namely, **2a** and **2h**, were used to show the diversity of this reaction. An electron-donating methyl group at the *meta* position on the aromatic ring of the enal was well tolerated, resulting in product **3n** in 96% yield. Similarly, methoxy groups at the *para* position of the aromatic ring of the enal afforded the products in good yields (**3o** and **3w**). Halogens incorporated at the *meta* position of the aromatic ring of the enal were also compatible, giving the products in 82% and 76% yields (**3p** and **3s**, respectively). A similar trend occurred for a chloro-substituted enal at the *para* position leading to formation of product **3q** in 75% yield. On the contrary, the presence of a chlorine atom at the *ortho* position resulted in a lower yield of 40% (**3r**), which could be explained by steric hindrance effects. When the aromatic ring on the enal was replaced by a naphthalene moiety, the reaction was positively affected, resulting in product **3t** in 82% yield. Other heteroaromatic rings such as thiophene and furan were also compatible with the reaction, resulting in yields of 89% and 83%, respectively (**3u** and **3v**). In general, the reaction afforded the highest yields when the enal substrates were reacted with electron poor Michael acceptor **2a** except for product **3r**, which was affected by steric hindrance. The enals could also react with the more conjugated vinyl dienone **2h** leading to products **3w–3z** in good yields. However, the reaction did not provide the desired product **3aa**, indicating that this strategy is not compatible with the use of non-aromatic *α,β*-unsaturated enals. In addition, an aldehyde with an extended γ position with a methyl group is not suitable for this reaction because the desired product **3ab** was not found.

**Scheme 3:**
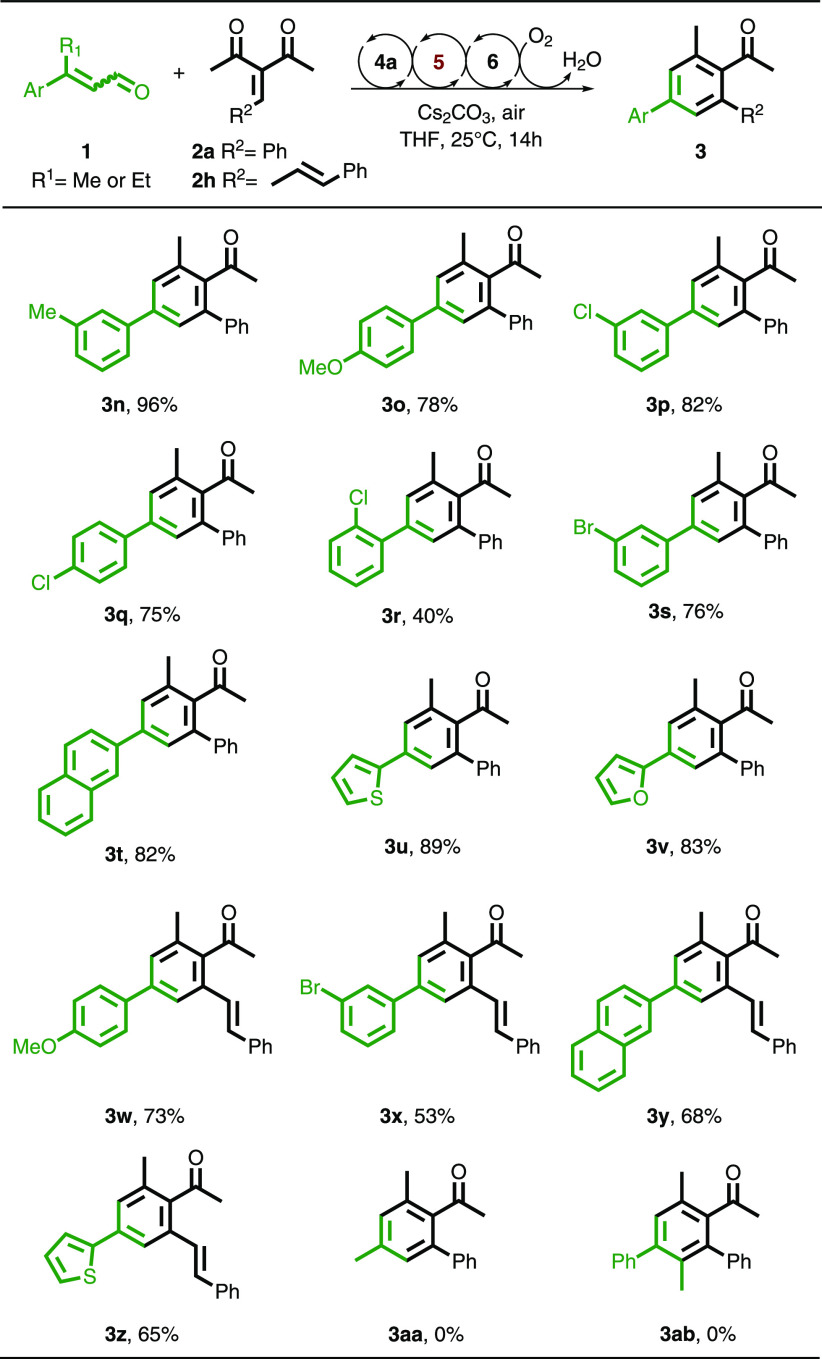
Scope of the Enal for the Aerobic Oxidative NHC Catalysis for the Synthesis of Benzene Derivatives Substrate scope run with the optimal conditions from Table 1.

To better understand the influence of the different ETMs, we monitored the progression of the reaction over time using GC-FID (Figure 1). When the reaction was performed with 2 equiv of **5** alone under nitrogen (Figure 1b), we obtained a yield comparable to that attained with our catalytic oxidation approach (Figure 1a). Mechanistically, elimination experiments showed that the reaction performed without **5** stopped after 1 h with an 8% yield (Figure 1c), and a similar trend was established upon removal of **6** (Figure 1d). In the absence of both **5** and **6** and with atmospheric oxygen as the only oxidant, the reaction afforded a yield of <7% (Figure 1e). Analysis of the reaction mixture showed that the enal is fully consumed after 5 h, indicative of competing kinetic side reactions.

**Figure 1:**
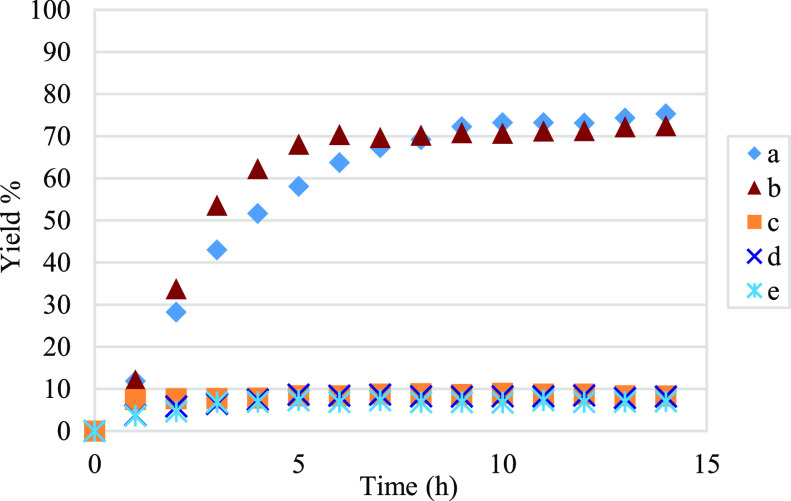
Kinetic profile of the aerobic [3+3] annulation, determined via elimination experiments. (a) Optimal conditions. (b) Two equivalents of **5** under N_2_, without **6** and without O_2_. (c) Optimal conditions without **5**. (d) Optimal conditions without **6**. (e) Optimal conditions without **5** and **6**, only atmospheric O_2_ as the oxidant.

The proposed mechanism of this reaction is initiated via the cesium carbonate-assisted deprotonation of imidazolium salt **4a** generating the active carbene catalyst (Scheme 4). After the subsequent nucleophilic attack of the carbene on *α,β*-unsaturated aldehyde **1a**, Breslow intermediate **I** is formed. The oxidation of **I** to acyl azolium **II** is carried out by oxidant **5**, which is regenerated by the ETM system with aerial oxygen as the terminal electron acceptor. After this catalytic oxidation, the reaction continues via γ-deprotonation to give **III**. Subsequent steps are a Michael addition on **2a**, followed by γ-deprotonation of **IV** and an intramolecular aldol reaction leading to cyclic intermediate **VI**. After a lactonization and decarboxylation, the last oxidative event involves the ETM system a second time, producing the final product **3a**.

**Scheme 4:**
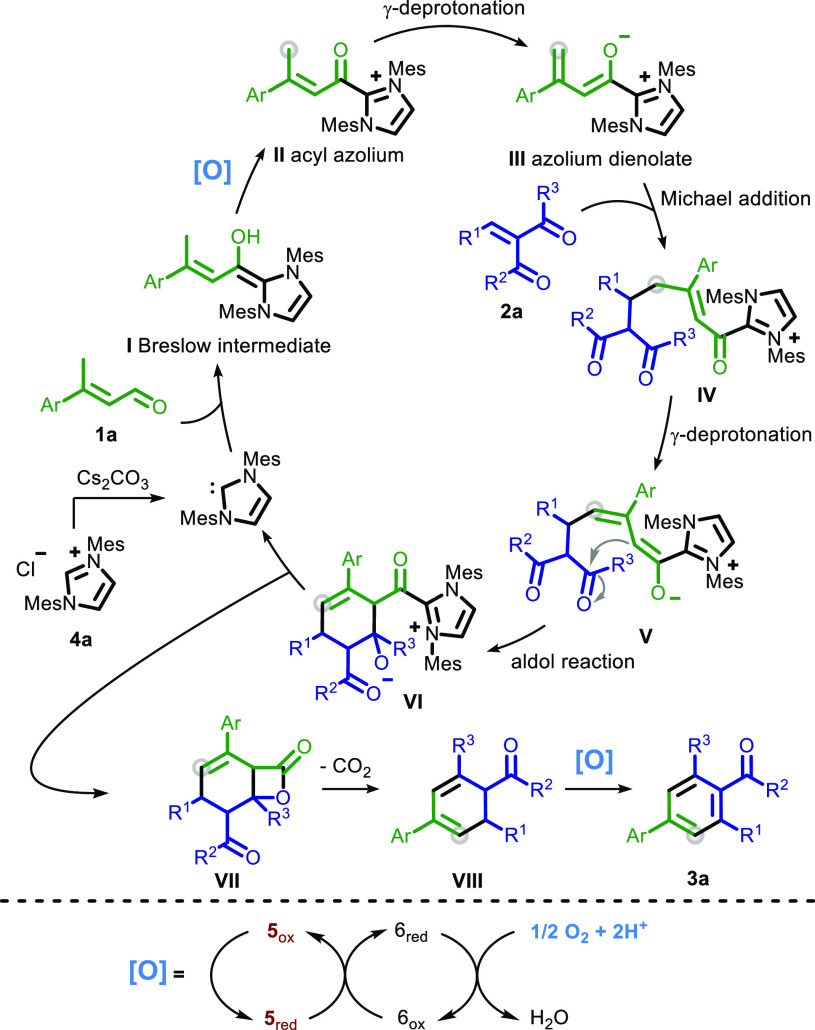
Proposed Catalytic Cycle

Importantly, this method provides very potential high value molecular platforms in applied chemistry fields such as medicinal and physical chemistry and material science. Molecules with biological activity or photochemical properties, such as indenes and fluorenones, respectively, can be synthesized after one or two extra derivatization steps.^7,25,26^ Tetrasubstituted acetophenones such as **3a** can be converted to highly functionalized oxo triphenylhexanoates (OTHOs) **7**, a class of substances that find important use as gelators in the field of material chemistry, with a yield of 38% (Scheme 5A).^27−31^ The production of OTHOs requires only one extra synthetic step in a four-component reaction in which the yield per bond-forming step is 72%. Alternatively, isocoumarins, natural products of pharmacological interest, can be accessed by derivatization of aromatic ester **3k** (Scheme 2). For example, arene **3k** can be converted into an isocoumarin by sequential hydrolysis with lithium hydroxide and later lactonization with a selenium catalyst and a hypervalent iodine oxidant providing **8** in 52% yield.^32,33^

**Scheme 5:**
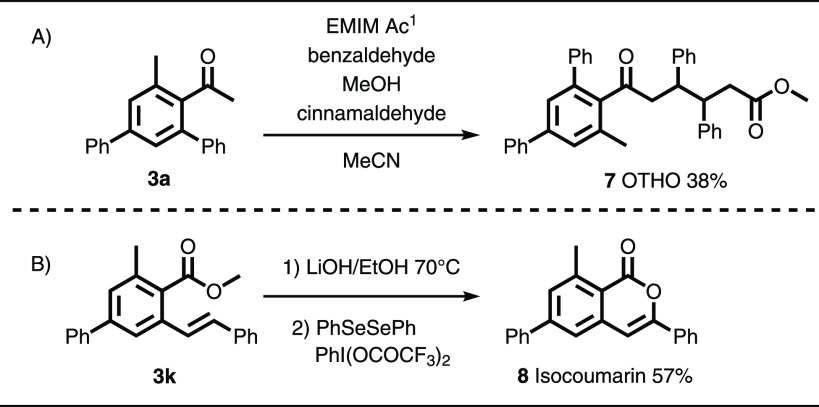
Synthetic Applicability of the Tetrasubstituted Benzene Products EMIM Ac, 1-ethyl-3-methylimidazolium acetate.

In summary, a novel aerobic N-heterocyclic carbene-catalyzed synthetic method, involving γ-carbon activation and a multistep electron transfer process, has been developed. Tetrasubstituted benzenes could easily be synthesized in one reaction step with yields of ≤98%, and the products were isolated in good to excellent yields. This method utilizes aerial oxygen as the terminal oxidant, which makes the reaction more atom economical than previously reported procedures (see the Supporting Information for detailed calculations). In addition, this method benefits from mild conditions and a simple reaction setup. Furthermore, the synthesized products can be readily modified to yield interesting compounds for applied chemistry disciplines, demonstrating the utility of this reaction. These results will pave the way for further development in aerobic oxidative NHC catalysis, seeking new reactivity under aerobic and mild conditions by utilizing ETM systems.

## Data Availability

The data underlying this study are available in the published article and its Supporting Information.

## Supporting Information Available

General information, general synthetic procedure for tetrasubstituted benzene rings **3a–3z**, 1 mmol scale reaction for the synthesis of **3a**, procedure for GC-FID studies, atom economy calculations, additional data from the optimization, general synthetic procedure for enals **1a–1k**, general synthetic procedure for dienones/β-ketoesters **2b–2m**, spectroscopic data of tetrasubstituted benzene rings **3a–3z**, synthesis of OTHO **7**, synthesis of isocoumarin **8**, references, and ^1^H and ^13^C{^1^H} NMR spectra of products **3a–3z**, **7**, and **8** (PDF)
