# Supplementary material for: Aerobic Oxidative N-Heterocyclic Carbene-Catalyzed Formal [3+3] Cyclization for the Synthesis of Tetrasubstituted Benzene Derivatives
Source: Org Lett. 2022 Dec 5;24(49):9146–50. doi: 10.1021/acs.orglett.2c03879 (PMC9764416; doi:10.1021/acs.orglett.2c03879)

Supporting Information For:

Aerobic Oxidative N-Heterocyclic Carbene Catalyzed Formal [3+3] Cyclization for the Synthesis of Tetra-Substituted Benzene Derivatives

Sara Bacaicoa, Ellymay Goossens, Henrik Sundén*

Department of Chemistry and Molecular Biology, University of Gothenburg, Kemivägen 10, 41296, Göteborg, Sweden

*E-mail address: henrik.sunden@chem.gu.se

Table of contents

General information S2
General synthetic procedure of the tetrasubstituted benzene rings (3a-3z) S2
1 mmol scale reaction for the synthesis of 3a S2
Procedure for GC-FID studies S2
Atom Economy calculations (AE) S2
Additional data from the optimization S3
General synthetic procedure of the enals (1a-1k)[1] S4
General synthetic procedure of the dienones/b-ketoesters (2b-2m)[6] S5
Spectroscopic data of the tetrasubstituted benzene rings (3a-3z) S7
Synthesis of OTHO 7 .... S9
Synthesis of isocoumarin 8 .... S10
References S10
^{1}H and ^{13}C {^{1}H} NMR spectroscopy of products 3a-3z, 7 and 8........ S12

General information

All reagents were purchased from VWR, Sigma Aldrich and Thermo Fischer Scientific. All solvents were purchased from VWR. All moisture-sensitive reactions were performed under nitrogen with standard Schlenk techniques. THF and toluene were retrieved from the SPS brand inert, Model number PS-MD-5/7 for anhydrous reactions. Proton nuclear magnetic resonance (^{1}H NMR) spectra and Carbon nuclear magnetic resonance (^{13}C NMR) were recorded on Bruker Avance 400 (400 MHz) spectrometer in $CDCl_3$. ^{1}H NMR and ^{13}C NMR spectra were referenced to residual solvent (for ^{1}H NMR, δ = 7.26 ppm, singlet; for ^{13}C NMR, δ = 77.16 ppm, triplet). High resolution mass spectral analysis (HRMS) was performed on an Agilent 6520 equipped with an electrospray interface and operated in the positive ionization mode. GC-FID spectra were recorded on Agilent Technologies 7820A GC System. Analytical thin-layer chromatography (TLC) was carried out on Merck 60 F254 pre-coated silica gel plate (0.2 mm thickness) and visualized by 254 nm light. For collecting HRMS data the equipment used was Agilent QTOF 6520 equipped with an electrospray interface operated in positive ionization mode.

General synthetic procedure of the tetrasubstituted benzene rings (3a-3z)

To a 5 mL V-vial containing the enone (0.2 mmol), Fe(II)Pc **6** (11.4 mg, 10 mol%), Cs_2CO_3 (130.31 mg, 2 equiv.), dimesityl imidazolium chloride **4a** (13.6 mg, 30 mol%), tetratertbutylbiphenoquinone **5** (8.17 mg, 10 mol%), enal (0.4 mmol, 2 equiv.) was added THF (4 mL). The reaction mixture was stirred during 14 hours in the open V-vial at 25 °C. Solvents were removed under reduced pressure and purified by flash column chromatography (Pentane/Toluene, 3:2) to give the product.

1 mmol scale reaction for the synthesis of 3a

To a 25 mL vial containing the enone **2a** (188.2 mg, 1 mmol), Fe(II)Pc **6** (56.8 mg, 10 mol%), Cs_2CO_3 (651.6 mg, 2 equiv.), dimesityl imidazolium chloride **4a** (102.3 mg, 30 mol%), tetratertbutylbiphenoquinone **5** (40.9 mg, 10 mol%), enal **1a** (292.4 mg, 2 mmol, 2 equiv.) was added THF (20 mL). The reaction mixture was stirred during 14 hours in the open V-vial at 25 °C. Solvents were removed under reduced pressure and purified by flash column chromatography (Pentane/Toluene, 3:2) to give 252 mg of the product **3a** in 88% yield.

Procedure for GC-FID studies

Dodecane was added to the reactions as internal standard. Aliquots of 20 µL were taken from the reaction, diluted with ethyl acetate (1.5 mL) and analyzed using GC-FID from Agilent Technologies. GC-FID method used: 50 °C for 0.2 minutes, 50 °C/min to 220 °C, 10 °C/min to 250, 40 °C/min to 300 °C. The following retention times (ret.t.) were observed: Internal standard dodecane ret.t.: 3.67. Product 1-(5'-Methyl-[1,1':3',1''-terphenyl]-4'-yl)ethanone (**3a**) ret.t.: 9.14.

The response factors (F) were calculated using the following relationship:

$$\frac{A_X}{[X]} = F\,\frac{A_S}{[S]}$$

A_X = Area of the peak corresponding to the analyte
[X] = Concentration of the analyte¨
F = Response factor
A_S = Area of the peak corresponding to the internal standard
[S] = Concentration of the internal standard

Atom Economy calculations (AE)

$$AE = \frac{MW_{product}}{\Sigma MW_{starting\ materials}} \times 100$$

Atom Economy using atmospheric oxygen as terminal oxidant:

$$AE = \frac{MW(\boldsymbol{3a})}{MW(O_2) + MW(\boldsymbol{2a}) + MW(\boldsymbol{1a}) + MW(Cs_2CO_3)} \times 100 = \frac{286,37\ g/mol}{(32.00 + 188.22 + 146.19 + 325.82)g/mol} \times 100 = 41.36\ \%$$

Atom Economy using stoichiometric amount of **5** as oxidant:

$$AE = \frac{MW(\mathbf{3a})}{[MW(\mathbf{5})] \times 2 + MW(\mathbf{2a}) + MW(\mathbf{1a}) + MW(Cs_2CO_3)} \times 100 = \frac{286{,}37\ g/mol}{(408.62 \times 2 + 188.22 + 146.19 + 325.82) g/mol} \times 100 = 19.38\ \%$$

Additional data from the optimization

Enal (**1a**) and dienone (**2a**) were used as model substrates for the optimization of the reaction (Scheme 1).

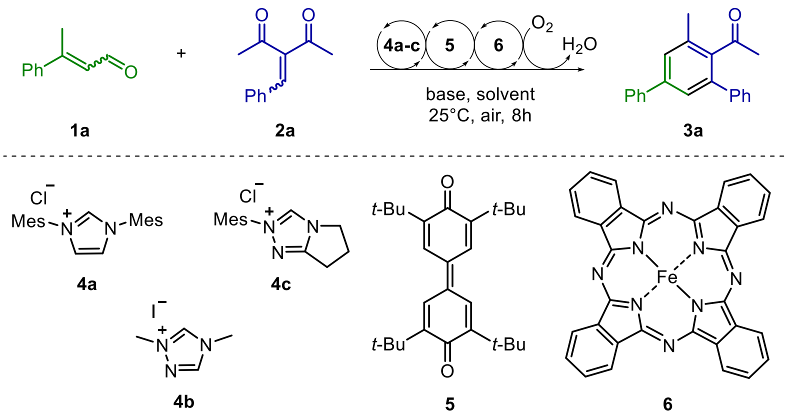

Scheme S1. Overview of reagents and reactants utilised in the optimisation.

Table S1. Evaluating different NHC catalysts.

Entry [a]	NHC (mol%)	Yield (%) [b]
**2**	**4a** (5)	29
**3**	**4b** (5)	1
**4**	**4c** (5)	n.r.

[a]Reaction conditions: **2a** (0.2 mmol), **1a** (0.2 mmol, 1 equiv.), **6** (0.02 mmol, 10 mol%), **5** (0.02 mmol, 10 mol%), **4a-c** (0.01 mmol, 5 mol%), cesium carbonate (0.3 mmol, 1.5 equiv.), THF (2 mL), 25 °C, air and 8 h. Solvent was manually kept at the same level. [b] Determined by GC-FID. n.r. = no reaction.

Table S2. Evaluating different bases.

Entry [a]	Base (equiv.)	Yield (%) [b]
**5**	Cs_2CO_3 (1.5)	47
**6**	*t*BuOK (1.5)	<1
**7**	TBD (1.5)	<1
**8**	DBU (1.5)	n.r.

[a]Reaction conditions: **2a** (0.2 mmol), **1a** (0.2 mmol, 1 equiv.), **6** (0.02 mmol, 10 mol%), **5** (0.02 mmol, 10 mol%), **4a** (0.02 mmol, 10 mol%), base (0.3 mmol, 1.5 equiv.), THF (2 mL), 25 °C, air and 8 h. Solvent was manually kept at the same level. [b] Determined by GC-FID. n.r. = no reaction. [b] Determined by GC-FID. n.r. = no reaction. TBD: Triazabicyclodecene. DBU: 1,8-diazabicyclo[5.4.0]undec7-ene.

Table S3. Evaluating different solvents.

Entry [a]	Solvent (mL)[b]	Yield (%) [c]
**5**	THF (2)	47
**9**	EtOAc (2)	17
**10**	MeCN (2)	n.r.
**11**	$CHCl_3$ (2)	32
**12**	DCM (2)	24
**13**	MeOH (2)	n.r.

[a]Reaction conditions: **2a** (0.2 mmol), **1a** (0.2 mmol, 1 equiv.), **6** (0.02 mmol, 10 mol%), **5** (0.02 mmol, 10 mol%), **4a** (0.02 mmol, 10 mol%), cesium carbonate (0.3 mmol, 1.5 equiv.), solvent (2 mL), 25 °C, air and 8 h. [b]Solvent was manually kept at the same level. [c] Determined by GC-FID. n.r. = no reaction.

Table S4. Evaluating the amount of solvent.

Entry [a]	Solvent (mL)	Yield (%)
**16**	THF (2)[b]	76[c]
**17**	THF (4)	76[d]

[a]Reaction conditions: **2a** (0.2 mmol), **1a** (0.4 mmol, 2 equiv.), **6** (0.02 mmol, 10 mol%), **5** (0.02 mmol, 10 mol%), **4a** (0.06 mmol, 30 mol%), cesium carbonate (0.4 mmol, 2 equiv.), solvent, 25 °C, air and 14 h. [b]Solvent was manually kept at the same level. [c] Determined by GC-FID. [d]Isolated yield. n.r. = no reaction.

General synthetic procedure of the enals (1a-1k)[1]

In an oven-dried 100 mL Schlenk flask, 40% ethoxy acetylene solution in hexanes (2.5 equiv., 10 mmol, 2.4 mL) was added under nitrogen atmosphere. The solution was diluted in THF (1.4 mL) and cooled down to 0 °C. 2M solution of borane dimethylsulfide in THF (0.5 equiv., 2 mmol, 1 mL) was added dropwise to the solution of ethoxy acetylene over 30 min at 0 °C. The reaction was warmed to room temperature and stirred for 12 h, then heated to 60 °C for 1 h. When the reaction was cooled down to room temperature, the volatile materials were removed under reduced pressure. The Schlenk flask was put under nitrogen atmosphere and the residue was dissolved in dry toluene (3.8 mL). The reaction was cooled down to -78°C in a bath of dry ice and acetone and 1.1M solution of diethylzinc in toluene (1.8 equiv., 7.2 mmol, 6.5 mL) was added dropwise. After the mixture was stirred for 20 min at -78°C, an acetophenone (1 equiv., 4 mmol) was added. The reaction was warmed to room temperature for 2 hours and stirred for 48 h. After completion of the reaction, it was cooled down to 0 °C and diluted with Et_2O (7 mL) and quenched with brine (20 mL). After 10 min of vigorous stirring 2M HCl solution was added dropwise until the solids were dissolved and the pH of the aqueous layer was below 4. The mixture was then stirred for 10 min and monitored by TLC. After completion the aqueous and organic layer were separated, and the organic layer was washed with brine and dried over Na_2SO_4. The volume was removed under reduced pressure at a temperature under 30°C and the crude was purified using flash column chromatography (Pentane/Ethyl acetate, 4:1) to afford the enal. It's worth noting that these kinds of aldehydes are unstable and need to be stored under nitrogen and in a -80°C freezer.

3-phenyl-2-butenal (1a)[1]

Purified using flash column chromatography (Pentane/Ethyl acetate, 4:1) Product obtained as a light yellow oil.
^{1}H NMR (400 MHz, $CDCl_3$) δ = 10.18 (d, *J* = 7.9 Hz, 1H), 7.60 – 7.50 (m, 2H), 7.47 – 7.36 (m, 3H), 6.40 (d, *J* = 7.9 Hz, 1H), 2.58 (s, 3H). **^{13}C{^{1}H} NMR (101 MHz, $CDCl_3$) δ =** 191.4, 157.8, 140.6, 130.2, 128.8, 127.3, 126.3, 16.5.

3-(3-bromophenyl)-2-butenal (1b)[2]

Purified using flash column chromatography (Pentane/Ethyl acetate, 4:1) Product obtained as a light yellow solid.
^{1}H NMR (400 MHz, $CDCl_3$) δ = 10.17 (d, *J* = 7.8 Hz, 1H), 7.67 (t, *J* = 1.9 Hz, 1H), 7.55 (dt, *J* = 7.9, 1.9, 1H), 7.46 (dt, *J* = 7.9, 1.9, 1H), 7.29 (t, *J* = 7.9 Hz, 1H), 6.35 (dt, *J* = 7.8, 1.3 Hz, 1H), 2.55 (s, 3H). **^{13}C{^{1}H} NMR (101 MHz, $CDCl_3$) δ =**190.8, 155.5, 142.5, 132.6, 130.1, 129.16, 127.7, 124.7, 26.2, 16.2.

3-(4-Chlorophenyl)-2-butenal (1c)[3]

Purified using flash column chromatography (Pentane/Ethyl acetate, 4:1) Product obtained as a light yellow oil.
^{1}H NMR (400 MHz, $CDCl_3$) δ = 10.16 (d, *J* = 7.8 Hz, 1H), 7.49 – 7.45 (m, 2H), 7.40 – 7.35 (m, 2H), 6.35 (d, *J* = 7.8, 1H), 2.54 (s, 3H). **^{13}C{^{1}H} NMR (101 MHz, $CDCl_3$) δ =** 190.8, 155.8, 138.7, 135.8, 128.7, 127.4, 127.1, 16.0.

3-(3-chloro)-phenyl-2-butenal (1d)[2]

Purified using flash column chromatography (Pentane/Ethyl acetate, 4:1) Product obtained as a light yellow oil.
^{1}H NMR (400 MHz, $CDCl_3$) δ = 10.17 (d, *J* = 7.7 Hz, 1H), 7.50 (s, 1H), 7.45 – 7.28 (m, 3H), 6.35 (d, *J* = 7.7 Hz, 1H), 2.54 (s, 3H). **^{13}C{^{1}H} NMR (101 MHz, $CDCl_3$) δ =** 191.1, 156.0, 142.5, 134.9, 130.1, 130.0, 128.0, 126.6, 124.5, 16.5.

3-(2-chloro)-phenyl-2-butenal (1e)[4]

Purified using flash column chromatography (Pentane/Ethyl acetate, 4:1) Product obtained as a light yellow oil.
^{1}H NMR (400 MHz, $CDCl_3$) δ = 10.17 (d, *J* = 7.8 Hz, 1H), 7.48 – 7.35 (m, 1H), 7.36 – 7.25 (m, 3H), 7.24 – 7.13 (m, 1H), 6.01 (d, *J* = 7.8, 1H), 2.52 (s, 3H). **^{13}C{^{1}H} NMR (101 MHz, $CDCl_3$) δ =** 191.1, 141.6, 130.7, 130.3, 130.1, 129.7, 129.6, 128.6, 127.0, 18.6.

3-(2-naphthyl)-2-butenal (1f)[2]

Purified using flash column chromatography (Pentane/Ethyl acetate, 4:1) Product obtained as a light yellow oil.
^{1}H NMR (400 MHz, $CDCl_3$) δ = 10.24 (d, *J* = 7.8 Hz, 1H), 8.05 – 8.00 (m, 1H), 7.92 – 7.81 (m, 3H), 7.64 (dt, *J* = 8.7, 2.1 Hz, 1H), 7.57 – 7.49 (m, 2H), 6.55 (d, *J* = 7.8 Hz, 1H), 2.68 (s, 3H). **^{13}C{^{1}H} NMR (101 MHz, $CDCl_3$) δ =** 191.2, 157.2, 137.6, 134.0, 133.0, 128.7, 128.5, 127.6, 127.5, 127.3, 126.8, 126.4, 123.4, 16.3.

3-(3-Methylphenyl)-2-butenal (1g)[5]

Purified using flash column chromatography (Pentane/Ethyl acetate, 4:1) Product obtained as a light yellow oil.
^{1}H NMR (400 MHz, $CDCl_3$) δ = 10.18 (d, *J* = 7.9 Hz, 1H), 7.38 – 7.20 (m, 4H), 6.39 (dd, *J* = 7.9, 1.3 Hz, 1H), 2.56 (d, *J* = 1.3 Hz, 3H), 2.39 (s, 3H). **^{13}C{^{1}H} NMR (101 MHz, $CDCl_3$) δ =** 191.4, 158.0, 140.6, 138.5, 130.9, 128.7, 127.30, 127.09, 123.5, 21.6, 16.5.

3-(2-Thienyl)-2-butenal (1h)[2]

Purified using flash column chromatography (Pentane/Ethyl acetate, 3:1) Product obtained as a light brown solid.
^{1}H NMR (400 MHz, $CDCl_3$) δ = 10.08 (d, *J* = 7.8 Hz, 1H), 7.44 – 7.37 (m, 2H), 7.06 (t, *J* = 4.4 Hz, 1H), 6.42 (d, *J* = 7.8 Hz, 1H), 2.54 (s, 3H). **^{13}C{^{1}H} NMR (101 MHz, $CDCl_3$) δ =** 190.7, 133.8, 132.6, 129.1, 128.4, 127.9, 124.4, 16.1

3-(2-Furanyl)-2-butenal (1i)[2]

Purified using flash column chromatography (Pentane/Ethyl acetate, 3:1) Product obtained as a light yellow solid.
^{1}H NMR (400 MHz, $CDCl_3$) δ = 10.13 (d, *J* = 8.1 Hz, 1H), 7.54 (d, *J* = 1.8 Hz, 1H), 6.81 (d, *J* = 3.5 Hz, 1H), 6.55 (dd, *J* = 8.1, 1.3 Hz, 1H), 6.52 (dd, *J* = 3.5, 1.8 Hz, 1H), 2.44 (d, *J* = 1.3 Hz, 3H). **^{13}C{^{1}H} NMR (101 MHz, $CDCl_3$) δ =** 190.9, 153.5, 145.2, 144.1, 122.4, 113.2, 112.5, 13.5.

3-(4-Methoxyphenyl)-2-butenal (1j)[2]

Purified using flash column chromatography (Pentane/Ethyl acetate, 4:1) Product obtained as a yellow oil.
^{1}H NMR (400 MHz, $CDCl_3$) δ = 10.15 (d, *J* = 8.0 Hz, 1H), 7.54 (d, *J* = 8.9 Hz, 2H), 6.93 (d, *J* = 8.9 Hz, 2H), 6.39 (d, *J* = 8.0 Hz, 1H), 3.85 (s, 3H), 2.55 (s, 3H). **^{13}C{^{1}H} NMR (101 MHz, $CDCl_3$) δ =** 193.4, 161.4, 157.1, 132.5, 127.9, 125.7, 114.2, 55.5, 16.2.

3-(4-Methylphenyl)-2-butenal (1k)[2]

Purified using flash column chromatography (Pentane/Ethyl acetate, 4:1) Product obtained as a yellow oil.
^{1}H NMR (400 MHz, $CDCl_3$) δ = 10.16 (d, *J* = 7.9 Hz, 1H), 7.45 (d, *J* = 8.2 Hz, 2H), 7.21 (d, *J* = 8.2 Hz, 2H), 6.39 (d, *J* = 7.9 Hz, 1H), 2.53 (s, 3H), 2.37 (s, 3H). **^{13}C{^{1}H} NMR (101 MHz, $CDCl_3$) δ =** 191.3, 157.6, 140.6, 137.5, 129.5, 126.5, 126.2, 21.3, 16.2.

3-phenyl-2-pentenal (1l)[2]

Purified using flash column chromatography (Pentane/Ethyl acetate, 4:1) Product obtained as a yellow oil.
^{1}H NMR (400 MHz, $CDCl_3$) δ = 10.16 (d, *J* = 8.0 Hz, 1H), 7.55 – 7.46 (m, 2H), 7.46 – 7.37 (m, 3H), 6.26 (d, *J* = 8.0 Hz, 1H), 3.07 (q, *J* = 7.6 Hz, 2H), 1.18 (t, *J* = 7.6 Hz, 3H). **^{13}C{^{1}H} NMR (101 MHz, $CDCl_3$) δ =** 191.1, 164.8, 139.5, 130.0, 128.9, 127.0, 126.7, 23.3, 15.1.

General synthetic procedure of the dienones/b-ketoesters (2b-2m)[6]

To a 5 mL V-vial, corresponding aldehyde (6 mmol), ketone (6 mmol) and L-proline (10 mol%) were added and stirred overnight at room temperature. After a basic work-up with $NaHCO_3$, volume was reduced and the crude product was purified by flash column chromatography (Pentane/Ethyl acetate, 4:1) to obtain the product.

3-[(4-Methylphenyl)methylene]-2,4-pentanedione (2b)[7]

Purified using flash column chromatography (Pentane/Ethyl acetate, 4:1) Product obtained as a yellow oil.
^{1}H NMR (400 MHz, $CDCl_3$) δ = 7.45 (s, 1H), 7.29 (d, *J* = 8.2 Hz, 2H), 7.20 (d, *J* = 8.2 Hz, 2H), 2.42 (s, 3H), 2.38 (s, 3H), 2.30 (s, 3H). **^{13}C{^{1}H} NMR (101 MHz, $CDCl_3$) δ =** 206.0, 196.6, 142.0, 141.5, 140.0, 129.8, 31.7, 26.5, 21.5.

3-[(3-Bromophenyl)methylene]-2,4-pentanedione (2c)[7]

Purified using flash column chromatography (Pentane/Ethyl acetate, 4:1) Product obtained as a light yellow oil.

^{1}H NMR (400 MHz, $CDCl_3$) δ = 7.56 – 7.48 (m, 2H), 7.37 (s, 1H), 7.34 – 7.20 (m, 2H), 2.40 (s, 3H), 2.27 (s, 3H). **^{13}C{^{1}H} NMR (101 MHz, $CDCl_3$) δ =** 205.0, 196.3, 143.9, 137.9, 135.0, 133.5, 132.6, 130.6, 127.9, 123.1, 31.7, 26.6.

3-(4-Chlorobenzylidene)-2,4-pentanedione (2d)[7]
Purified using flash column chromatography (Pentane/Ethyl acetate, 4:1) Product obtained as a light yellow solid.
^{1}H NMR (400 MHz, $CDCl_3$) δ = 7.42 (s, 1H), 7.37 (d, J = 8.6 Hz, 2H), 7.33 (d, J = 8.6 Hz, 2H), 2.42 (s, 3H), 2.29 (s, 3H). **^{13}C{^{1}H} NMR (101 MHz, $CDCl_3$) δ =** 205.4, 196.4, 143.2, 138.3, 136.8, 131.4, 130.9, 129.4, 31.7, 26.5.

3-[(2-Fluorophenyl)methylene]-2,4-pentanedione (2e)[8]
Purified using flash column chromatography (Pentane/Ethyl acetate, 4:1) Product obtained as a yellow solid.
^{1}H NMR (400 MHz, $CDCl_3$) δ = 7.64 (s, 1H), 7.46 – 7.30 (m, 2H), 7.18 – 7.08 (m, 2H), 2.45 (s, 3H), 2.29 (s, 3H). **^{13}C{^{1}H} NMR (101 MHz, $CDCl_3$) δ =** 204.7, 196.6, 161.9, 159.4, 144.0, 132.5 (d, J = 8.8 Hz), 132.0 (d, J = 5.1 Hz), 123.0 (d, J = 1.9 Hz), 124.6 (d, J = 3.8 Hz), 116.0 (d, J = 21.6 Hz), 31.4, 26.5.

3-(2-Thienylmethylene)-2,4-pentanedione (2f)[8]
Purified using flash column chromatography (Pentane/Ethyl acetate, 4:1) Product obtained as a light orange oil.
^{1}H NMR (400 MHz, $CDCl_3$) δ = 7.54 (d, J = 5.1 Hz, 1H), 7.52 (s, 1H), 7.33 (d, J = 3.7 Hz, 1H), 7.10 (dd, J = 5.1, 3.7 Hz, 1H), 2.43 (s, 3H), 2.41 (s, 3H). **^{13}C{^{1}H} NMR (101 MHz, $CDCl_3$) δ=** 205.2, 196.5, 139.3, 136.0, 134.3, 132.6, 132.3, 128.3, 31.4, 26.2.

3-(2-Naphthalenylmethylene)-2,4-pentanedione (2g)[9]
Purified using flash column chromatography (Pentane/Ethyl acetate, 4:1) Product obtained as a light yellow solid.
^{1}H NMR (400 MHz, $CDCl_3$) δ = 7.91 (s, 1H), 7.88 – 7.80 (m, 3H), 7.65 (s, 1H), 7.60 – 7.49 (m, 2H), 7.49 – 7.41 (m, 1H), 2.47 (s, 3H), 2.32 (s, 3H). **^{13}C{^{1}H} NMR (101 MHz, $CDCl_3$) δ =** 205.9, 196.6, 142.9, 140.0, 134.1, 133.1, 131.0, 130.4, 129.0, 128.8, 128.0, 127.9, 127.1, 125.7, 31.9, 26.7.

3-[3-Phenyl-2-propen-1-ylidene]-2,4-pentanedione (2h)[6]
Purified using flash column chromatography (Pentane/Ethyl acetate, 4:1) Product obtained as a light orange solid.
^{1}H NMR (400 MHz, $CDCl_3$) δ = 7.53 – 7.46 (m, 2H), 7.42 – 7.31 (m, 3H), 7.27 – 7.03 (m, 3H), 2.42 (s, 3H), 2.41 (s, 3H). **^{13}C{^{1}H} NMR (101 MHz, $CDCl_3$) δ =** 199.1, 193.3, 141.1, 139.0, 137.5, 131.5, 126.1, 125.0, 123.9, 119.4, 27.9, 22.4.

3-oxo-2-(phenylmethylene)butanoate (2i)[6]
Purified using flash column chromatography (Pentane/Ethyl acetate, 4:1) Product obtained as a white solid.
^{1}H NMR (400 MHz, $CDCl_3$) δ = 7.58 (s, 1H), 7.48 – 7.35 (m, 4H), 3.85 (s, 3H), 2.43 (s, 3H). **^{13}C{^{1}H} NMR (101 MHz, $CDCl_3$) δ =** 194.6, 167.9, 141.3, 134.1, 132.5, 130.6, 129.1, 128.7, 52.2, 25.9.

Methyl 2-acetyl-5-phenyl-2,4-pentadienoate (2j)[6]
Purified using flash column chromatography (Pentane/Ethyl acetate, 4:1) Product obtained as a light yellow solid.
^{1}H NMR (400 MHz, $CDCl_3$) δ = 7.54 – 7.22 (m, 7H), 7.15 – 7.05 (m, 1H), 3.91 (s, 3H), 2.40 (s, 3H). **^{13}C{^{1}H} NMR (101 MHz, $CDCl_3$) δ =** 195.6, 166.8, 146.1, 145.2, 135.6, 132.1, 130.1, 129.0, 128.0, 123.7, 52.2, 28.0.

Methyl 2-[(4-methoxyphenyl)methylene]-3-oxobutanoate (2k)[10]
Purified using flash column chromatography (Pentane/Ethyl acetate, 4:1) Product obtained as a light yellow solid.
^{1}H NMR (400 MHz, $CDCl_3$) δ = 7.51 (s, 1H), 7.39 (d, J = 9.0 Hz, 2H), 6.90 (d, J = 9.0 Hz, 2H), 3.86 (s, 3H), 3.84 (s, 3H), 2.39 (s, 3H). **^{13}C{^{1}H} NMR (101 MHz, $CDCl_3$) δ =** 194.8, 168.8, 161.9, 141.5, 132.0, 131.8, 125.2, 114.6, 55.5, 52.6, 26.4.

Methyl 2-[(4-chlorophenyl)methylene]-3-oxobutanoate (2l)[10]
Purified using flash column chromatography (Pentane/Ethyl acetate, 4:1) Product obtained as a white solid.
^{1}H NMR (400 MHz, $cdcl_3$) δ = 7.53 (s, 1H), 7.41 – 7.33 (m, 4H), 3.85 (s, 3H), 2.42 (s, 3H). **^{13}C{^{1}H} NMR (101 MHz, $CDCl_3$) δ =** 194.4, 168.1, 140.2, 137.0, 134.7, 131.4, 130.7, 129.4, 52.8, 26.7.

4-benzylideneheptane-3,5-dione (2m)[11]
Purified using flash column chromatography (Pentane/Ethyl acetate, 4:1) Product obtained as a light yellow oil.
^{1}H NMR (400 MHz, $CDCl_3$) δ = 7.54 (s, 1H), 7.44 – 7.30 (m, 5H), 2.75 (q, J = 7.2 Hz, 2H), 2.49 (q, J = 7.2 Hz, 2H), 1.15 (t, J = 7.2 Hz, 3H), 1.08 (t, J = 7.2 Hz, 3H). **^{13}C{^{1}H} NMR (101 MHz, $CDCl_3$) δ** = 208.8, 199.0, 142.3, 138.6, 133.2, 130.4, 129.5, 129.0, 37.4, 32.0, 8.0, 7.6.

Spectroscopic data of the tetrasubstituted benzene rings (3a-3z)

1-(5'-Methyl-[1,1':3',1''-terphenyl]-4'-yl)ethanone (3a)[12]
Purified using flash column chromatography (Pentane/Toluene, 3:2) Product obtained as a light yellow oil, yield 76%, 43.5 mg. **^{1}H NMR (400 MHz, $CDCl_3$) δ =** 7.67 – 7.59 (m, 2H), 7.50 – 7.34 (m, 10H), 2.41 (s, 3H), 1.97 (s, 3H). **^{13}C{^{1}H} NMR (101 MHz, $CDCl_3$) δ =** 207.6, 141.8, 140.5, 140.25, 140.24 139.3, 134.5, 129.0, 128.9, 128.7, 128.4, 127.9, 127.8, 127.2, 126.2, 32.2, 19.8.

1-(2''-Fluoro-5'-methyl-[1,1':3',1''-terphenyl]-4'-yl)ethanone (3b)[9]
Purified using flash column chromatography (Pentane/Toluene, 3:2) Product obtained as a light yellow oil, yield 70%, 42.5 mg. **^{1}H NMR (400 MHz, $CDCl_3$) δ =** 7.65 – 7.59 (m, 2H), 7.51 – 7.27 (m, 7H), 7.24 – 7.12 (m, 2H), 2.43 (s, 3H), 2.09 (s, 3H). **^{13}C{^{1}H} NMR (101 MHz, $CDCl_3$) δ =** 206.5, 159.5 (d, *J* = 246.8 Hz), 141.7, 140.9, 140.1, 134.6, 132.9, 132.1 (d, *J* = 3.0 Hz), 130.1 (d, *J* = 8.0 Hz), 129.2, 129.0, 127.9, 127.8, 127.3, 127.2 (d, *J* = 1.6 Hz), 124.4 (d, *J* = 3.8 Hz), 116.0 (d, *J* = 22.2 Hz), 31.8, 20.1.

1-(3''-Bromo-5'-methyl-[1,1':3',1''-terphenyl]-4'-yl)ethanone (3c)[9]
Purified using flash column chromatography (Pentane/Toluene, 3:2) Product obtained as a light yellow oil, yield 98%, 71.7 mg. **^{1}H NMR (400 MHz, $CDCl_3$) δ =** 7.69 – 7.56 (m, 2H), 7.56 – 7.26 (m, 9H), 2.39 (s, 3H), 2.02 (s, 3H). **^{13}C{^{1}H} NMR (101 MHz, $CDCl_3$) δ =** 207.2, 142.6, 142.1, 140.3, 140.1, 137.7, 134.7, 131.9, 131.1, 130.2, 129.04, 129.00, 128.0, 127.9, 127.3, 126.2, 122.9, 32.4, 19.9.

1-(5'-ethyl-[1,1':3',1''-terphenyl]-4'-yl)propan-1-one (3d)[11]
Purified using flash column chromatography (Pentane/Toluene, 3:2) Product obtained as a light yellow oil, yield 46%, 28.2 mg. **^{1}H NMR (400 MHz, $CDCl_3$) δ =** 7.67 – 7.60 (m, 2H), 7.52 – 7.33 (m, 10H), 2.66 (q, *J* = 7.6 Hz, 2H), 2.20 (q, *J* = 7.2 Hz, 2H), 1.31 (t, *J* = 7.5 Hz, 3H), 0.85 (t, *J* = 7.2 Hz, 3H). **^{13}C{^{1}H} NMR (101 MHz, $CDCl_3$) δ =** 210.7, 141.9, 141.1, 140.7, 140.6, 140.1, 139.3, 129.2, 129.0, 128.7, 127.9, 127.8, 127.4, 126.9, 126.4, 38.4, 26.7, 16.3, 8.0.

1-(4''-Chloro-5'-methyl-[1,1':3',1''-terphenyl]-4'-yl)ethanone (3e)[12]
Purified using flash column chromatography (Pentane/Toluene, 3:2) Product obtained as a light yellow oil, yield 86%, 55.4 mg. **^{1}H NMR (400 MHz, $CDCl_3$) δ =** 7.65 – 7.59 (m, 2H), 7.50 – 7.31 (m, 9H), 2.40 (s, 3H), 2.01 (s, 3H). **^{13}C{^{1}H} NMR (101 MHz, $CDCl_3$) δ =** 207.5, 142.0, 140.3, 140.2, 138.9, 137.9, 134.7, 134.2, 130.4, 129.0, 128.89, 128.88, 128.0, 127.3, 126.1, 32.4, 19.9.

1-(4'',5'-Dimethyl-[1,1':3',1''-terphenyl]-4'-yl)ethanone (3f)[9]
Purified using flash column chromatography (Pentane/Toluene, 3:2) Product obtained as a light yellow oil, yield 54%, 32.3 mg. **^{1}H NMR (400 MHz, $CDCl_3$) δ** = 7.67 – 7.60 (m, 2H), 7.50 – 7.36 (m, 5H), 7.32 (d, *J* = 8.1 Hz, 2H), 7.25 (d, *J* = 7.9 Hz, 2H), 2.42 (s, 3H), 2.41 (s, 3H), 2.00 (s, 3H). **^{13}C{^{1}H} NMR (101 MHz, $CDCl_3$) δ** = 207.8, 141.8, 140.4, 140.3, 139.4, 137.8, 137.6, 134.6, 129.5, 129.0, 128.9, 128.2, 127.8, 127.3, 126.3, 32.3, 21.3, 19.9.

1-(3-Methyl-5-(thiophen-2-yl)-[1,1'-biphenyl]-4-yl)ethanone (3g)[9]
Purified using flash column chromatography (Pentane/Toluene, 3:2) Product obtained as a light yellow oil, yield 85%, 47.9 mg. **^{1}H NMR (400 MHz, $CDCl_3$) δ =** 7.67 – 7.59 (m, 2H), 7.56 – 7.34 (m, 6H), 7.10 – 7.03 (m, 2H), 2.39 (s, 3H), 2.15 (s, 3H). **^{13}C{^{1}H} NMR (101 MHz, $CDCl_3$) δ =** 207.6, 142.0, 141.4, 140.3, 140.1, 134.6, 131.5, 128.99, 128.97, 128.1, 128.0, 127.9, 127.3, 126.7, 126.6, 31.9, 19.8.

1-(3-methyl-5-(naphthalen-2-yl)-[1,1'-biphenyl]-4-yl)ethanone (3h)[9]
Purified using flash column chromatography (Pentane/Toluene, 3:2) Product obtained as a light yellow solid, yield 71%, 47.5 mg. **^{1}H NMR (400 MHz, $CDCl_3$) δ =** 7.94 – 7.87 (m, 4H), 7.68 – 7.63 (m, 2H), 7.60 – 7.35 (m, 8H), 2.45 (s, 3H), 1.97 (s, 3H). **^{13}C{^{1}H} NMR (101 MHz, $CDCl_3$) δ** = 207.7, 142.0, 140.5, 140.3, 139.3, 138.0, 134.8, 133.4, 132.8, 129.0, 128.60, 128.57, 128.4, 128.2, 127.9, 127.8, 127.4, 127.1, 126.7, 126.7, 126.6, 32.4, 20.0.

1-(3-methyl-5-styryl-[1,1'-biphenyl]-4-yl)ethanone (3i)[12]
Purified using flash column chromatography (Pentane/Toluene, 3:2) Product obtained as a light yellow solid, yield 60%, 37.5 mg. **^{1}H NMR (400 MHz, $CDCl_3$) δ =** 7.70 – 7.59 (m, 3H), 7.53 – 7.27 (m, 9H), 7.11 – 7.07 (m, 2H), 2.54 (s, 3H), 2.36 (s, 3H). **^{13}C{^{1}H} NMR (101 MHz, $CDCl_3$) δ** = 208.3, 142.0, 140.6, 140.4, 137.0, 134.0, 133.6, 132.4, 129.0, 128.9, 128.6, 128.3, 127.9, 127.3, 126.9, 125.4, 122.6, 33.1, 19.5.

methyl 5'-methyl-[1,1':3',1''-terphenyl]-4'-carboxylate (3j)[12]

Purified using flash column chromatography (Pentane/Toluene, 3:2) Product obtained as a yellow oil, yield 64%, 38.5 mg. **^{1}H NMR (400 MHz, CDCl$_3$) δ =** 7.66 – 7.61 (m, 2H), 7.50 – 7.34 (m, 10H), 3.62 (s, 3H), 2.50 (s, 3H). **^{13}C{^{1}H} NMR (101 MHz, CDCl$_3$) δ** = 170.4, 142.5, 141.1, 140.9, 140.3, 136.3, 132.0, 129.0, 128.5, 128.3, 128.0, 127.9, 127.6, 127.4, 126.3, 52.0, 20.1.

methyl 3-methyl-5-styryl-[1,1'-biphenyl]-4-carboxylate (3k)[12]

Purified using flash column chromatography (Pentane/Toluene, 3:2) Product obtained as a light yellow oil, yield 59%, 38.7 mg. **^{1}H NMR (400 MHz, CDCl$_3$) δ =** 7.74 (s, 1H), 7.66 – 7.61 (m, 2H), 7.54 – 7.35 (m, 8H), 7.33 – 7.28 (m, 1H), 7.22 (d, *J* = 16.1 Hz, 1H), 7.13 (d, *J* = 16.1 Hz, 1H), 3.99 (s, 3H), 2.44 (s, 3H). **^{13}C{^{1}H} NMR (101 MHz, CDCl$_3$) δ =** 170.3, 142.7, 140.6, 137.2, 136.1, 135.8, 131.8, 129.0, 128.8, 128.4, 128.1, 128.0, 127.4, 126.9, 125.8, 122.1, 52.3, 20.1.

methyl 4''-methoxy-5'-methyl-[1,1':3',1''-terphenyl]-4'-carboxylate (3l)[12]

Purified using flash column chromatography (Pentane/Toluene, 3:2) Product obtained as a light yellow solid, yield 50%, 33.8 mg. **^{1}H NMR (400 MHz, CDCl$_3$) δ =** 7.65 – 7.59 (m, 2H), 7.49 – 7.32 (m, 7H), 6.99 – 6.92 (m, 2H), 3.86 (s, 3H), 3.66 (s, 3H), 2.47 (s, 3H). **^{13}C{^{1}H} NMR (101 MHz, CDCl$_3$) δ** = 170.6, 159.2, 142.4, 140.43, 140.41, 136.1, 133.4, 132.1, 129.5, 128.9, 127.9, 127.7, 127.4, 126.2, 113.9, 55.4, 52.1, 20.0.

1-(4''-chloro-5'-methyl-[1,1':3',1''-terphenyl]-4'-carboxylate (3m)[9]

Purified using flash column chromatography (Pentane/Toluene, 3:2) Product obtained as a yellow oil, yield 61%, 40.8 mg. **^{1}H NMR (400 MHz, CDCl$_3$) δ** = 7.64 – 7.57 (m, 2H), 7.48 – 7.31 (m, 9H), 3.64 (s, 3H), 2.47 (s, 3H). **^{13}C{^{1}H} NMR (101 MHz, CDCl$_3$) δ =** 170.2, 142.7, 140.2, 139.7, 139.5, 136.5, 133.8, 131.2, 129.7, 129.0, 128.7, 128.4, 128.0, 127.4, 126.1, 52.2, 20.1.

1-(3,5'-Dimethyl-[1,1':3',1''-terphenyl]-4'-yl)ethanone (3n)[9]

Purified using flash column chromatography (Pentane/Toluene, 3:2) Product obtained as a light yellow oil, yield 96%, 57.9 mg. **^{1}H NMR (400 MHz, CDCl$_3$) δ =** 7.48 – 7.40 (m, 9H), 7.39 – 7.32 (m, 1H), 7.22 – 7.19 (m, 1H), 2.44 (s, 3H), 2.41 (s, 3H), 1.98 (s, 3H). **^{13}C{^{1}H} NMR (101 MHz, CDCl$_3$) δ** = 207.8, 142.0, 140.6, 140.3, 140.2, 139.4, 138.6, 134.6, 129.1, 128.9, 128.8, 128.6, 128.5, 128.1, 128.0, 126.3, 124.4, 32.3, 21.7, 19.9.

1-(4-Methoxy-5'-methyl-[1,1':3',1''-terphenyl]-4'-yl)ethanone (3o)[9]

Purified using flash column chromatography (Pentane/Toluene, 3:2) Product obtained as a light yellow oil, yield 78%, 39.4 mg. **^{1}H NMR (400 MHz, CDCl$_3$) δ =** 7.57 (d, *J* = 8.8 Hz, 2H), 7.48 – 7.34 (m, 7H), 6.99 (d, *J* = 8.8 Hz, 2H), 3.86 (s, 3H), 2.39 (s, 3H), 1.96 (s, 3H). **^{13}C{^{1}H} NMR (101 MHz, CDCl$_3$) δ** = 207.8, 159.6, 141.5, 140.7, 139.8, 139.4, 134.6, 132.8, 129.1, 128.8, 128.4, 128.0, 127.9, 125.9, 114.4, 55.5, 32.3, 20.0.

1-(3-Chloro-5'-methyl-[1,1':3',1''-terphenyl]-4'-yl)ethanone (3p)[9]

Purified using flash column chromatography (Pentane/Toluene, 3:2) Product obtained as a light yellow oil, yield 82%, 52.6 mg. **^{1}H NMR (400 MHz, CDCl$_3$) δ =** 7.62 – 7.59 (m, 1H), 7.52 – 7.48 (m, 1H), 7.45 – 7.31 (m, 9H), 2.40 (s, 3H), 1.97 (s, 3H). **^{13}C{^{1}H} NMR (101 MHz, CDCl$_3$) δ** = 207.6, 142.2, 140.9, 140.4, 140.3, 139.6, 134.9, 134.8, 130.2, 129.1, 128.9, 128.4, 128.1, 127.9, 127.4, 126.3, 125.5, 32.3, 19.9.

1-(4-Chloro-5'-methyl-[1,1':3',1''-terphenyl]-4'-yl)ethanone (3q)[9]

Purified using flash column chromatography (Pentane/Toluene, 3:2) Product obtained as a light yellow solid, yield 75%, 47.7 mg. **^{1}H NMR (400 MHz, CDCl$_3$) δ =** 7.59 – 7.51 (m, 2H), 7.48 – 7.35 (m, 9H), 2.39 (s, 3H), 1.96 (s, 3H). **^{13}C{^{1}H} NMR (101 MHz, CDCl$_3$) δ** = 207.5, 140.60, 140.59, 140.4, 139.5, 138.7, 134.8, 134.0, 129.13, 129.08, 128.8, 128.5, 128.2, 128.1, 126.1, 32.3, 19.9.

1-(2-Chloro-5'-methyl-[1,1':3',1''-terphenyl]-4'-yl)ethanone (3r)[9]

Purified using flash column chromatography (Pentane/Toluene, 3:2) Product obtained as a light yellow oil, yield 40%, 25.5 mg. **^{1}H NMR (400 MHz, CDCl$_3$) δ =** 7.50 – 7.46 (m, 1H), 7.43 – 7.33 (m, 6H), 7.33 – 7.23 (m, 4H), 2.38 (s, 3H), 1.99 (s, 3H).**^{13}C{^{1}H} NMR (101 MHz, CDCl$_3$) δ =** 207.8, 140.6, 140.4, 140.0, 139.7, 138.5, 133.9, 132.5, 131.4, 130.7, 130.2, 129.2, 129.0, 128.8, 128.7, 128.0, 127.1, 32.3, 19.8.

1-(2-Bromo-5'-methyl-[1,1':3',1''-terphenyl]-4'-yl)ethanone (3s)[9]

Purified using flash column chromatography (Pentane/Toluene, 3:2) Product obtained as a light yellow oil, yield 76%, 55.8 mg. **^{1}H NMR (400 MHz, CDCl$_3$) δ =** 7.76 (s, 1H), 7.57 – 7.47 (m, 2H), 7.45 – 7.37 (m, 7H), 7.35 – 7.29 (m, 1H), 2.40 (s, 3H), 1.97 (s, 3H). **^{13}C{^{1}H} NMR (101 MHz, CDCl$_3$) δ=** 207.5, 142.4, 140.9, 140.3, 140.3, 139.5, 134.8, 130.8, 130.5, 130.4, 129.1, 128.9, 128.4, 128.1, 126.2, 125.9, 123.1, 32.2, 19.9.

1-(3-Methyl-5-(naphthalen-2-yl)-[1,1'-biphenyl]-2-yl)ethanone (3t)[9]

Purified using flash column chromatography (Pentane/Toluene, 3:2) Product obtained as a white solid, yield 82%, 55.5 mg. **^{1}H NMR (400 MHz, $CDCl_3$) δ =** 8.09 (s, 1H), 7.97 – 7.84 (m, 3H), 7.81 – 7.74 (m, 1H), 7.61 – 7.35 (m, 9H), 2.44 (s, 3H), 1.99 (s, 3H). **^{13}C{^{1}H} NMR (101 MHz, $CDCl_3$) δ =** 207.8, 141.8, 140.6, 140.4, 139.6, 137.6, 134.8, 133.7, 133.0, 129.2, 128.9, 128.7, 128.4, 128.0, 127.8, 126.6, 126.3, 126.2, 125.5, 32.3, 20.0.

1-(3-Methyl-5-(thiophen-2-yl)-[1,1'-biphenyl]-2-yl)ethanone (3u)[9]

Purified using flash column chromatography (Pentane/Toluene, 3:2) Product obtained as a yellow oil, yield 89%, 52.3 mg. **^{1}H NMR (400 MHz, $CDCl_3$) δ =** 7.50 – 7.30 (m, 9H), 7.13 – 7.07 (m, 1H), 2.37 (s, 3H), 1.94 (s, 3H). **^{13}C{^{1}H} NMR (101 MHz, $CDCl_3$) δ** = 207.4, 143.4, 140.4, 140.3, 139.6, 135.0, 134.9, 129.1, 128.8, 128.3, 128.1, 127.1, 125.6, 124.9, 123.9, 32.2, 19.9.

1-(5-(Furan-2-yl)-3-methyl-[1,1'-biphenyl]-2-yl)ethanone (3v)[9]

Purified using flash column chromatography (Pentane/Toluene, 3:2) Product obtained as a light yellow oil, yield 83%, 46.1 mg. **^{1}H NMR (400 MHz, $CDCl_3$) δ =** 7.58 – 7.47 (m, 3H), 7.45 – 7.30 (m, 5H), 6.72 (dt, *J* = 3.4, 0.9 Hz, 1H), 6.52 – 6.46 (m, 1H), 2.36 (s, 3H), 1.94 (s, 3H). **^{13}C{^{1}H} NMR (101 MHz, $CDCl_3$) δ** = 207.6, 153.2, 142.7, 140.4, 140.2, 139.5, 134.7, 131.3, 129.0, 128.8, 128.0, 124.8, 122.8, 111.9, 106.2, 32.2, 19.9.

(*E*)-1-(3'-methoxy-3-methyl-5-styryl-[1,1'-biphenyl]-4-yl)ethanone (3w)[12]

Purified using flash column chromatography (Pentane/Toluene, 3:2) Product obtained as a light yellow solid, yield 73%, 50.3 mg. **^{1}H NMR (400 MHz, $CDCl_3$) δ =** 7.68 – 7.27 (m, 9H), 7.09 (s, 2H), 7.06 – 6.98 (m, 2H), 3.87 (s, 3H), 2.54 (s, 3H), 2.36 (s, 3H). **^{13}C{^{1}H} NMR (101 MHz, $CDCl_3$) δ** = 208.3, 159.6, 141.5, 139.8, 137.0, 134.0, 133.6, 133.0, 132.2, 128.9, 128.3, 128.22, 128.18, 126.8, 125.6, 122.1, 114.4, 55.5, 33.1, 19.5.

(*E*)-1-(3'-bromo-3-methyl-5-styryl-[1,1'-biphenyl]-4-yl)ethanone (3x)[12]

Purified using flash column chromatography (Pentane/Toluene, 3:2) Product obtained as a light yellow solid, yield 53%, 41.7 mg. **^{1}H NMR (400 MHz, $CDCl_3$) δ =** 7.76 (s, 1H), 7.63 (s, 1H), 7.58 – 7.46 (m, 4H), 7.42 – 7.22 (m, 5H), 7.14 – 7.01 (m, 2H), 2.53 (s, 3H), 2.35 (s, 3H). **^{13}C{^{1}H} NMR (101 MHz, $CDCl_3$) δ** = 208.0, 142.7, 140.9, 140.5, 136.8, 134.1, 133.9, 132.7, 130.8, 130.5, 130.3, 128.9, 128.5, 128.4, 126.9, 126.0, 125.1, 123.1, 122.5, 33.0, 19.5.

(*E*)-1-(3,4'-dimethyl-5-styryl-[1,1'-biphenyl]-4-yl)ethanone (3y)[12]

Purified using flash column chromatography (Pentane/Toluene, 3:2) Product obtained as a light yellow oil, yield 68%, 49.9 mg. **^{1}H NMR (400 MHz, $CDCl_3$) δ =** 8.14 – 8.03 (m, 1H), 7.99 – 7.85 (m, 3H), 7.83 – 7.74 (m, 2H), 7.61 – 7.46 (m, 5H), 7.43 – 7.23 (m, 3H), 7.17 – 7.09 (m, 2H), 2.56 (s, 3H), 2.40 (s, 3H). **^{13}C{^{1}H} NMR (101 MHz, $CDCl_3$) δ** = 208.3, 141.9, 140.4, 137.9, 137.0, 134.1, 133.8, 133.7, 132.9, 132.5, 128.91, 128.86, 128.7, 128.4, 128.3, 127.8, 126.9, 126.6, 126.3, 126.1, 125.52, 125.47, 122.8, 33.1, 19.6.

1-(2-methyl-6-styryl-4-(thiophen-2-yl)phenyl)ethanone (3z)[12]

Purified using flash column chromatography (Pentane/Toluene, 3:2) Product obtained as a light yellow oil, yield 65%, 41.2 mg. **^{1}H NMR (400 MHz, $CDCl_3$) δ =** 7.69 (s, 1H), 7.53 – 7.28 (m, 8H), 7.15 – 7.04 (m, 3H), 2.52 (s, 3H), 2.33 (s, 3H). **^{13}C{^{1}H} NMR (101 MHz, $CDCl_3$) δ** = 207.9, 143.5, 140.4, 136.8, 135.0, 134.2, 133.9, 132.6, 128.9, 128.3, 128.2, 127.2, 126.9, 125.5, 125.2, 123.8, 121.2, 33.0, 19.4.

Synthesis of OTHO 7

To a 5 mL V-vial equipped with a magnetic stirrer, was added: TBD (25.5 mg, 0.18 mmol, 0.5 eq), methanol (211 mg, 6.6 mmol, 18 eq), benzaldehyde (41 μL, 0.4 mmol, 1.1 eq), and **3a** (105 mg, 0.36 mmol, 1 eq). The mixture was stirred at room temperature. A few drops of acetonitrile were added when precipitate started to form. The formation of the chalcone was monitored by TLC and ^{1}H NMR. When the starting material was fully consumed, a mixture of EMIMAc (170 μL, 0.95 mmol) and cinnamaldehyde (138.5 μL, 1.1 mmol) in acetonitrile (3.85 mL) was added and was stirred at room temperature during 48h. The volatiles were removed under pressure and then dissolved in DCM and extracted with brine. The crude product was purified by flash column chromatography (Pentane/Ethyl acetate, 4:1) to obtain the product.

1-(5'-methyl-[1,1':3',1''-terphenyl]-4'-yl)-6-(l1-oxidaneyl)-3,4-diphenylhexane-1,6-dione (7)

Product obtained as a white solid, yield 38%, 74 mg. **^{1}H NMR (400 MHz, $CDCl_3$) δ =** 7.59 (d, *J* = 8.3, 1.4 Hz, 2H), 7.48 – 7.38 (m, 5H), 7.37 (ddd, *J* = 9.8, 4.1, 1.9 Hz, 4H), 7.26 (s, 1H), 7.12 – 6.97 (m, 6H), 6.62 (ddd, *J* = 9.8, 7.6, 2.2 Hz, 4H), 3.48 (s, 3H), 3.32 (ddd, *J* = 10.3, 6.4, 4.0 Hz, 1H), 3.14 (q, *J* = 7.4 Hz, 1H), 2.65 (dd, *J* = 17.3, 10.3 Hz, 1H), 2.38 (d, *J* = 7.8 Hz, 2H), 2.33 (dd, *J* = 17.3, 4.1 Hz, 1H), 1.73 (s, 3H). **^{13}C{^{1}H} NMR (101 MHz, $CDCl_3$) δ** = 207.9, 172.6, 141.8, 140.8, 140.5, 140.4, 140.3, 139.6, 139.5, 135.8, 129.3, 129.1, 129.0, 128.9, 128.6, 128.5, 128.1, 127.90,

127.85, 127.8, 127.3, 126.60, 126.59, 126.1, 51.7, 47.8, 46.9, 45.7, 37.5, 19.2. HRMS (ESI) for $C_{38}H_{34}O_3$ [M+H$^+$]: 539.2586, found 539.2585. **IR (ATR):** 3028, 1734, 1685, 1597, 1495, 1452, 1253, 1153, 1076, 1029, 981, 880, 761, 607, 578, 555 cm^{-1}. Melting point: 67.1 °C – 68.6 °C.

Synthesis of isocoumarin 8

To **3k** (38 mg, 0.116 mmol) in 3 mL of MeOH/H_2O (5:1) was added LiOH (20 mg, 0.835 mmol) at room temperature. The reaction mixture was then heated at 70°C for 12 h. After which the reaction mixture was allowed to cool to room temperature and then neutralized (pH = 6) using 1M HCl. The product was extracted with ethyl acetate and washed with brine. The extract was dried with anhydrous $MgSO_4$, evaporated under reduced pressure, and the obtained **3k´** was used directly in the next step without purification.

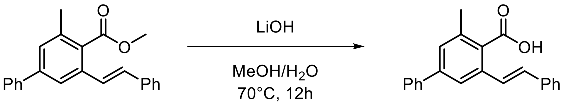

3k 3k´

Scheme S2. Hydrolysis of the ester 3k for the synthesis of the carboxylic acid 3k'.

Crude **3k´** (38 mg, 0.121 mmol) was added to a solution of diphenyl diselenide (0.006 mmol, 2 mg, 10 mol%) in acetonitrile (1 mL) followed by [bis(trifluoroacetoxy)iodo]benzene (28.7 mg, 0.067 mmol) and the mixture was stirred under nitrogen at room temperature until TLC showed no remaining starting material. The solvent was evaporated under reduced pressure and the residue purified immediately by flash chromatography (ethyl acetate/pentane, 1:4) to obtain product **8**, 21.4 mg, 57% yield.[13]

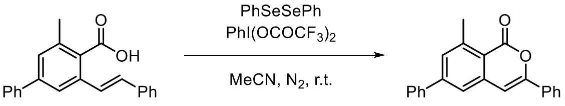

3k´ 8

Scheme S3. Cyclization of 3k' for the synthesis of isocoumarin 8.

8-methyl-3,6-diphenyl-1H-isochromen-1-one (8)
Product obtained as a white solid, yield 57%, 21.4 mg. **^{1}H NMR (400 MHz, $CDCl_3$) δ =** 7.93 – 7.85 (m, 2H), 7.70 – 7.63 (m, 2H), 7.55 – 7.38 (m, 8H), 6.95 (s, 1H), 2.92 (s, 3H). **^{13}C{^{1}H} NMR (101 MHz, $CDCl_3$) δ =** 161.7, 153.6, 146.8, 144.2, 139.7, 139.5, 132.1, 130.1, 130.0, 129.1, 128.9, 128.7, 127.5, 125.3, 122.5, 117.9, 102.6, 23.5. HRMS (ESI) for $C_{22}H_{16}O_2$ [M+H$^+$]: 313.1229, found 313.1229. **IR (ATR):** 2986, 2923, 1718, 1631, 1593, 1559, 1507, 1448, 1381, 1354, 1331, 1283, 1261, 1245, 1210, 1157, 1056, 1030, 1007, 885, 877, 819, 792, 767, 687, 610 cm^{-1}. **Melting point:** 194.2 °C – 195.8 °C.

References

1. Valenta, P.; Drucker, N. A.; Bode, J. W.; Walsh, P. J., Simple One-pot Conversion of Aldehydes and Ketones to Enals. *Org. Lett.* **2009,** *11* (10), 2117-2119.
2. Wang, W.; Yu, Y.; Cheng, B.; Fang, H.; Zhang, X.; Qian, H.; Ma, S., Stereodefined rhodium-catalysed 1,4-H/D delivery for modular syntheses and deuterium integration. *Nature Catalysis* **2021,** *4* (7), 586-594.
3. Li, Y.; Barløse, C.; Jørgensen, J.; Carlsen, B. D.; Jørgensen, K. A., Asymmetric Catalytic Aza-Diels–Alder/Ring-Closing Cascade Reaction Forming Bicyclic Azaheterocycles by Trienamine Catalysis. *Chemistry – A European Journal* **2017,** *23* (1), 38-41.
4. Bräuer, T. M.; Zhang, Q.; Tiefenbacher, K., Iminium Catalysis inside a Self-Assembled Supramolecular Capsule: Scope and Mechanistic Studies. *Journal of the American Chemical Society* **2017,** *139* (48), 17500-17507.
5. Song, A.; Zhang, X.; Song, X.; Chen, X.; Yu, C.; Huang, H.; Li, H.; Wang, W., Construction of Chiral Bridged Tricyclic Benzopyrans: Enantioselective Catalytic Diels–Alder Reaction and a One-Pot Reduction/Acid-Catalyzed Stereoselective Cyclization. *Angew. Chem. Int. Ed.* **2014,** *53* (19), 4940-4944.
6. Goswami, P.; Das, B., Efficient organocatalyzed solvent-free selective synthesis of conjugated enones. *Tetrahedron Lett.* **2009,** *50* (8), 897-900.
7. Zhang, Y.; Sun, C.; Liang, J.; Shang, Z., Catalysis by L-Lysine: A Green Method for the Condensation of Aromatic Aldehydes with Acidic Methylene Compounds in Water at Room Temperature. *Chin. J. Chem* . **2010,** *28* (11), 2255-2259.

8. Seckler, D.; Dea, C. M.; Rios, E. A. M.; de Godoi, M.; Rampon, D. d. S.; D’Oca, M. G. M.; D'Oca, C. D. R. M., Rice straw ash extract/glycerol: an efficient sustainable approach for Knoevenagel condensation. *New J. Chem.* **2022,** *46* (10), 4570-4578.
9. Wu, J.; Mou, C.; Chi, Y. R., Construction of Multi-Substituted Benzenes via NHC-Catalyzed Reactions of Carboxylic Esters. *Chin. J. Chem .* **2018,** *36* (4), 333-337.
10. Hu, Y.; He, Y.-H.; Guan, Z., A simple method for the preparation of functionalized trisubstituted alkenes and α,β,γ,δ-unsaturated carbonyl compounds by using natural amino acid l-tryptophan. *Catal. Commun.* **2010,** *11* (7), 656-659.
11. Bernasconi, C. F.; Stronach, M. W., Kinetics of amine addition to benzylidene-1,3-indandione and other vinylic .beta.-diketones. Effect of cyclic structure and steric strain on intrinsic rate constants. *Journal of the American Chemical Society* **1991,** *113* (6), 2222-2227.
12. Zhu, T.; Zheng, P.; Mou, C.; Yang, S.; Song, B.-A.; Chi, Y. R., Benzene construction via organocatalytic formal [3+3] cycloaddition reaction. *Nature Communications* **2014,** *5* (1), 5027.
13. Shahzad, S. A.; Venin, C.; Wirth, T., Diselenide- and Disulfide-Mediated Synthesis of Isocoumarins. *Eur. J. Org. Chem.* **2010,** *2010* (18), 3465-3472.

^{1}H NMR (400 MHz, $CDCl_3$) of compound (**3a**)

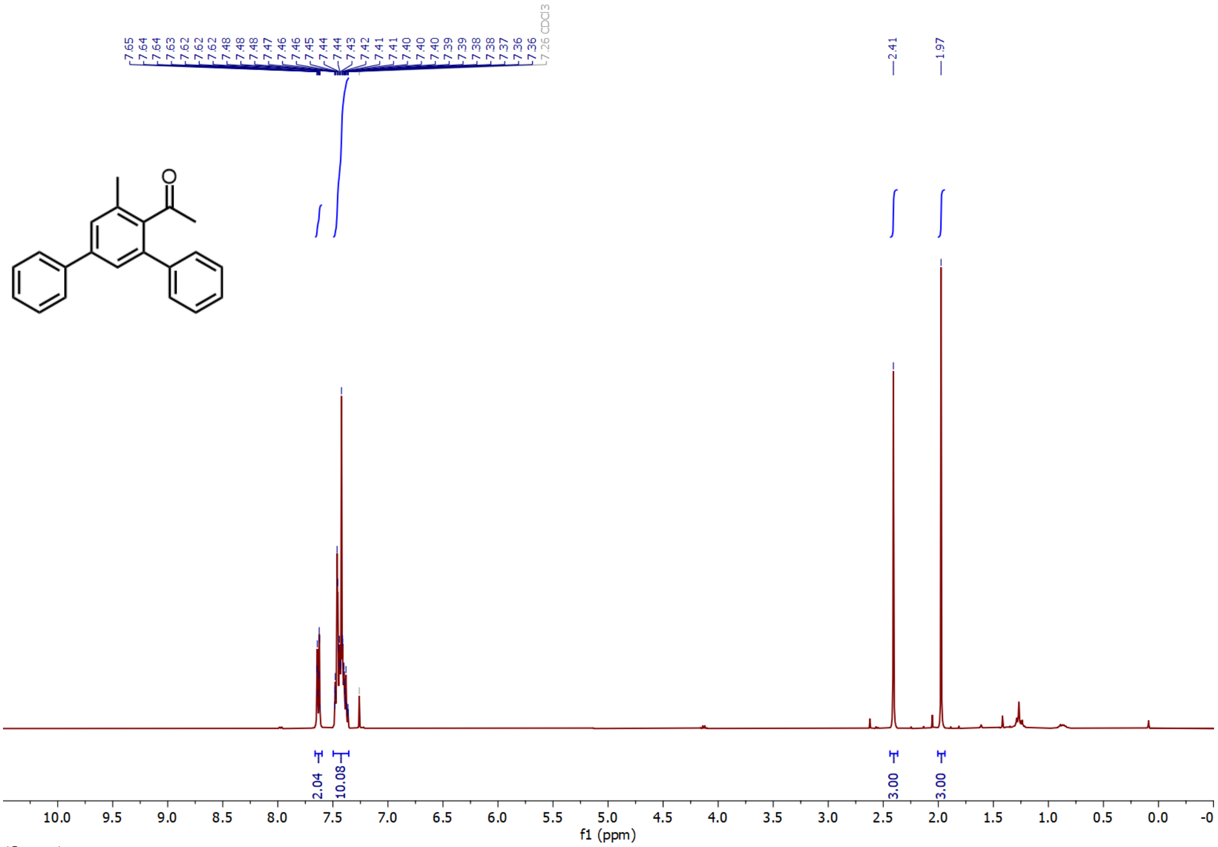

^{13}C {^{1}H} NMR (101 MHz, $CDCl_3$) of compound (**3a**)

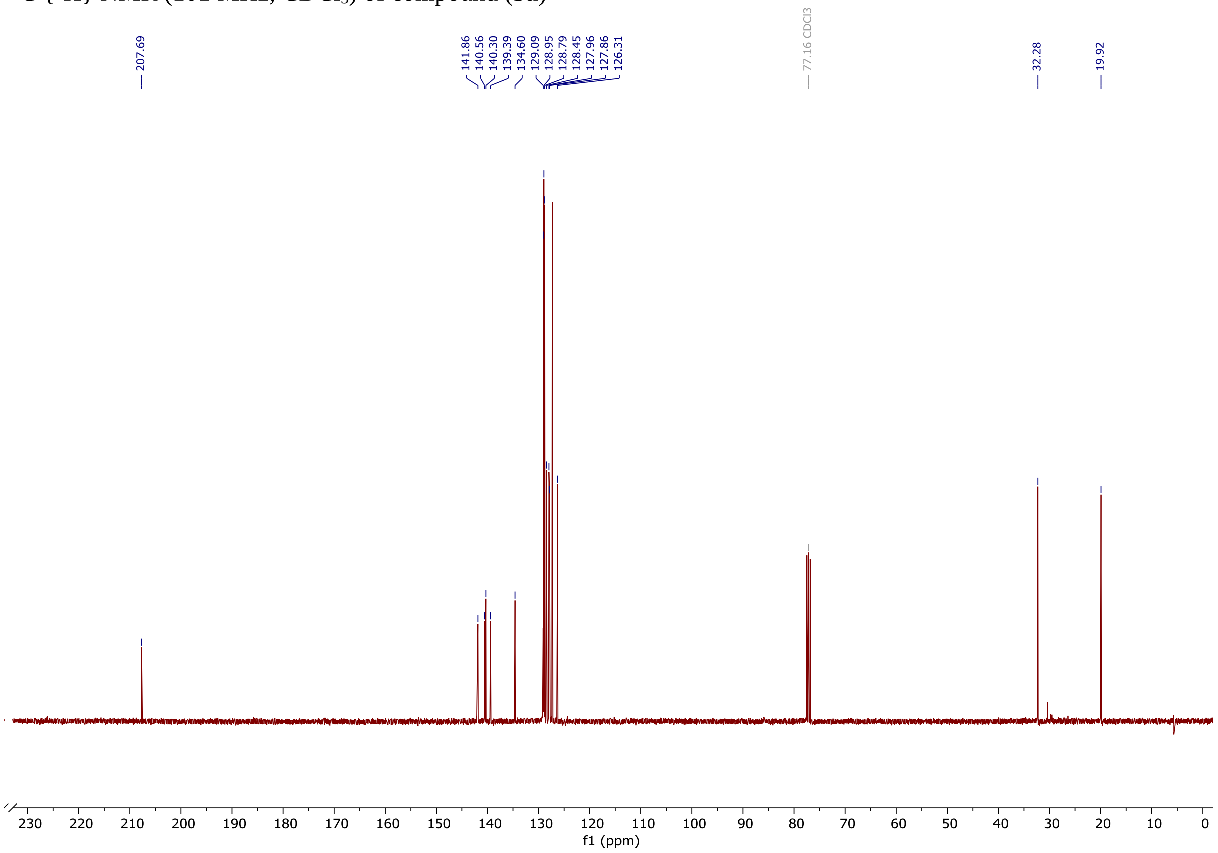

^{1}H NMR (400 MHz, $CDCl_3$) of compound (**3b**)

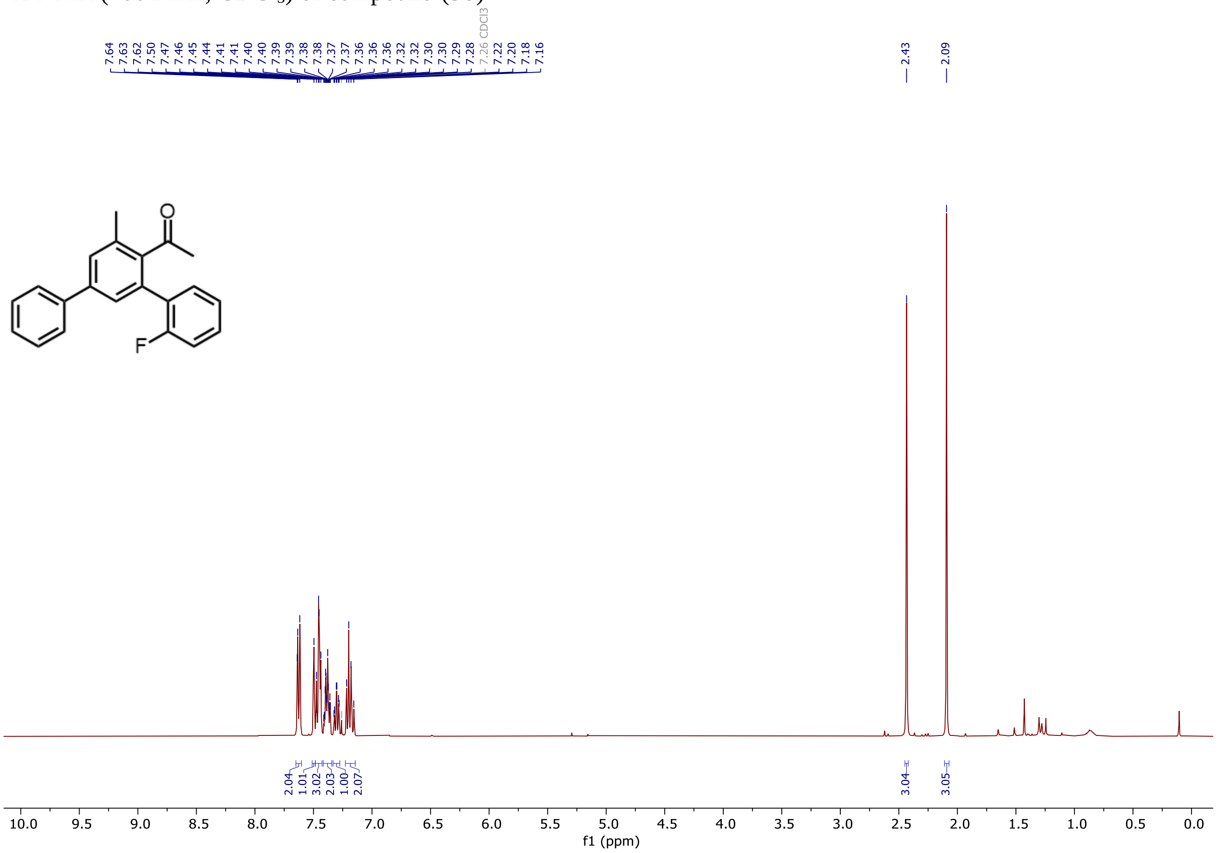

^{13}C {^{1}H} NMR (101 MHz, $CDCl_3$) of compound (**3e**)

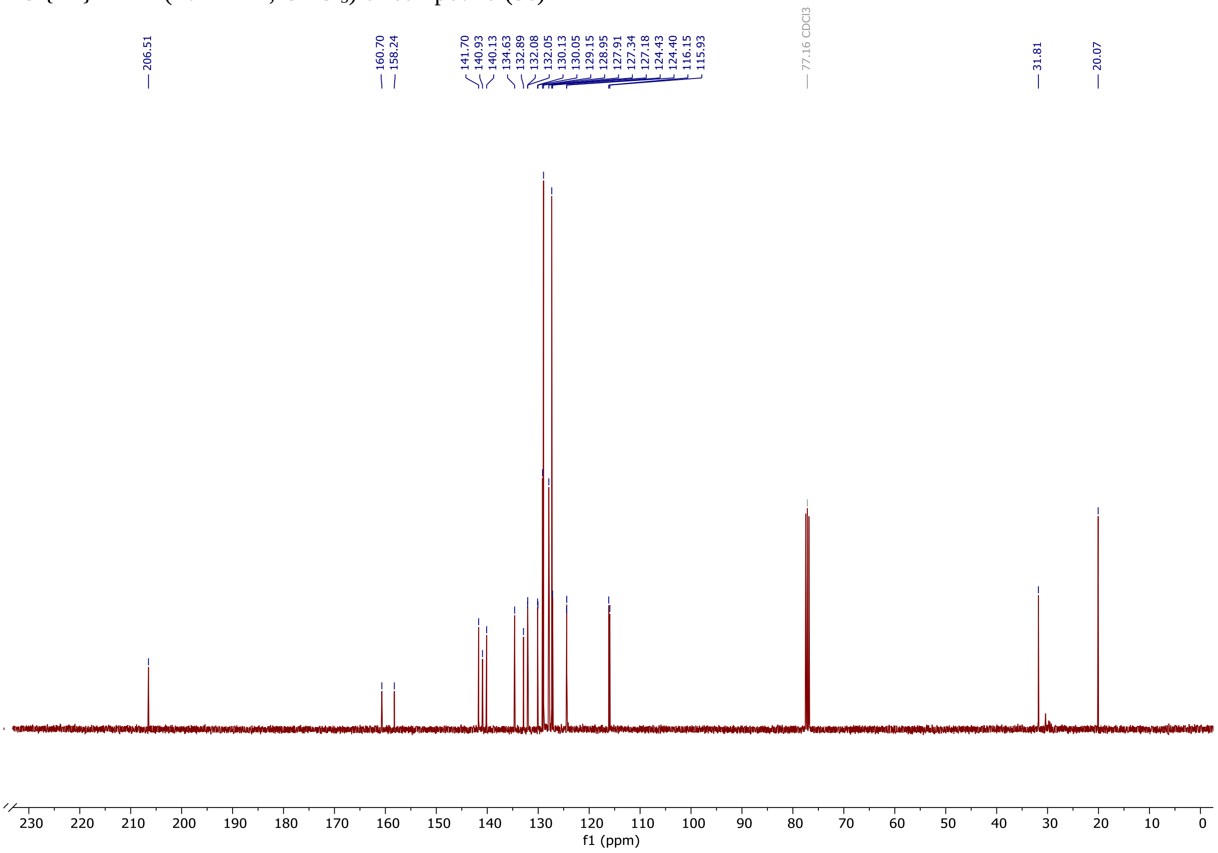

^{1}H NMR (400 MHz, $CDCl_3$) of compound (**3c**)

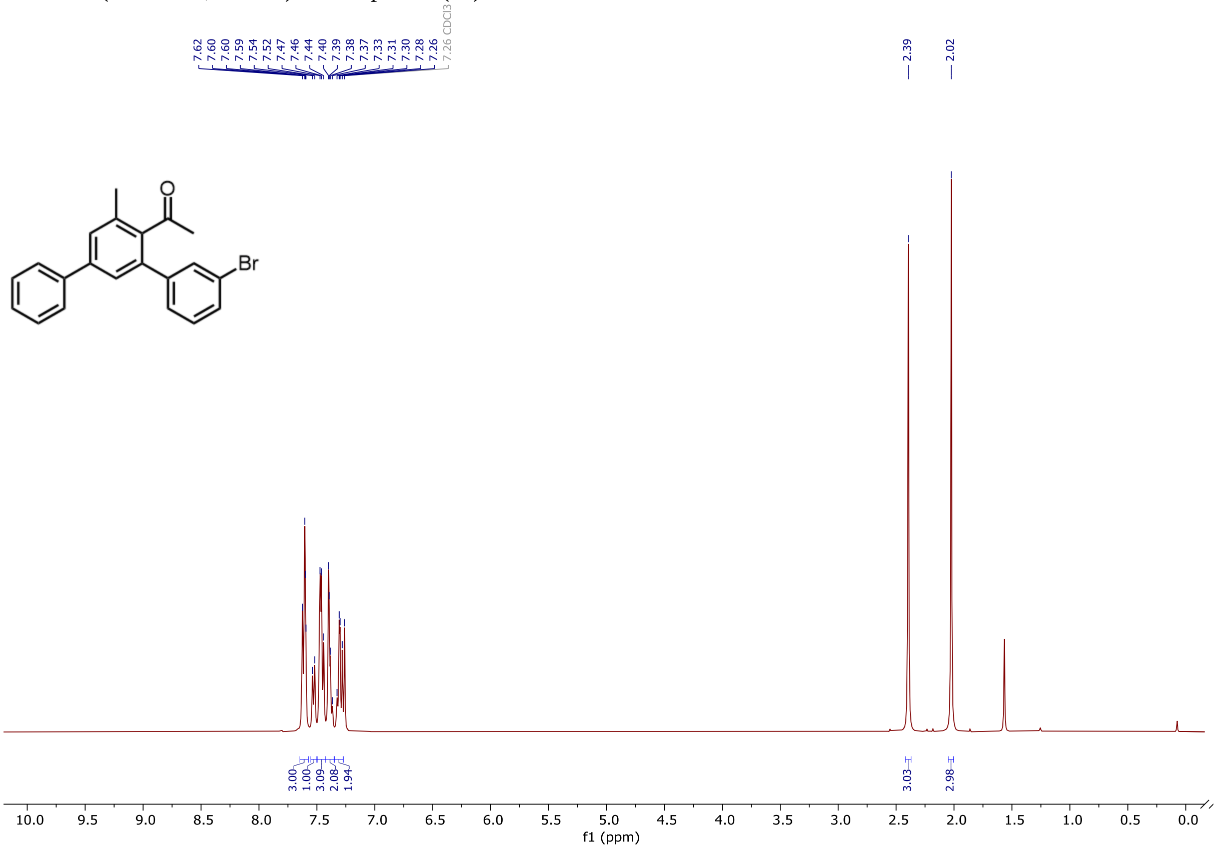

^{13}C {^{1}H} NMR (101 MHz, $CDCl_3$) of compound (**3c**)

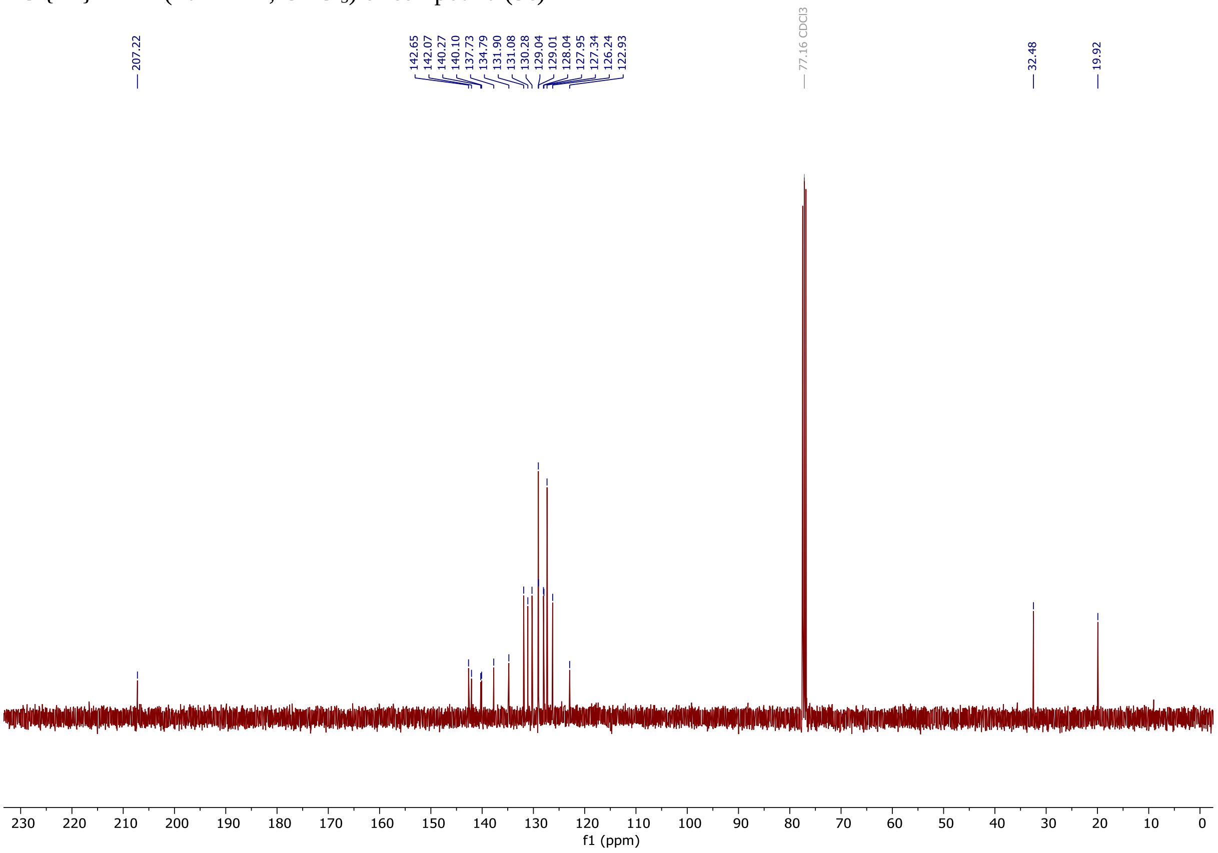

^{1}H NMR (400 MHz, $CDCl_3$) of compound (**3d**)

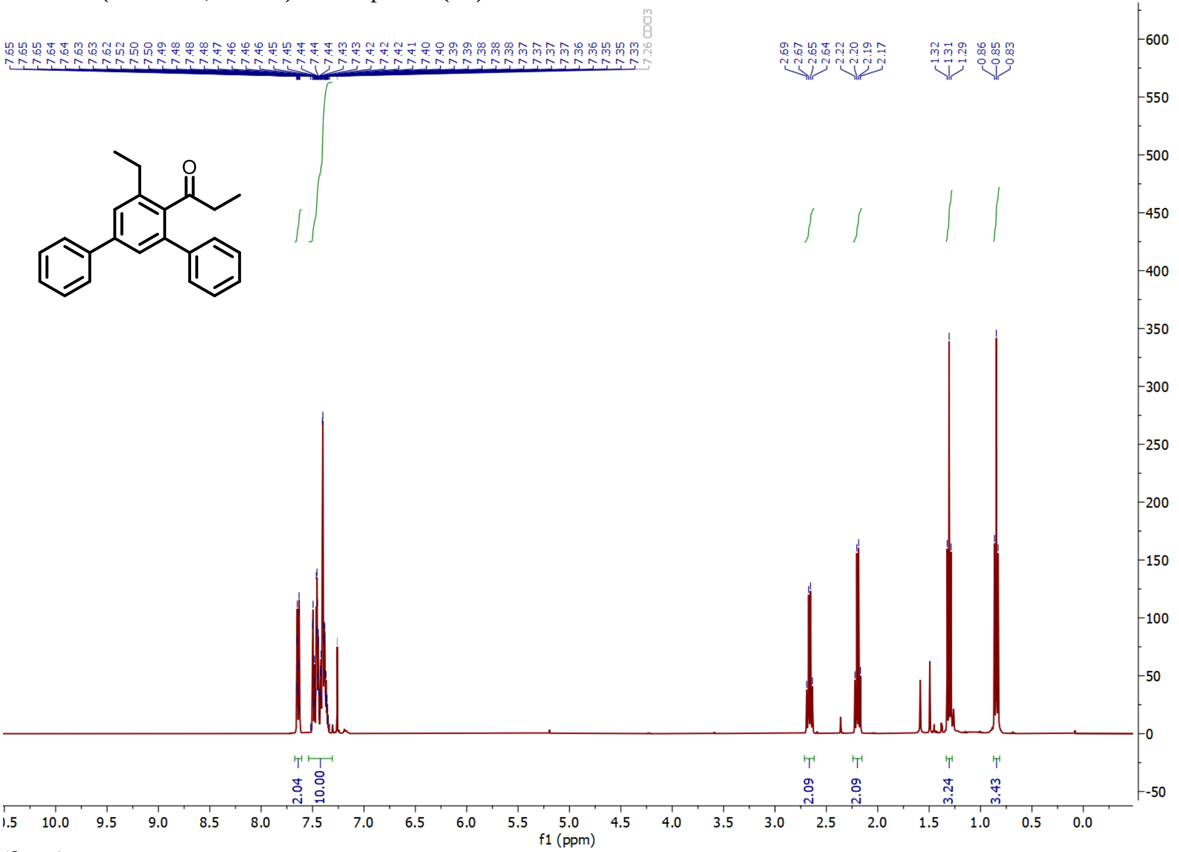

^{13}C {^{1}H} NMR (101 MHz, $CDCl_3$) of compound (**3d**)

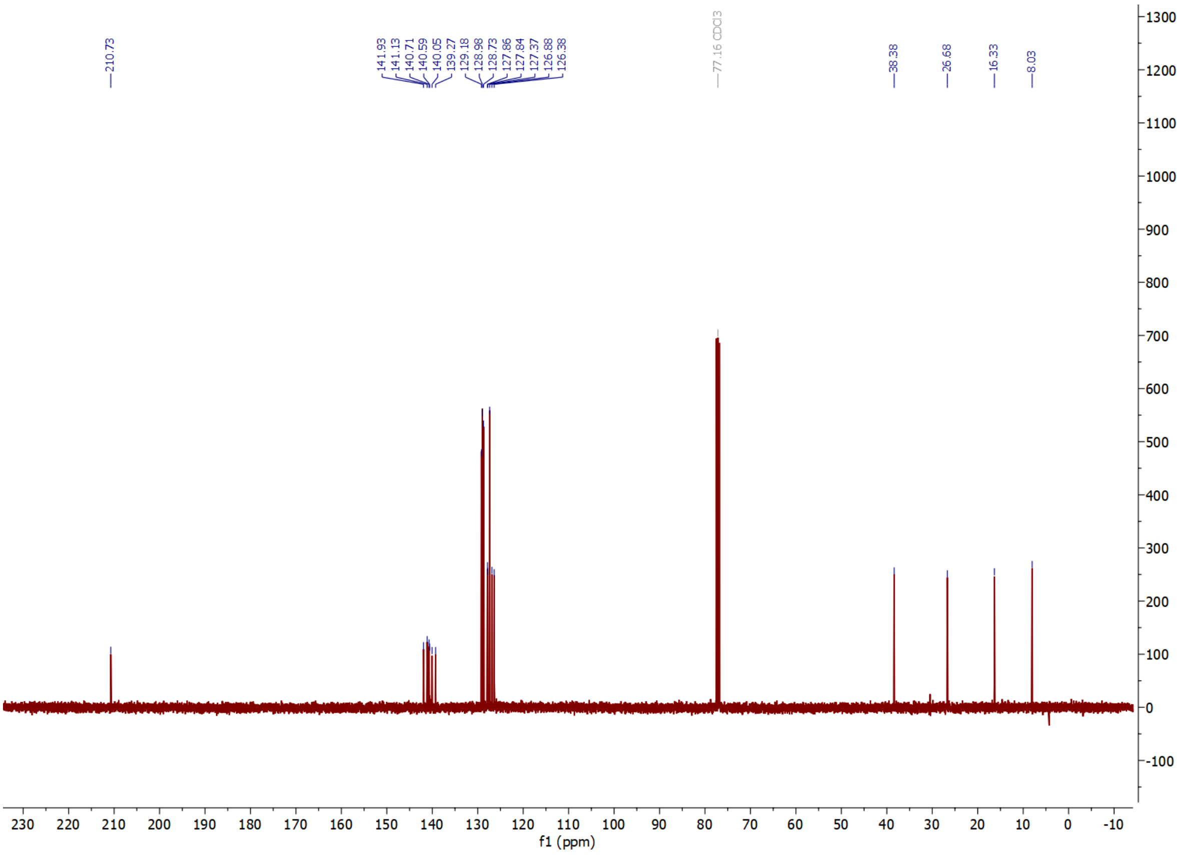

^{1}H NMR (400 MHz, $CDCl_3$) of compound (**3e**)

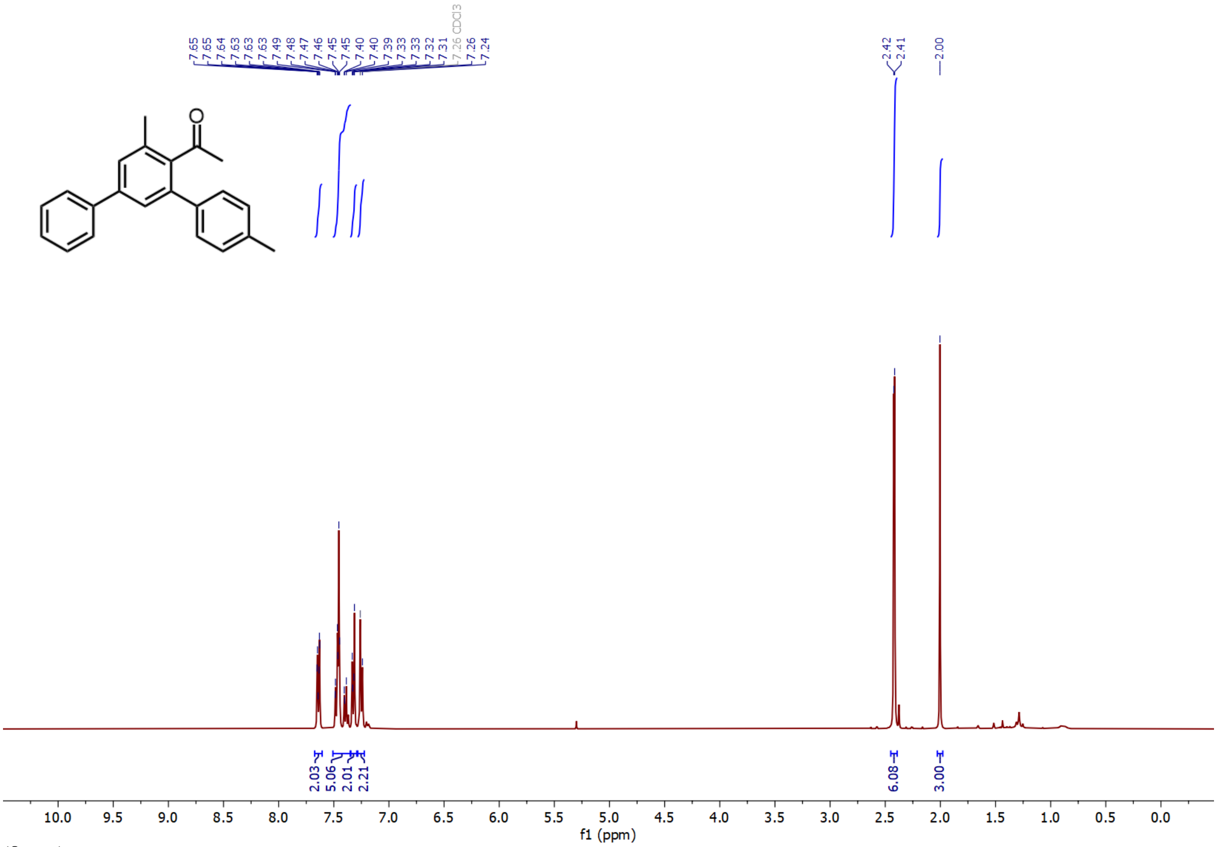

^{13}C {^{1}H} NMR (101 MHz, $CDCl_3$) of compound (**3e**)

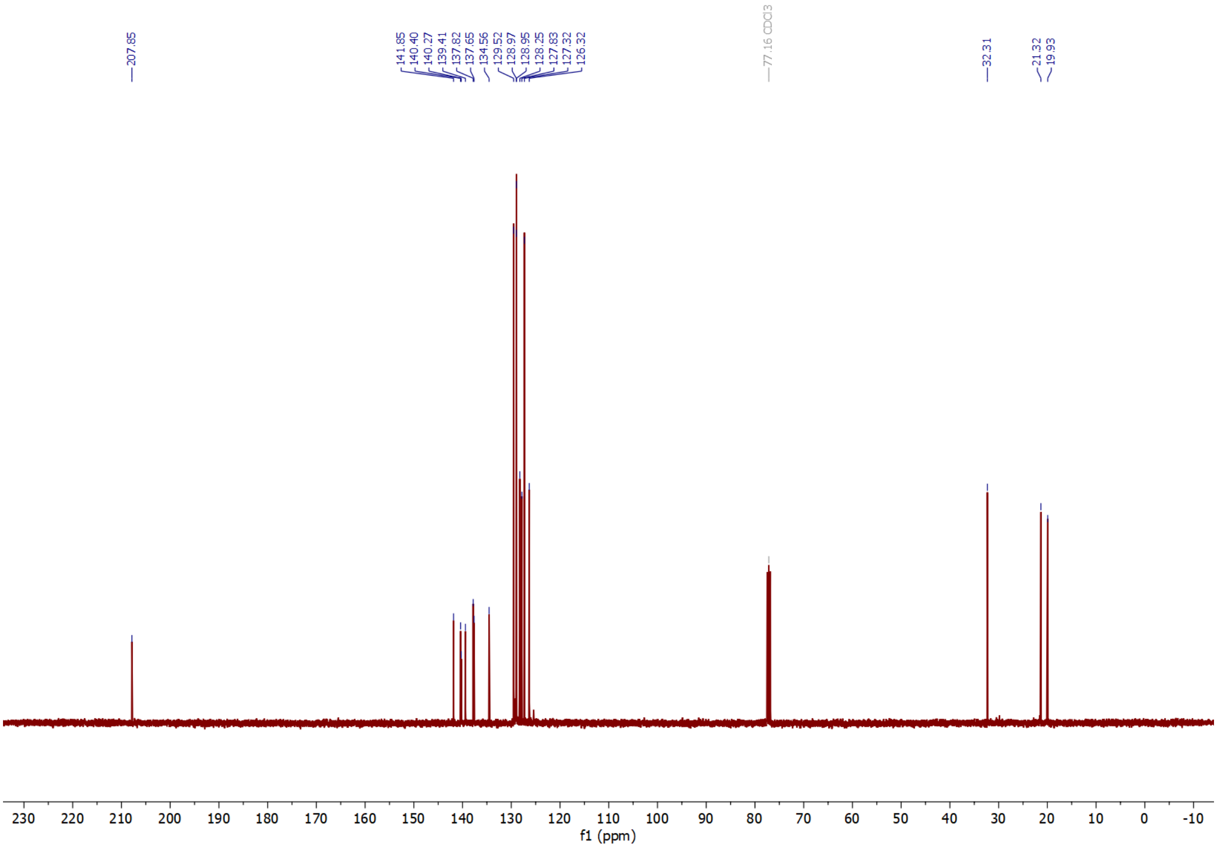

^{1}H NMR (400 MHz, $CDCl_3$) of compound (**3f**)

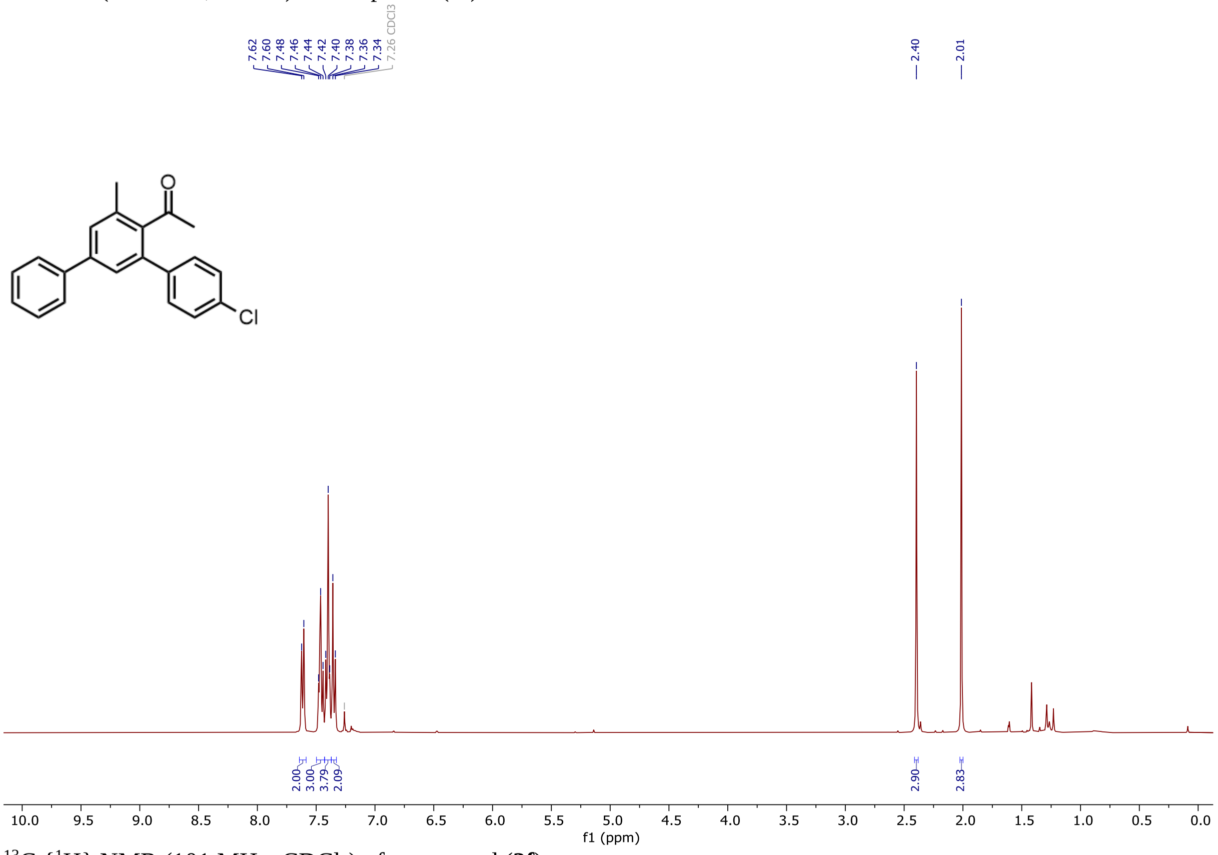

^{13}C {^{1}H} NMR (101 MHz, $CDCl_3$) of compound (**3f**)

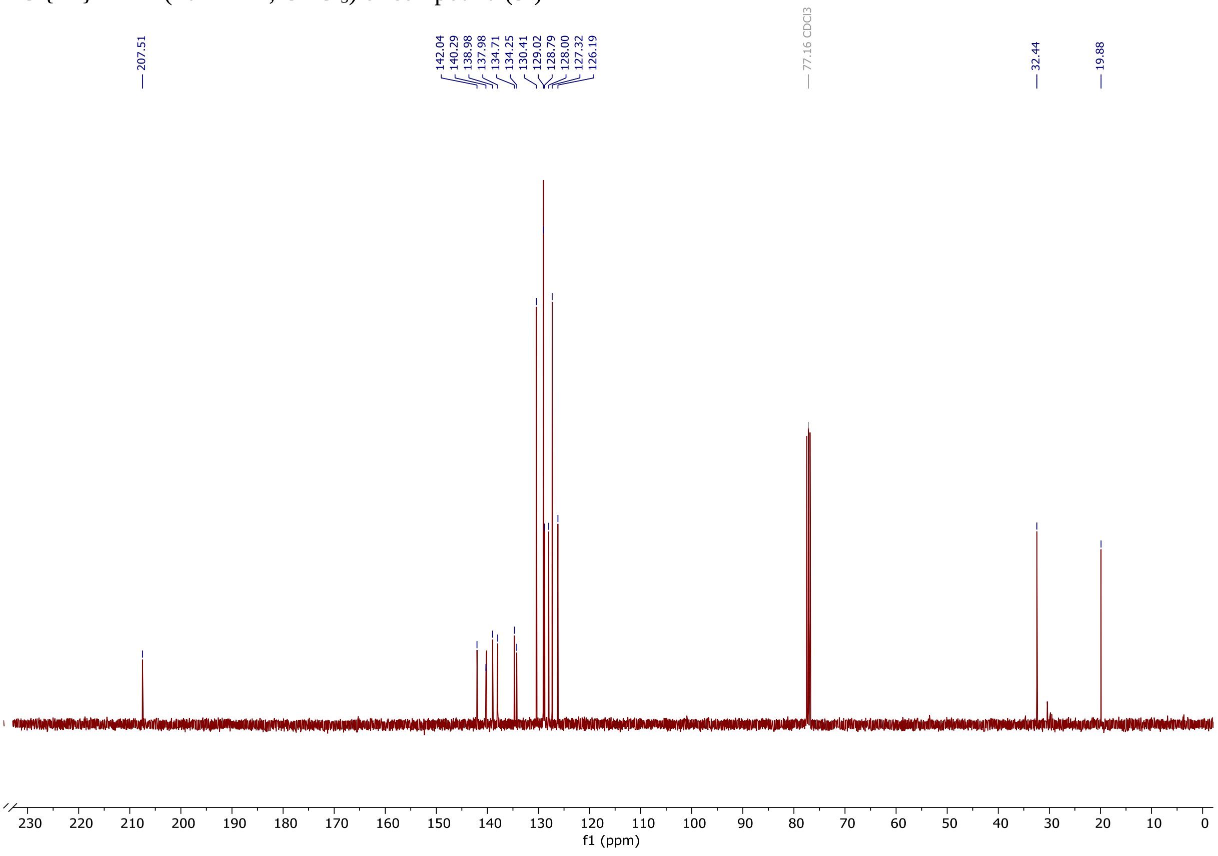

^{1}H NMR (400 MHz, $CDCl_3$) of compound (**3g**)

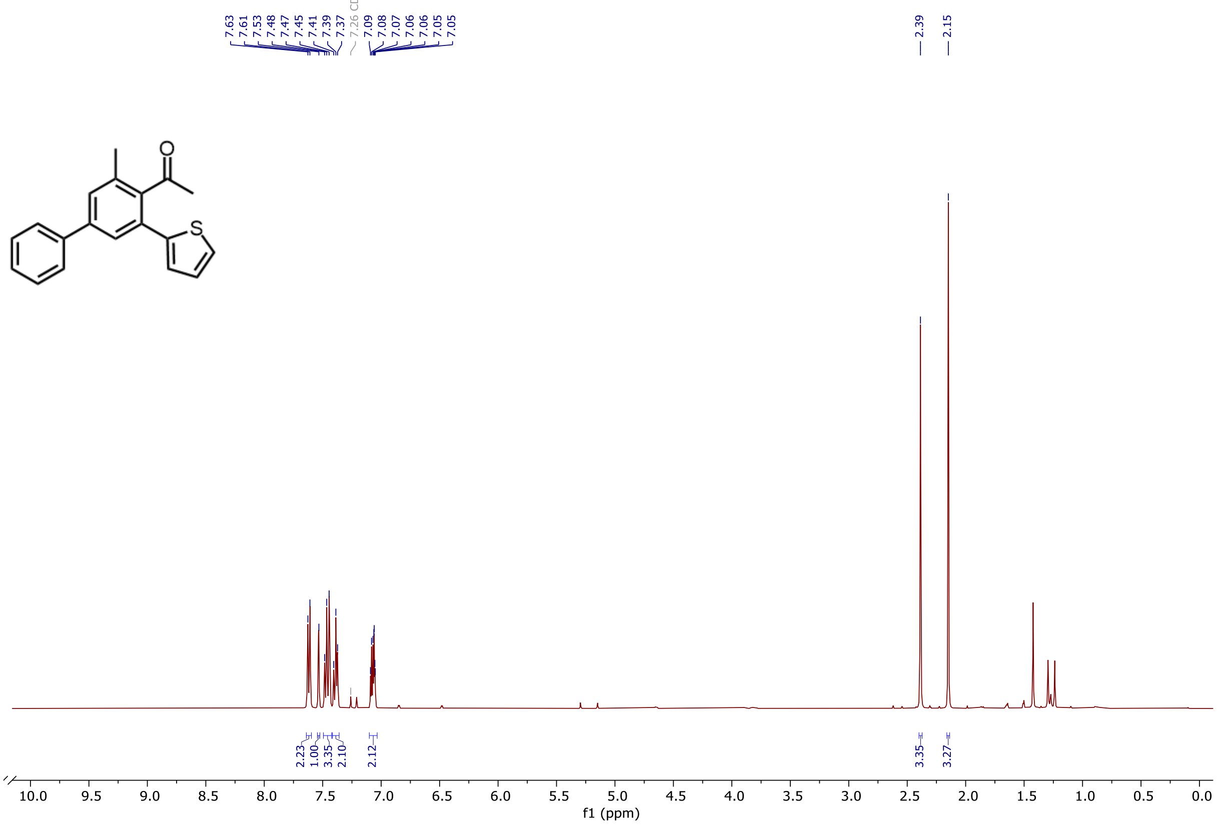

^{13}C {^{1}H} NMR (101 MHz, $CDCl_3$) of compound (**3g**)

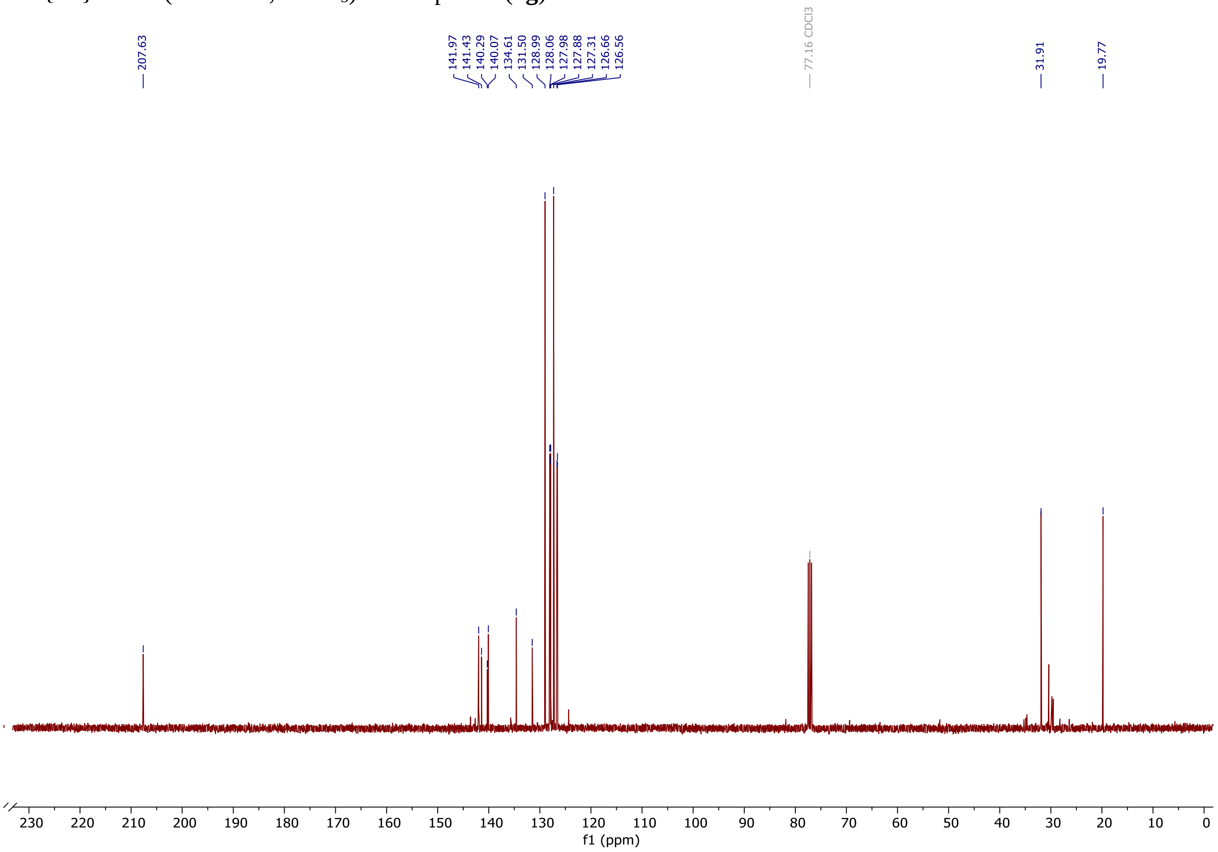

^{1}H NMR (400 MHz, $CDCl_3$) of compound (**3h**)

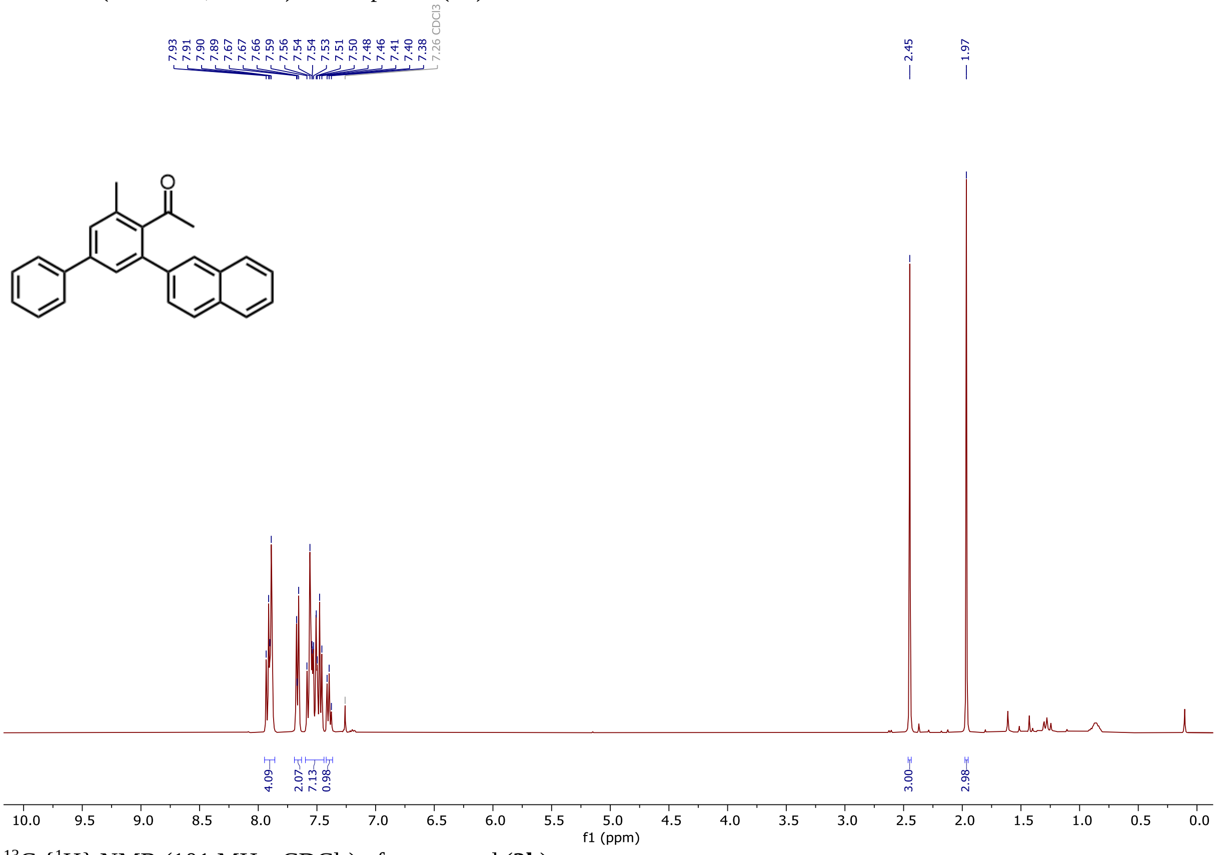

^{13}C {^{1}H} NMR (101 MHz, $CDCl_3$) of compound (**3h**)

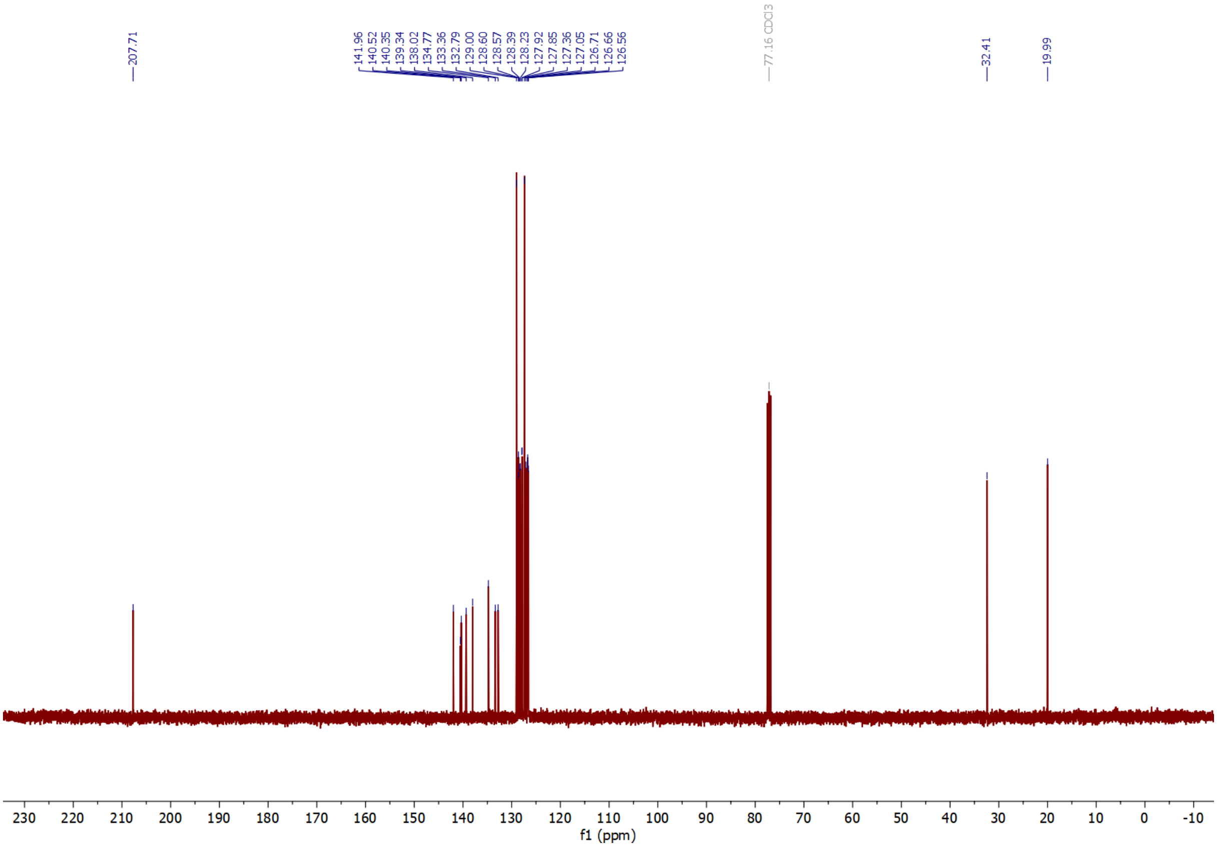

^{1}H NMR (400 MHz, $CDCl_3$) of compound (**3i**)

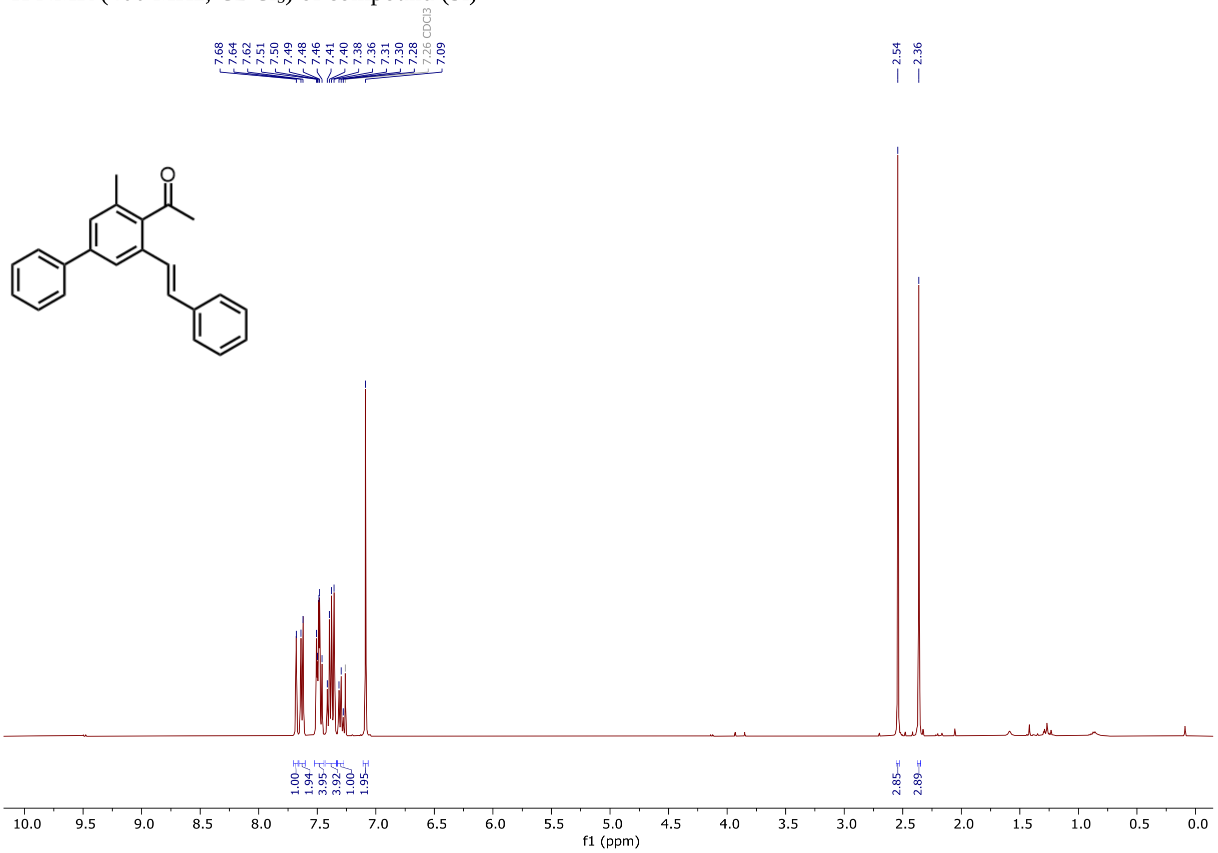

^{13}C {^{1}H} NMR (101 MHz, $CDCl_3$) of compound (**3i**)

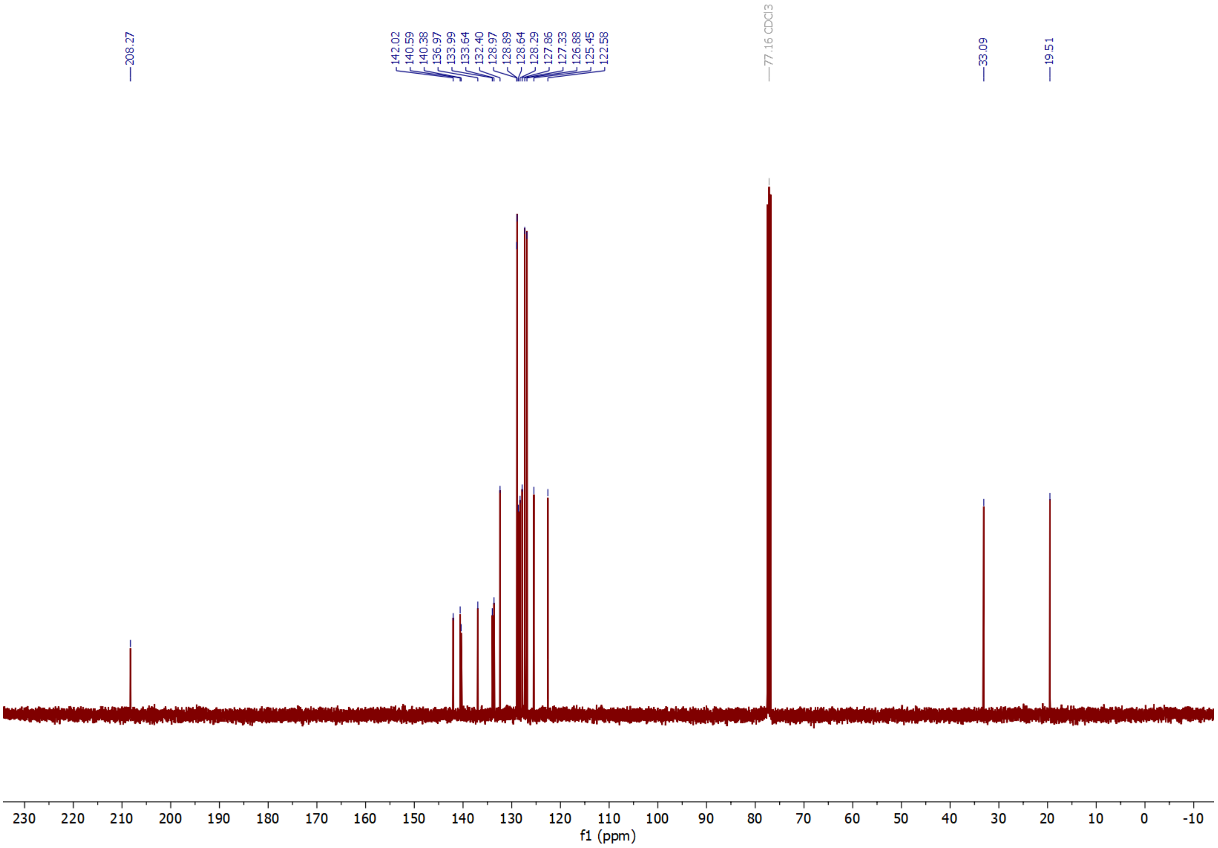

^{1}H NMR (400 MHz, $CDCl_3$) of compound (**3j**)

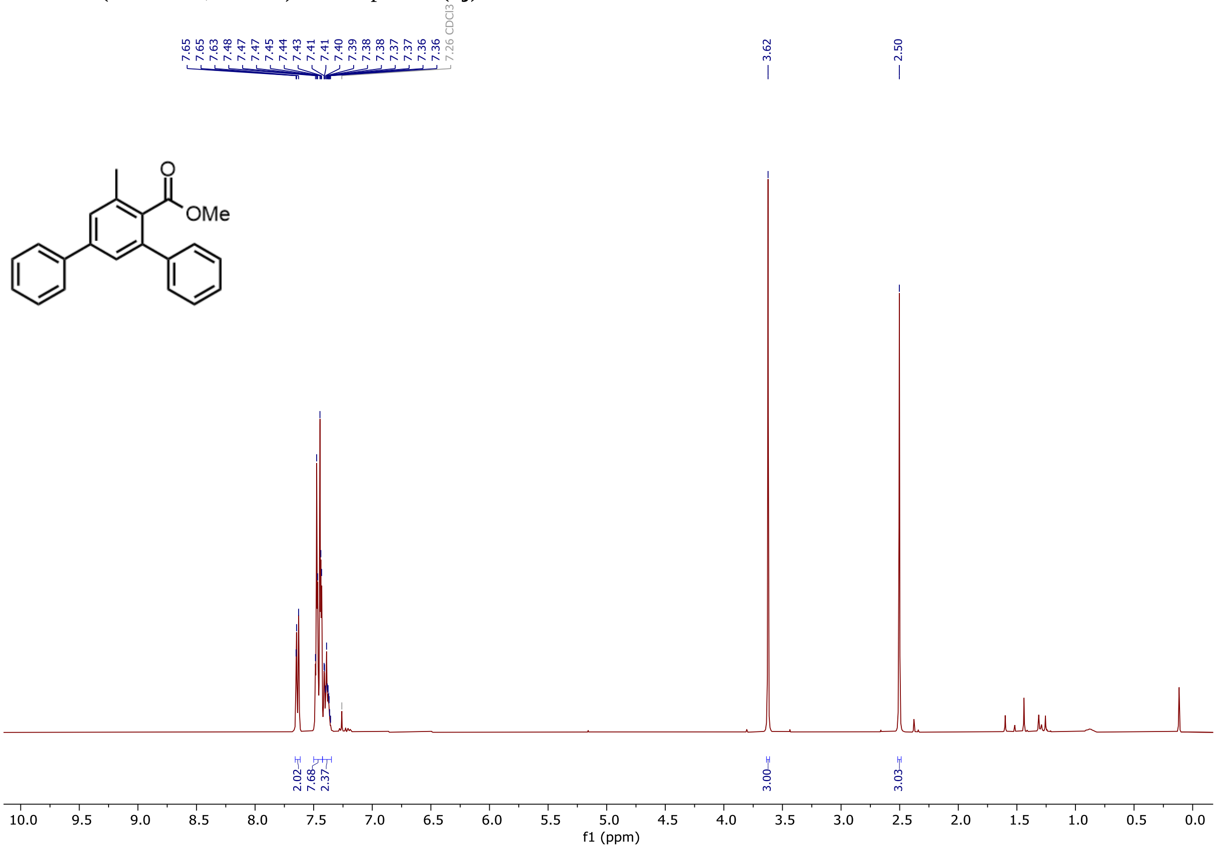

^{13}C {^{1}H} NMR (101 MHz, $CDCl_3$) of compound (**3j**)

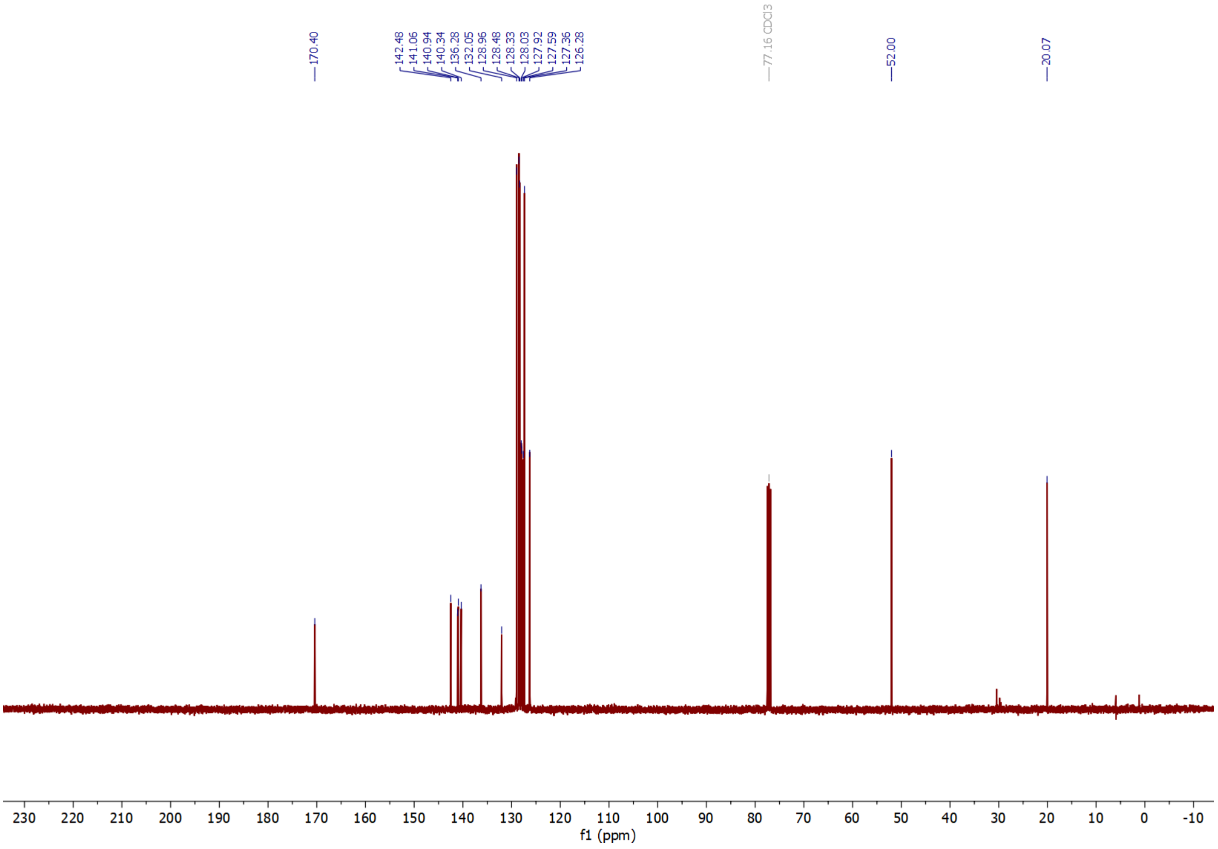

^{1}H NMR (400 MHz, $CDCl_3$) of compound (**3k**)

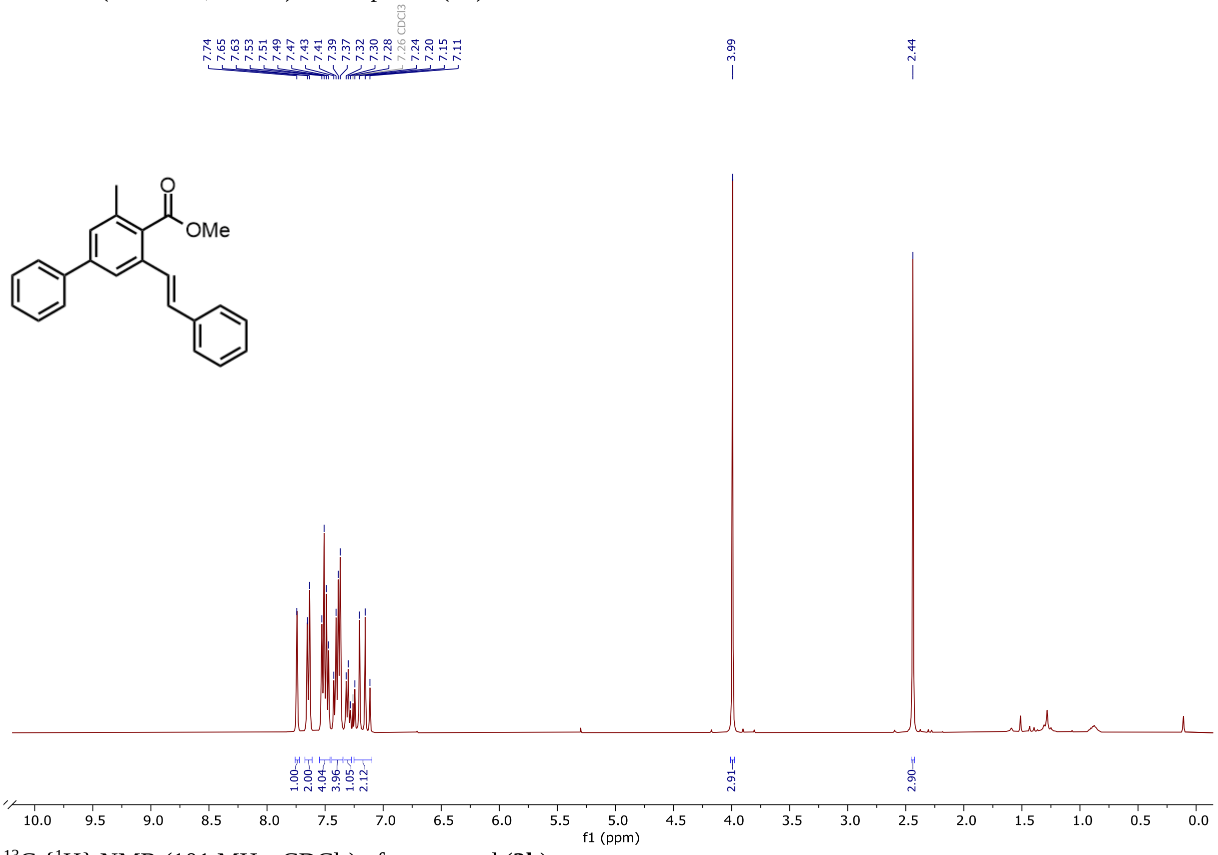

^{13}C {^{1}H} NMR (101 MHz, $CDCl_3$) of compound (**3k**)

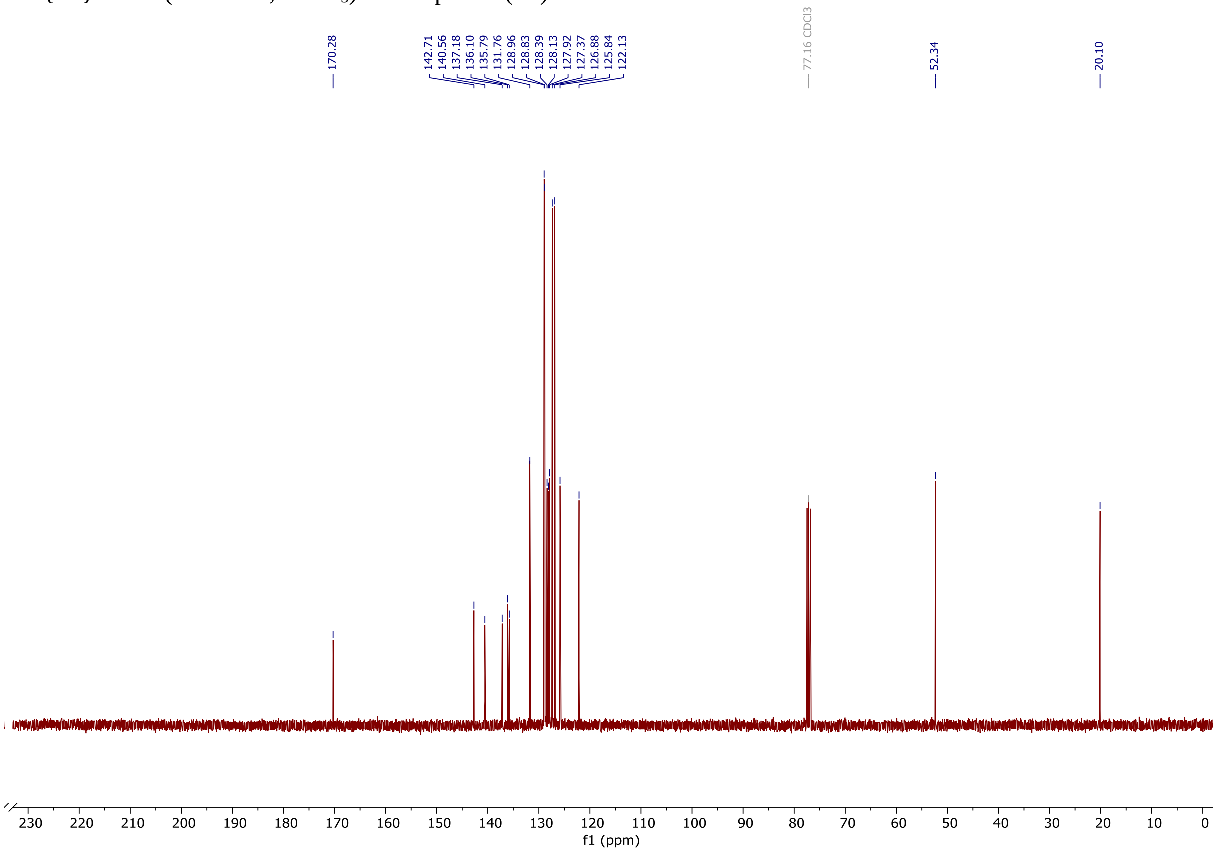

^{1}H NMR (400 MHz, $CDCl_3$) of compound (**3l**)

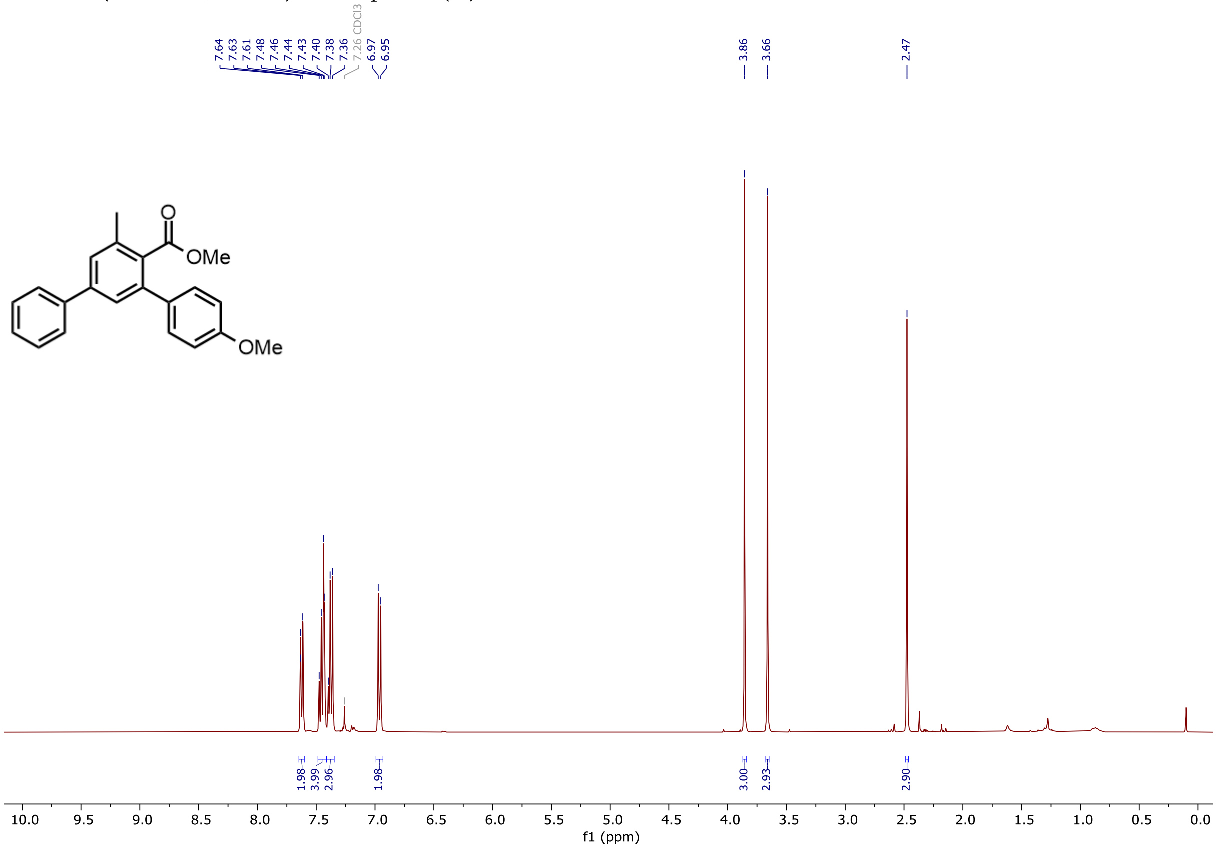

^{13}C {^{1}H} NMR (101 MHz, $CDCl_3$) of compound (**3l**)

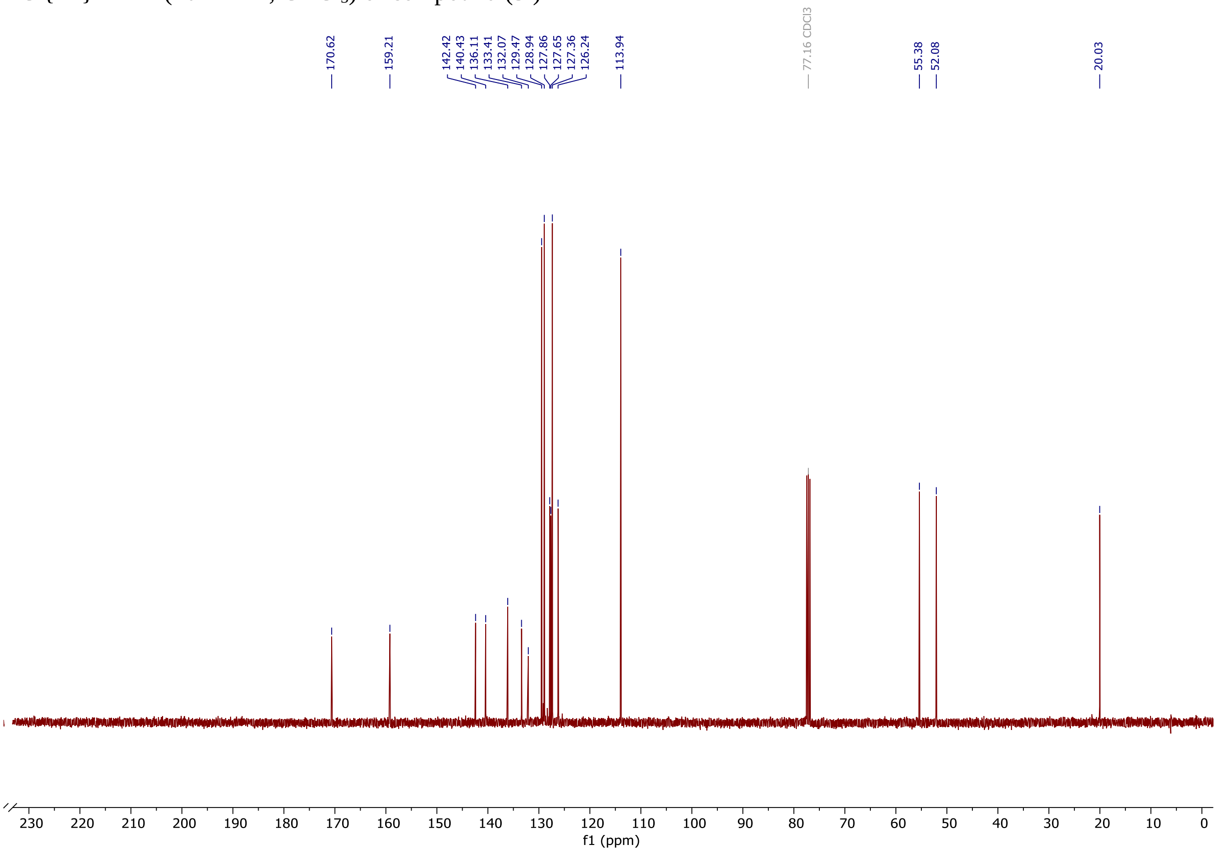

^{1}H NMR (400 MHz, $CDCl_3$) of compound (**3m**)

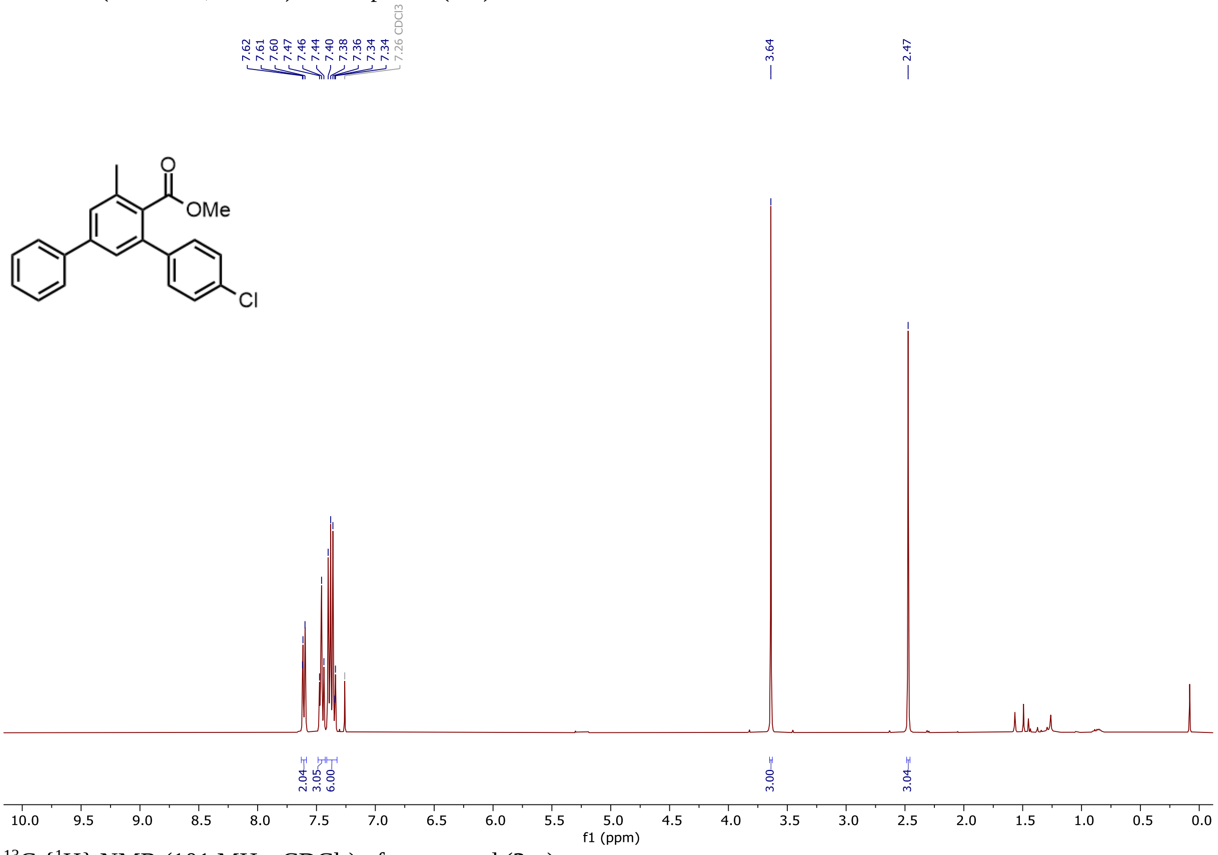

^{13}C {^{1}H} NMR (101 MHz, $CDCl_3$) of compound (**3m**)

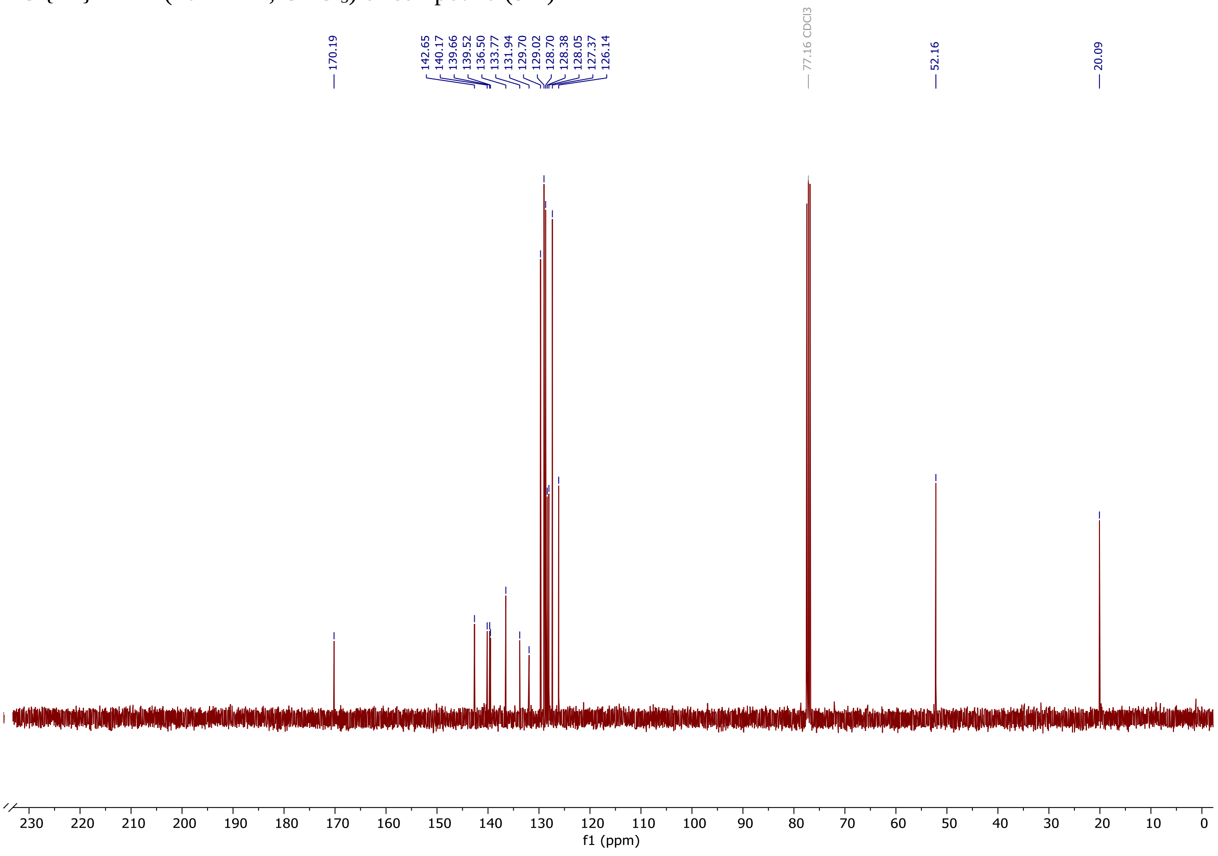

^{1}H NMR (400 MHz, $CDCl_3$) of compound (**3n**)

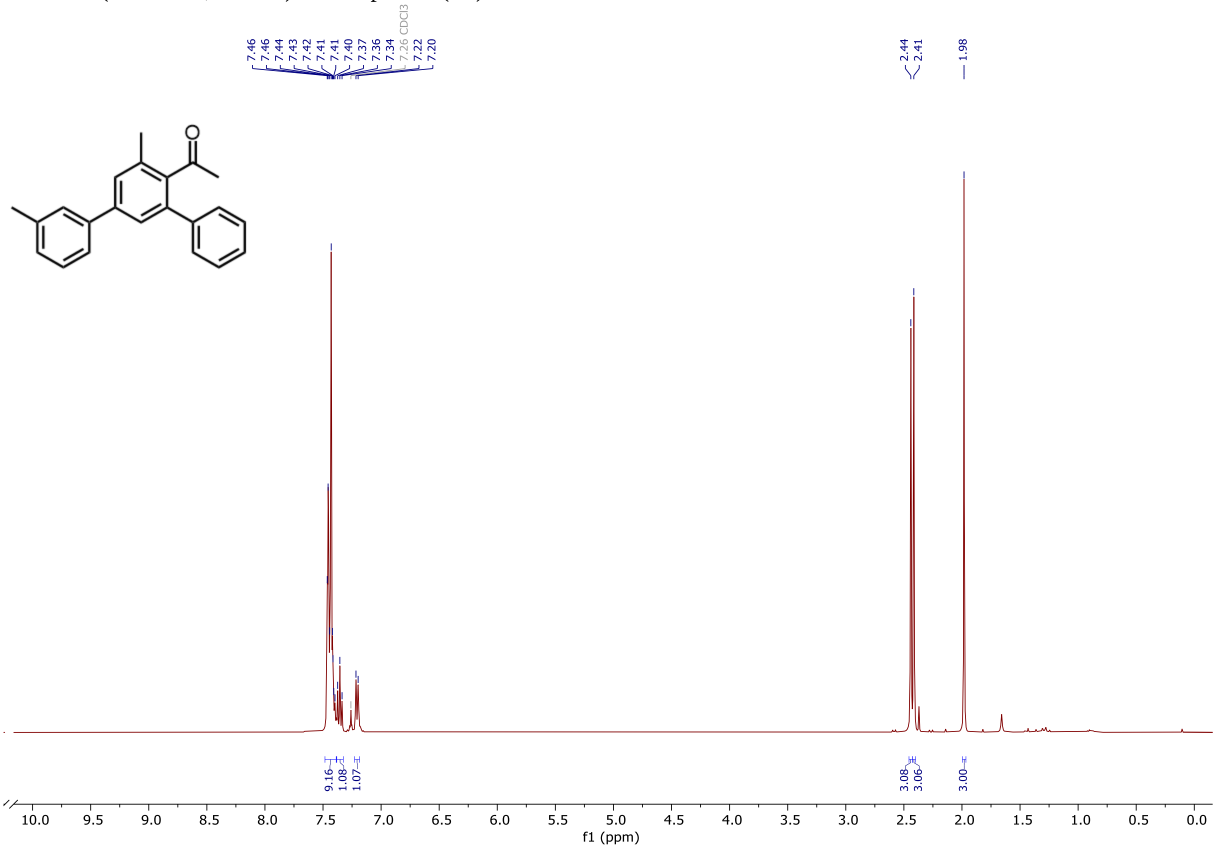

^{13}C {^{1}H} NMR (101 MHz, $CDCl_3$) of compound (**3n**)

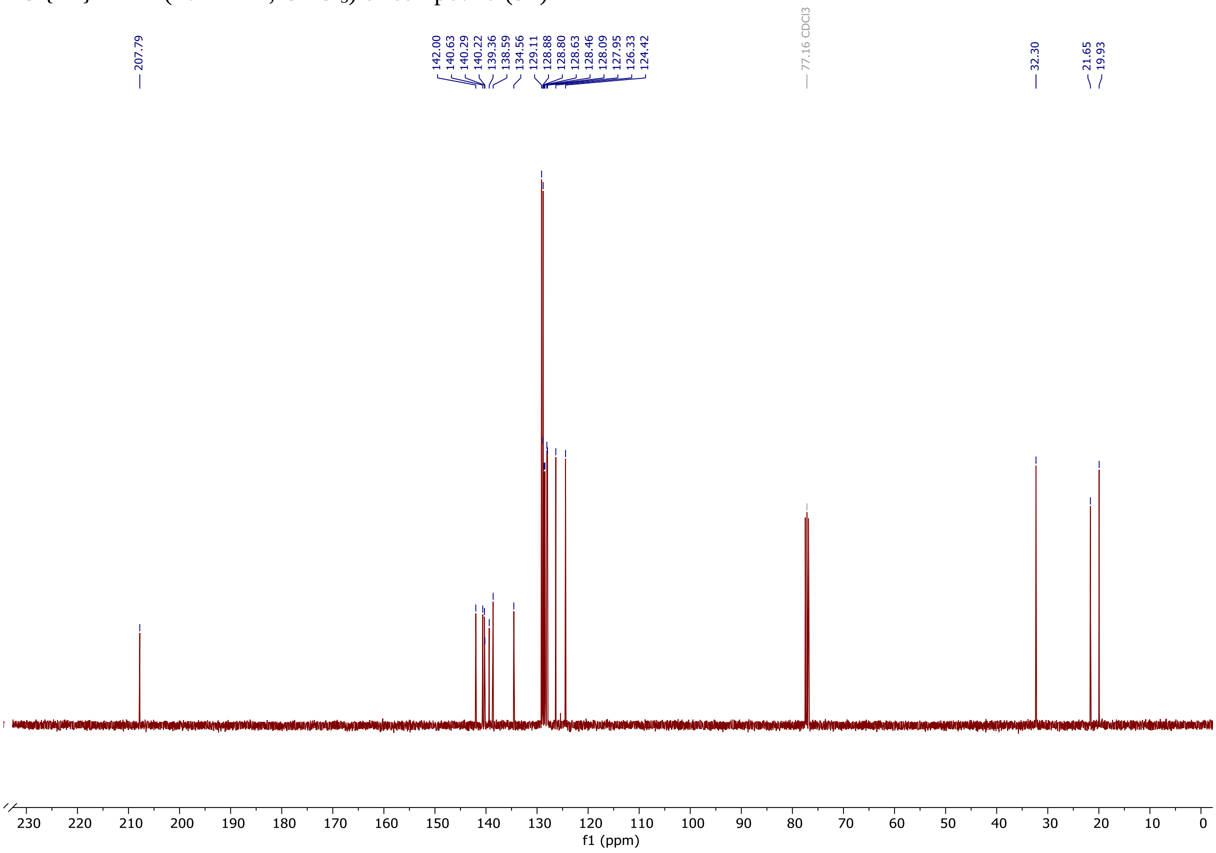

^{1}H NMR (400 MHz, $CDCl_3$) of compound (**3o**)

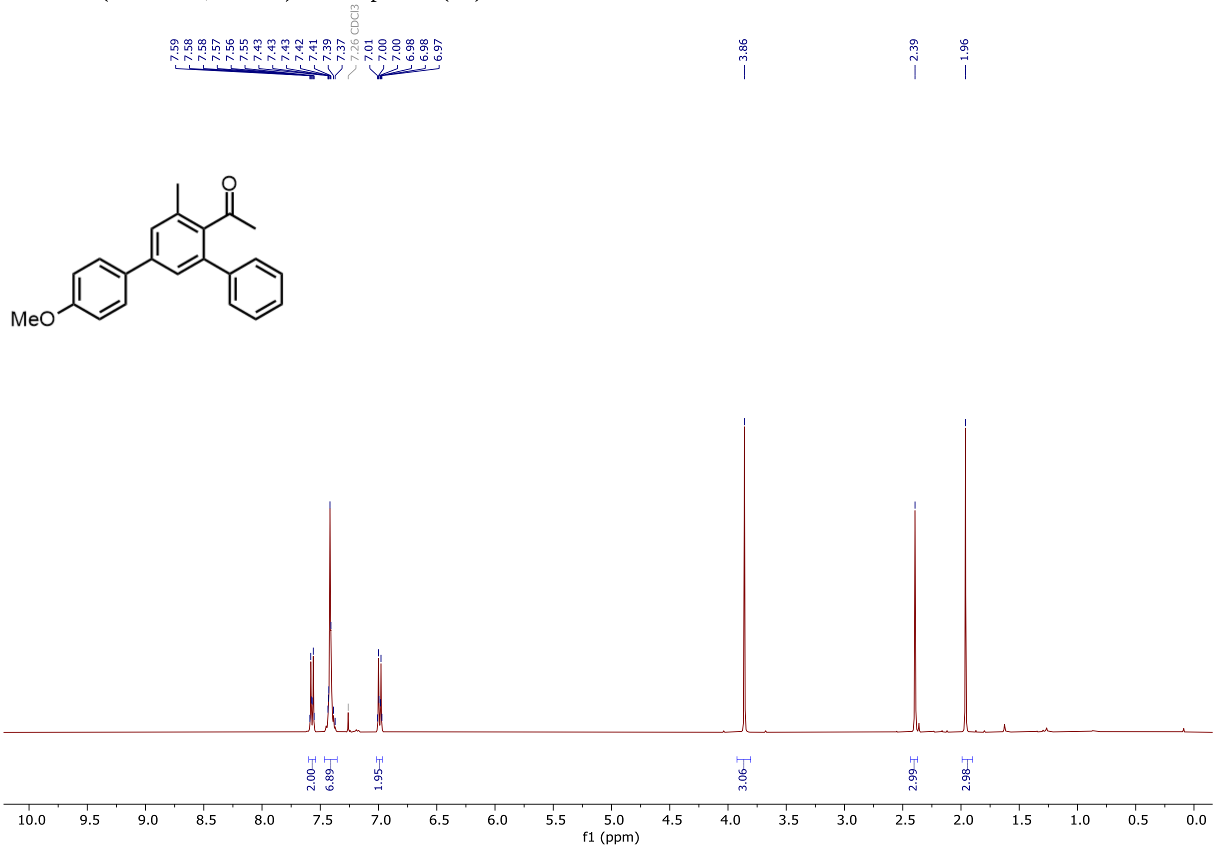

^{13}C {^{1}H} NMR (101 MHz, $CDCl_3$) of compound (**3o**)

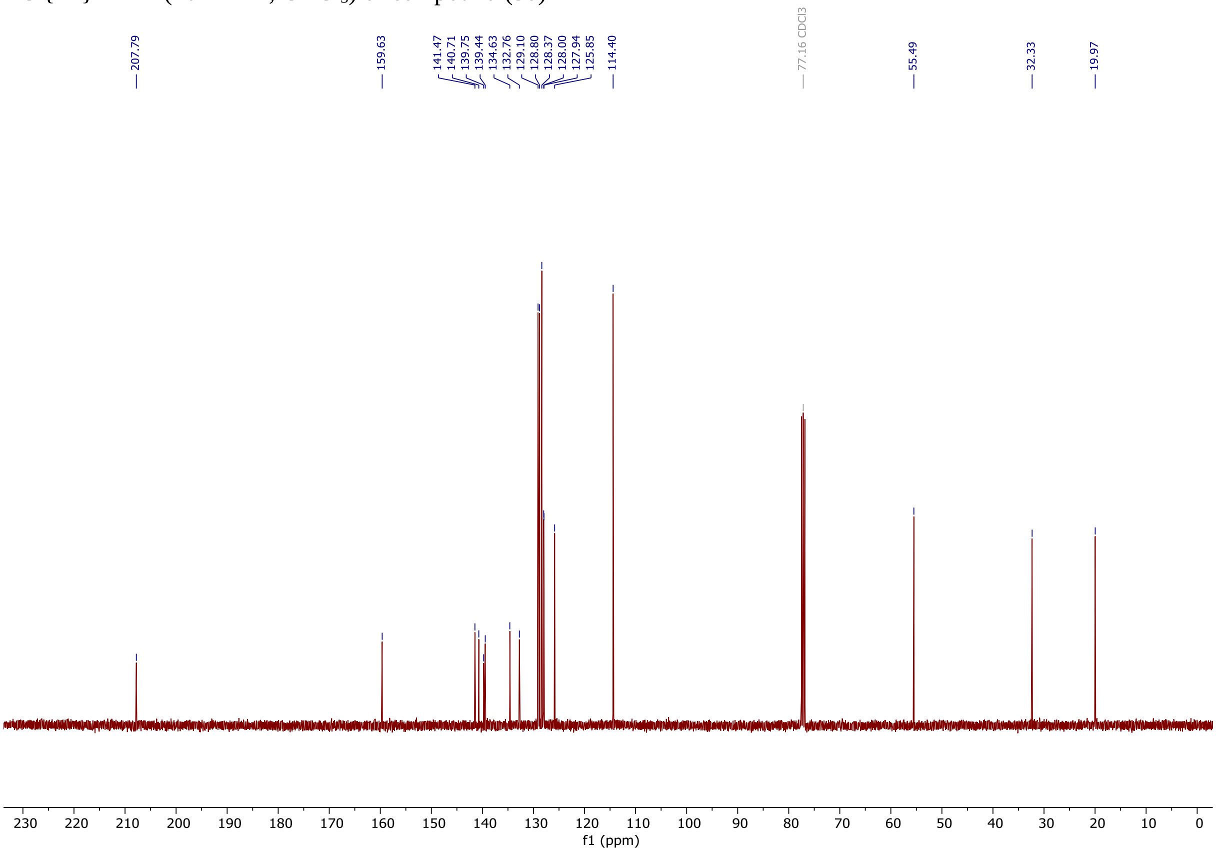

^{1}H NMR (400 MHz, $CDCl_3$) of compound (**3p**)

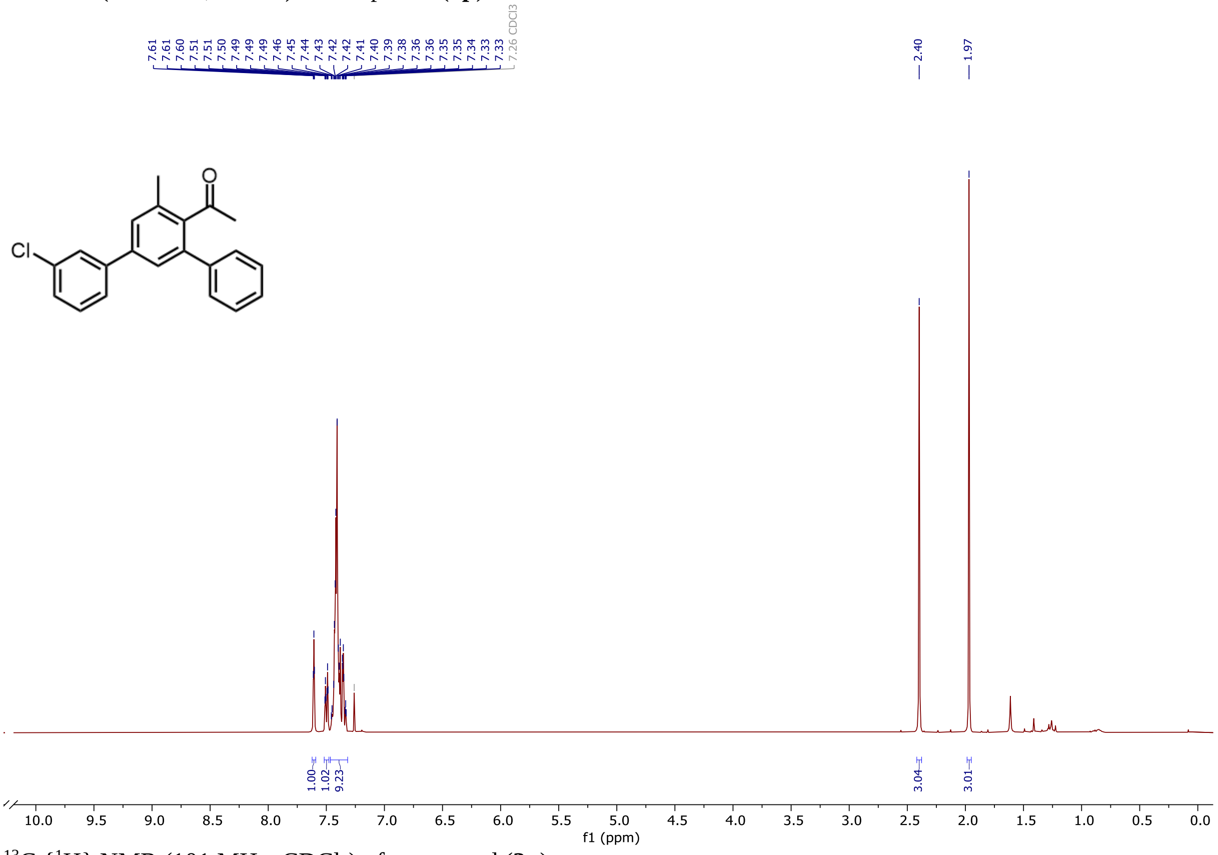

^{13}C {^{1}H} NMR (101 MHz, $CDCl_3$) of compound (**3p**)

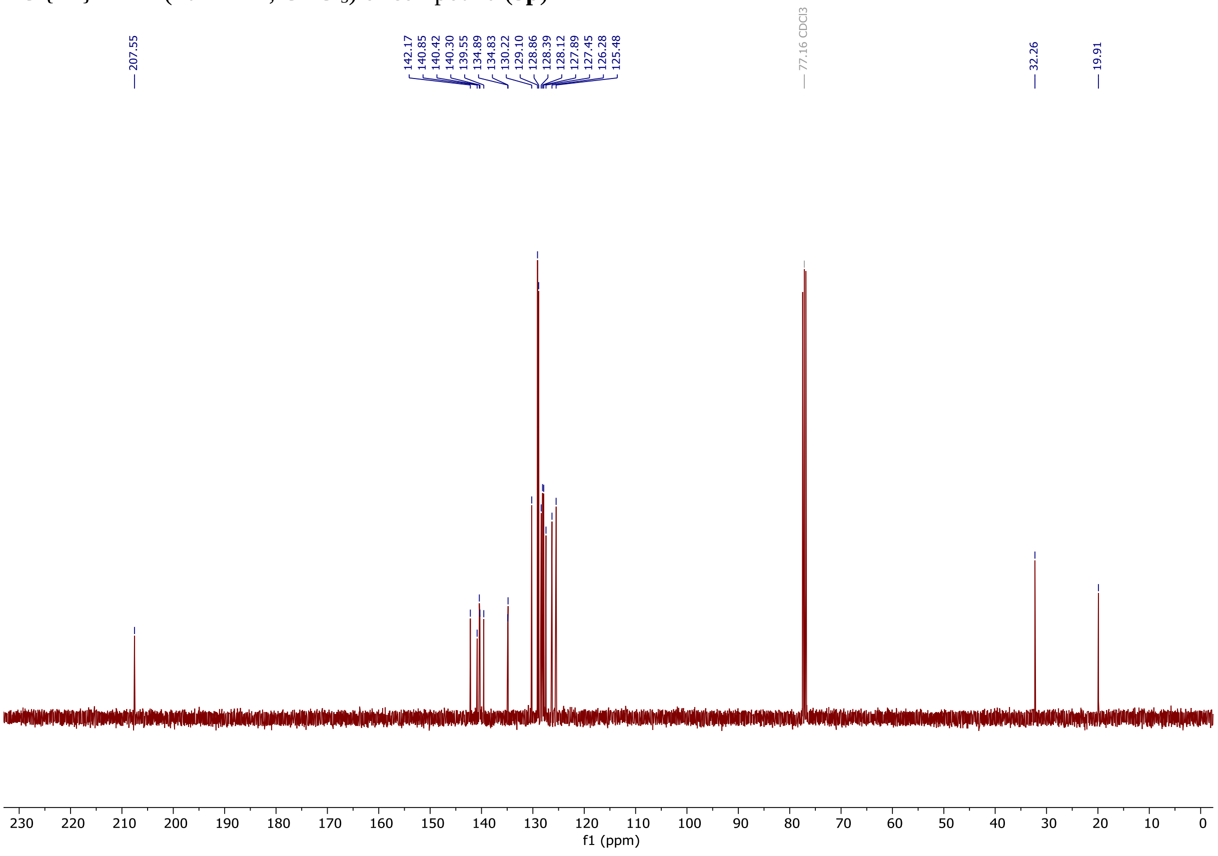

^{1}H NMR (400 MHz, $CDCl_3$) of compound (**3q**)

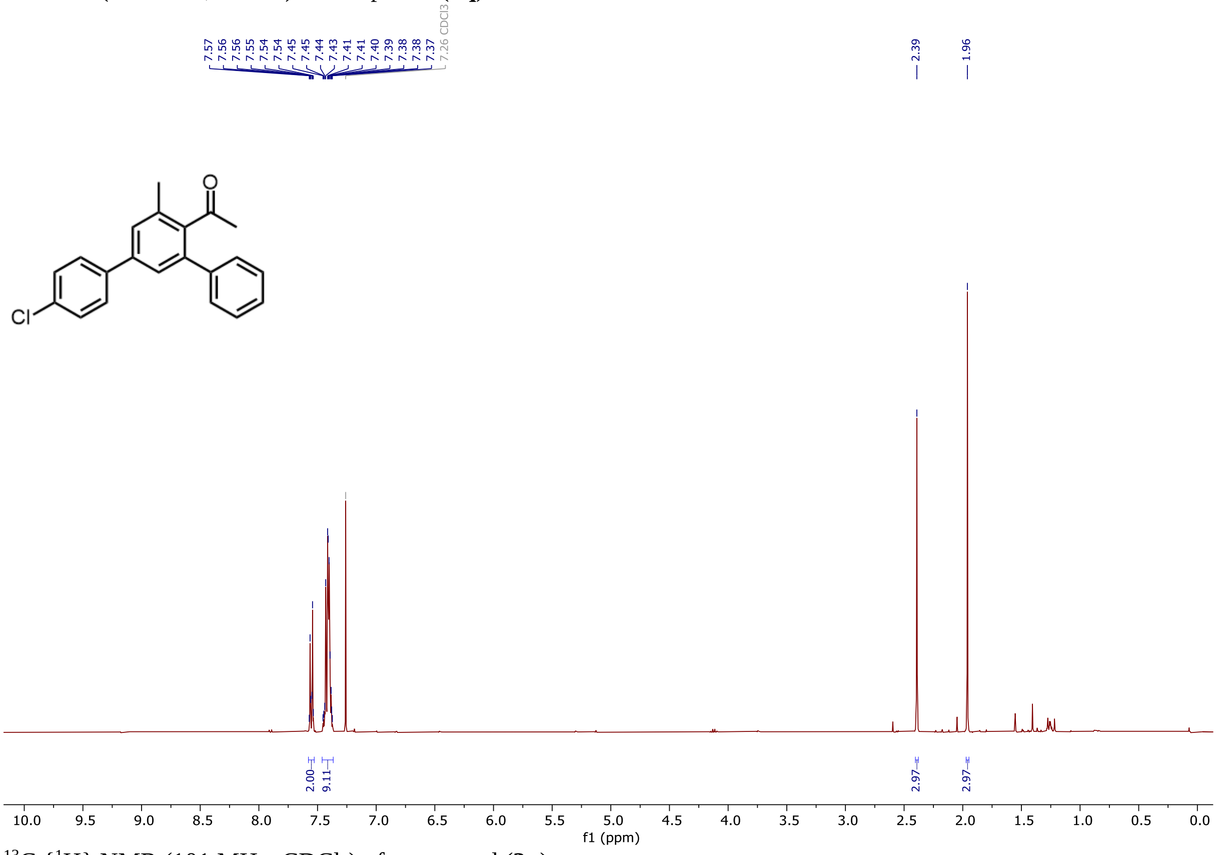

^{13}C {^{1}H} NMR (101 MHz, $CDCl_3$) of compound (**3q**)

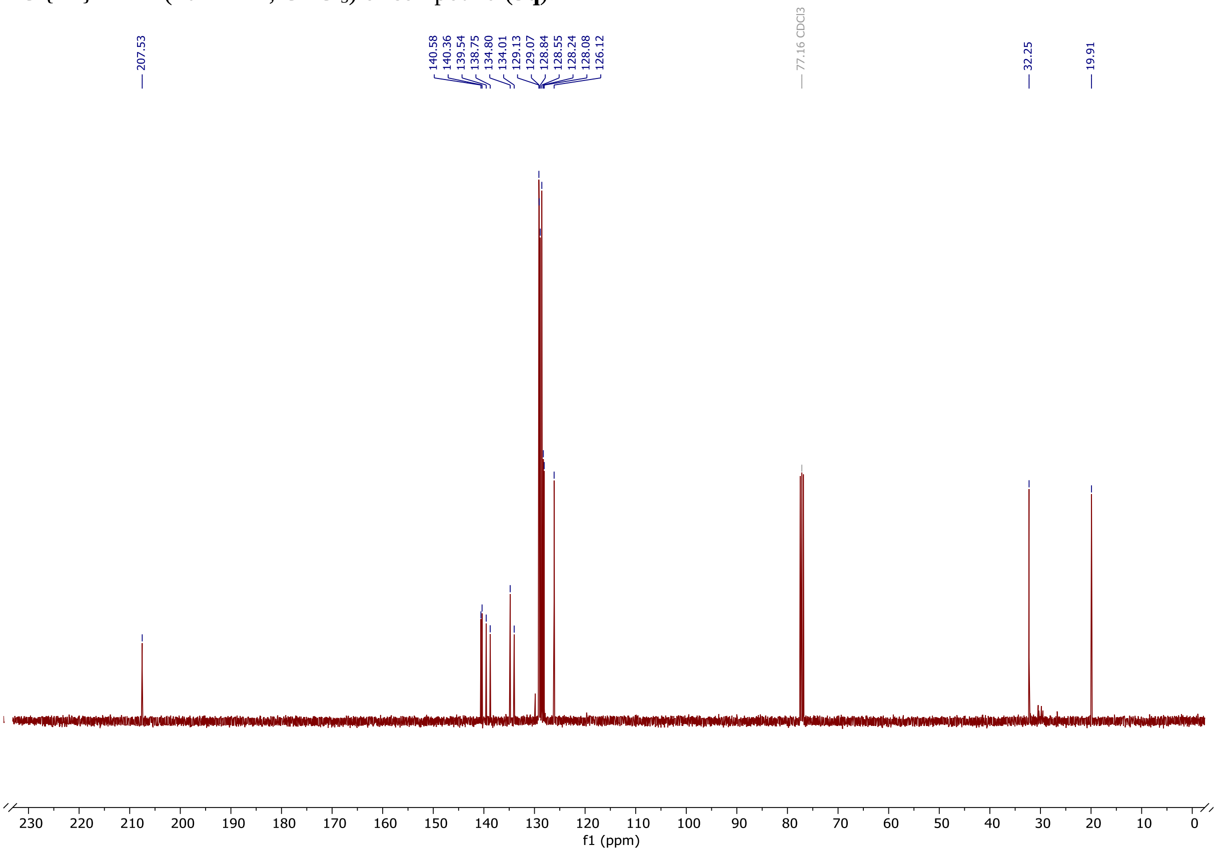

^{1}H NMR (400 MHz, $CDCl_3$) of compound (**3r**)

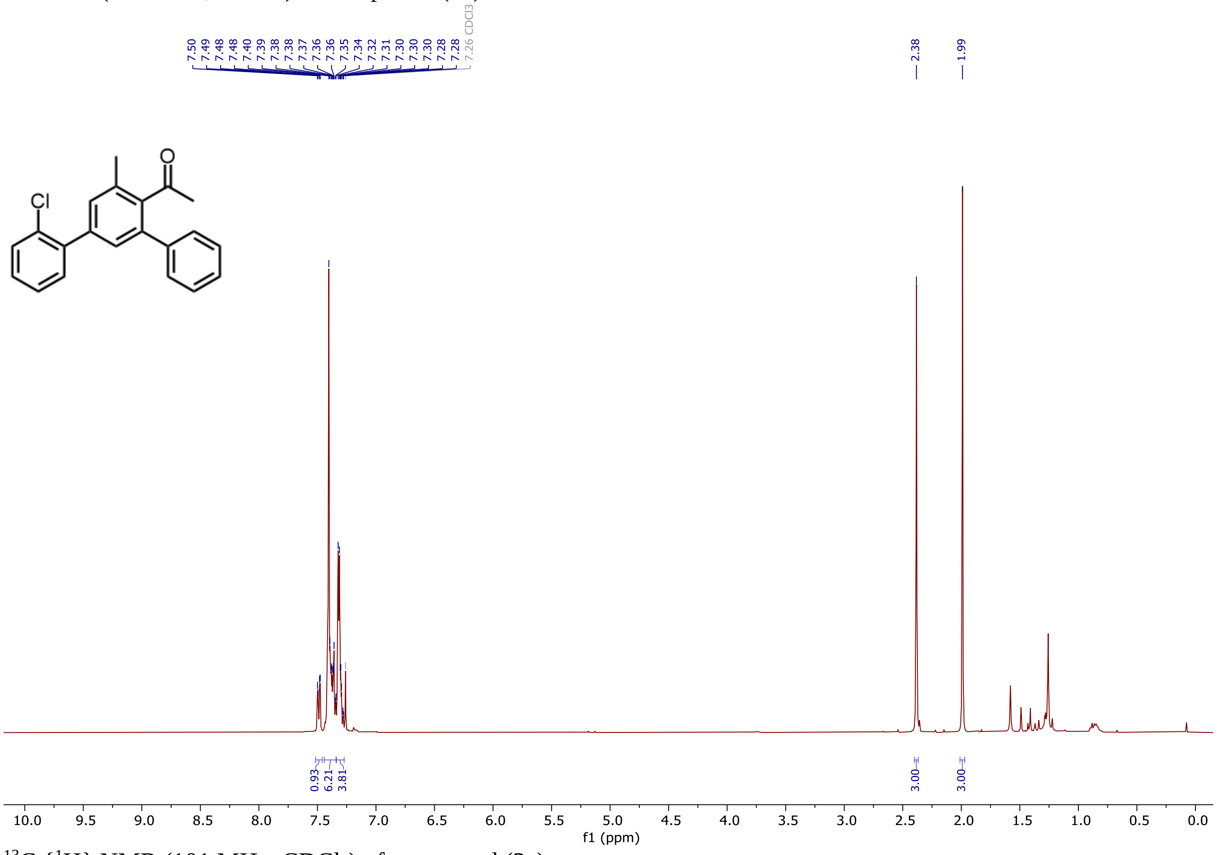

^{13}C {^{1}H} NMR (101 MHz, $CDCl_3$) of compound (**3r**)

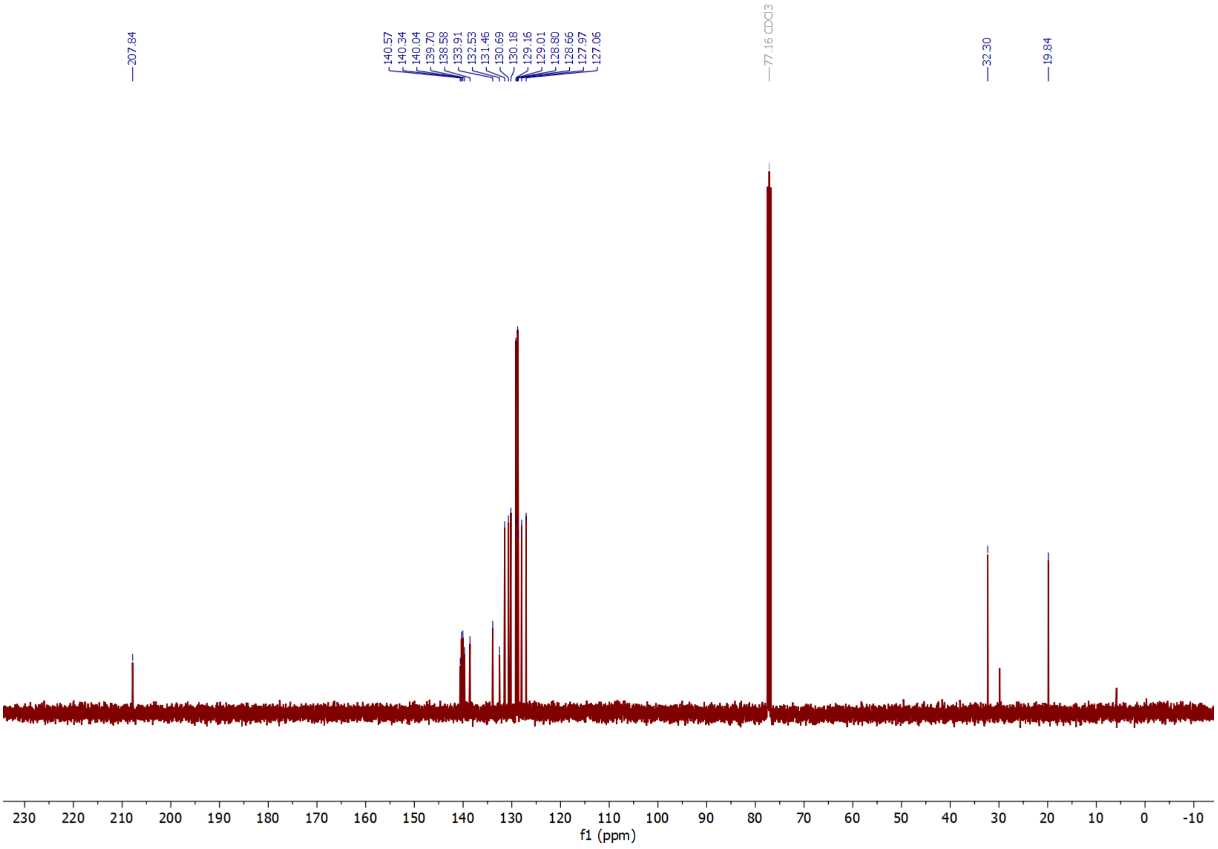

^{1}H NMR (400 MHz, $CDCl_3$) of compound (**3s**)

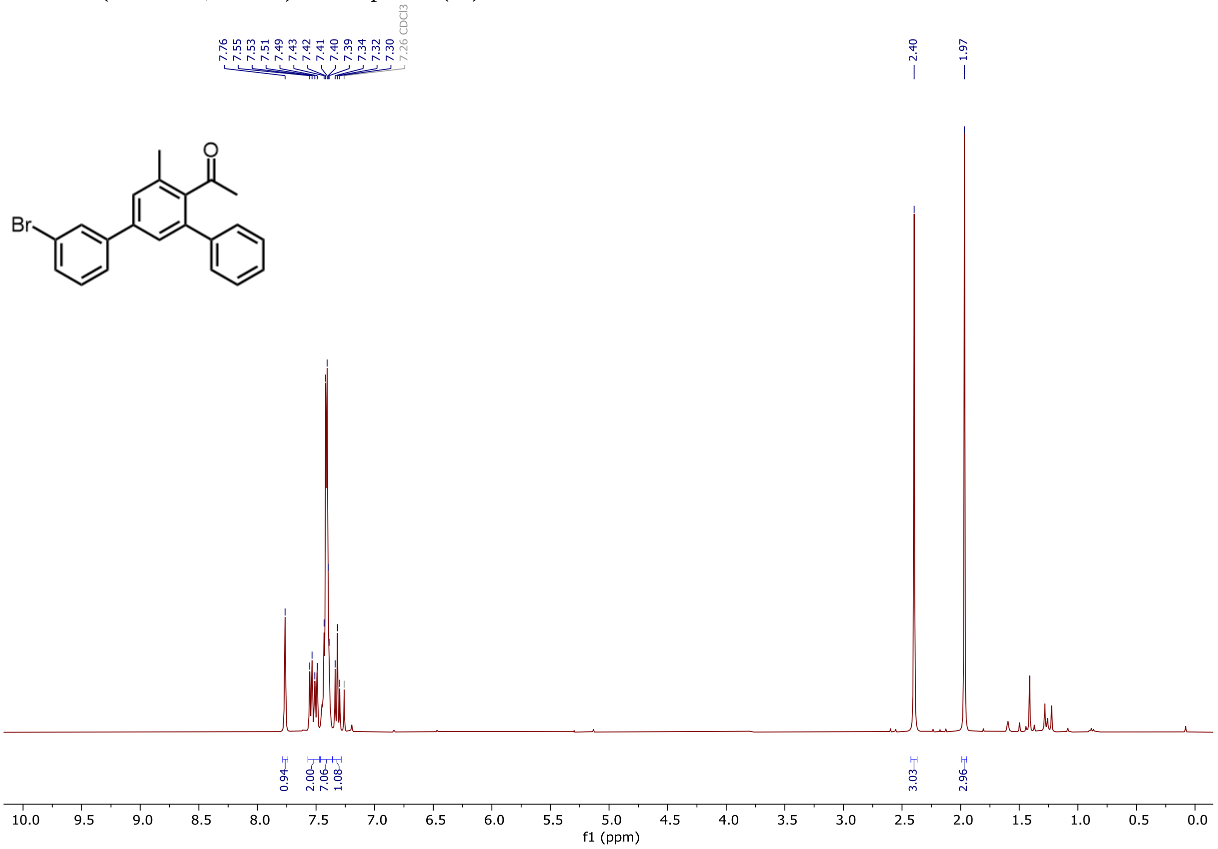

^{13}C {^{1}H} NMR (101 MHz, $CDCl_3$) of compound (**3s**)

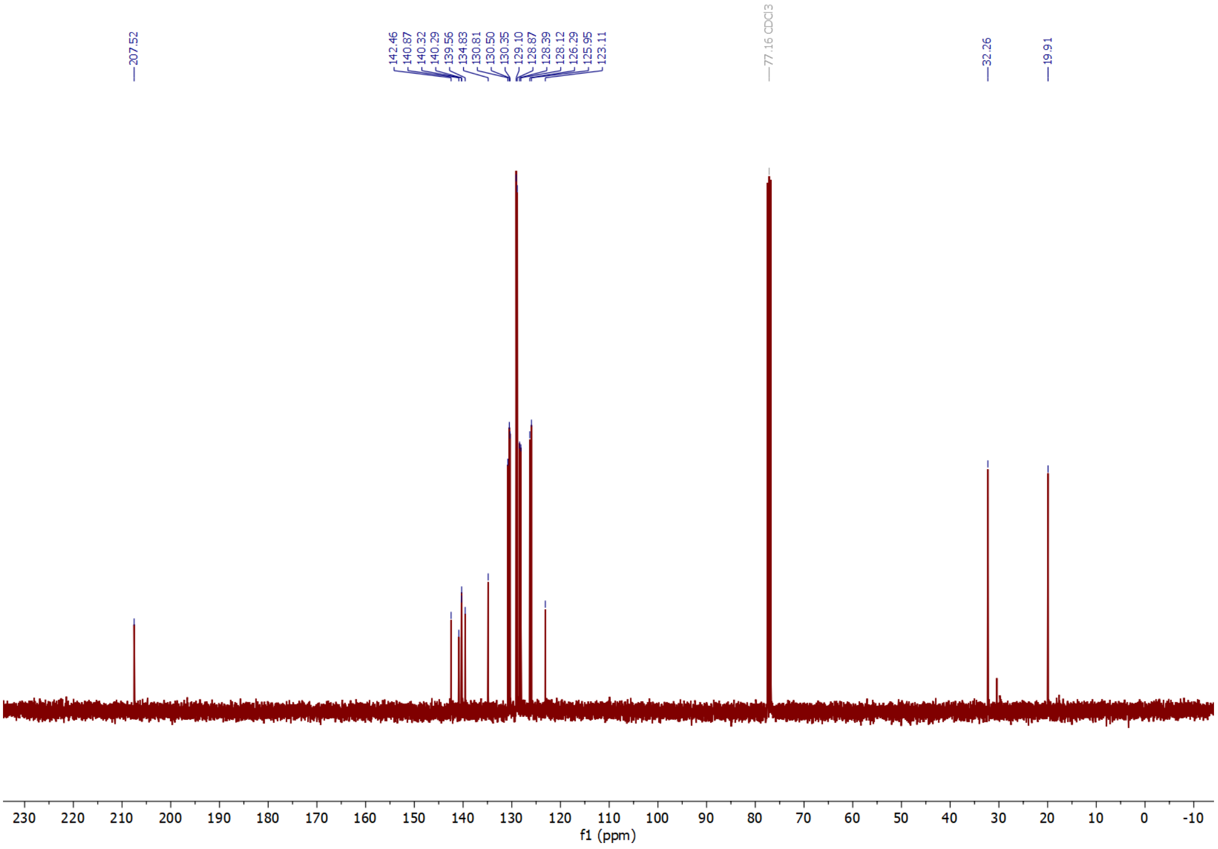

^{1}H NMR (400 MHz, $CDCl_3$) of compound (**3t**)

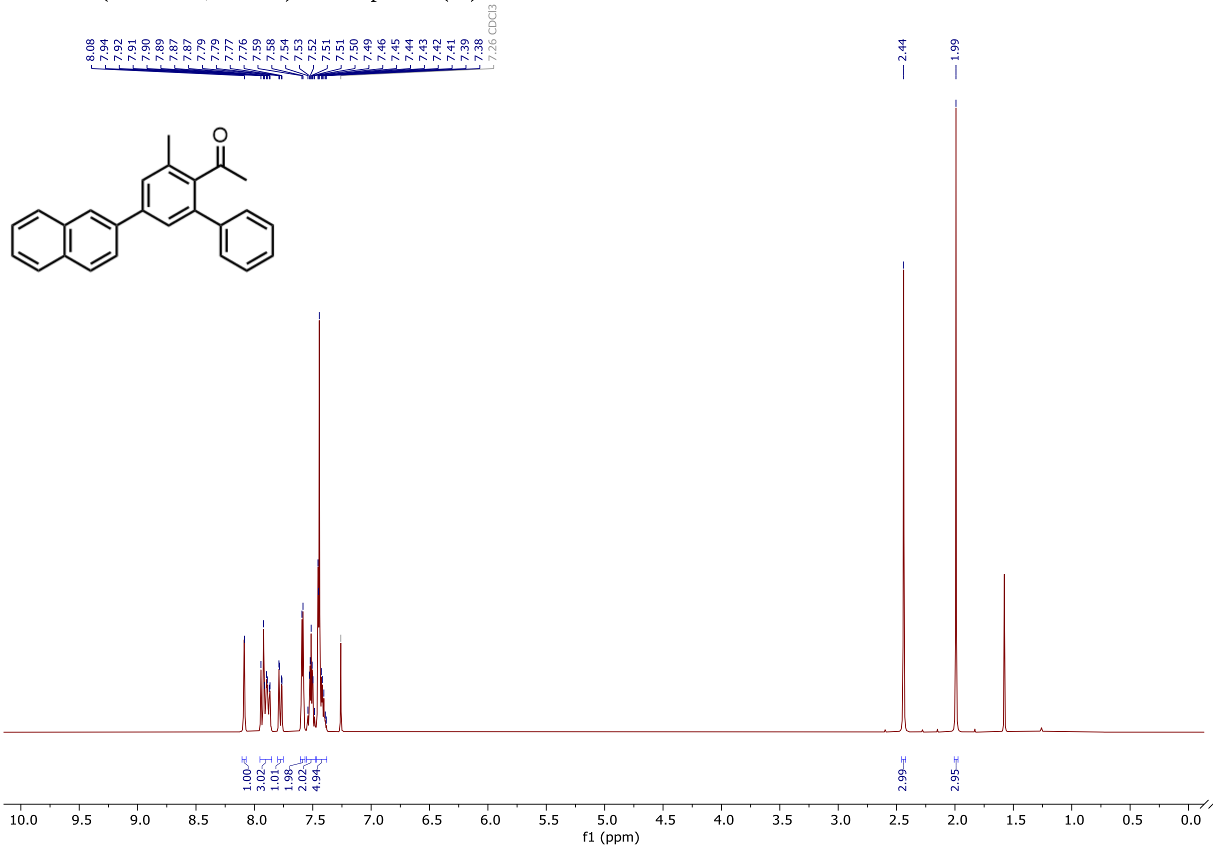

^{13}C {^{1}H} NMR (101 MHz, $CDCl_3$) of compound (**3t**)

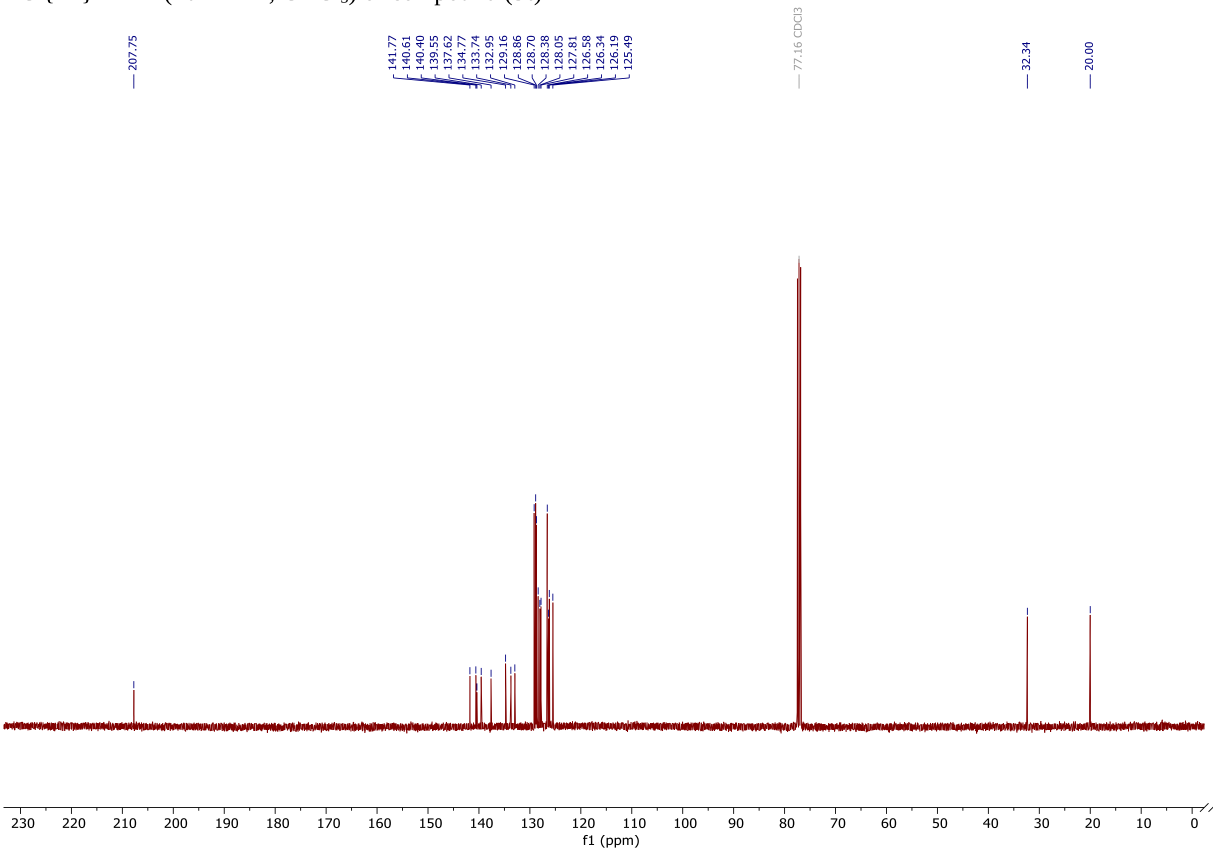

^{1}H NMR (400 MHz, $CDCl_3$) of compound (**3u**)

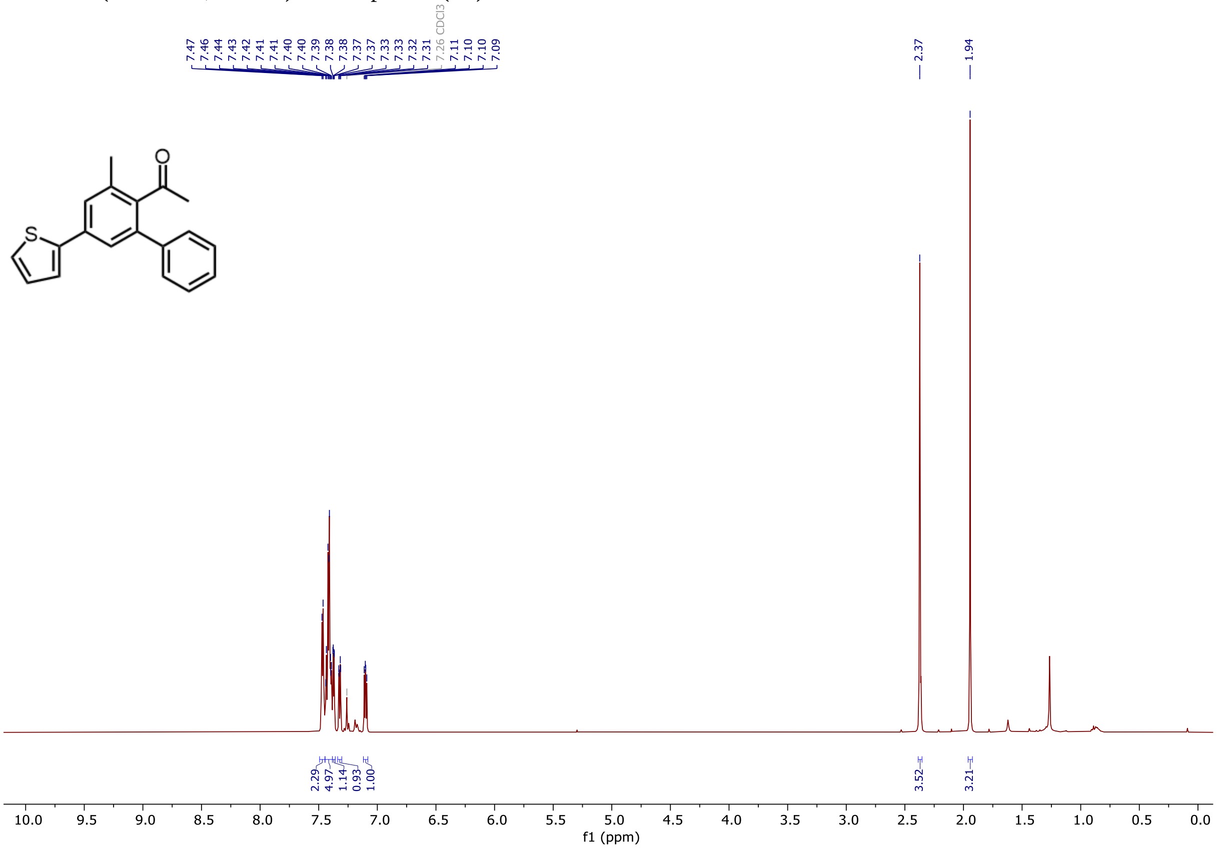

^{13}C {^{1}H} NMR (101 MHz, $CDCl_3$) of compound (**3u**)

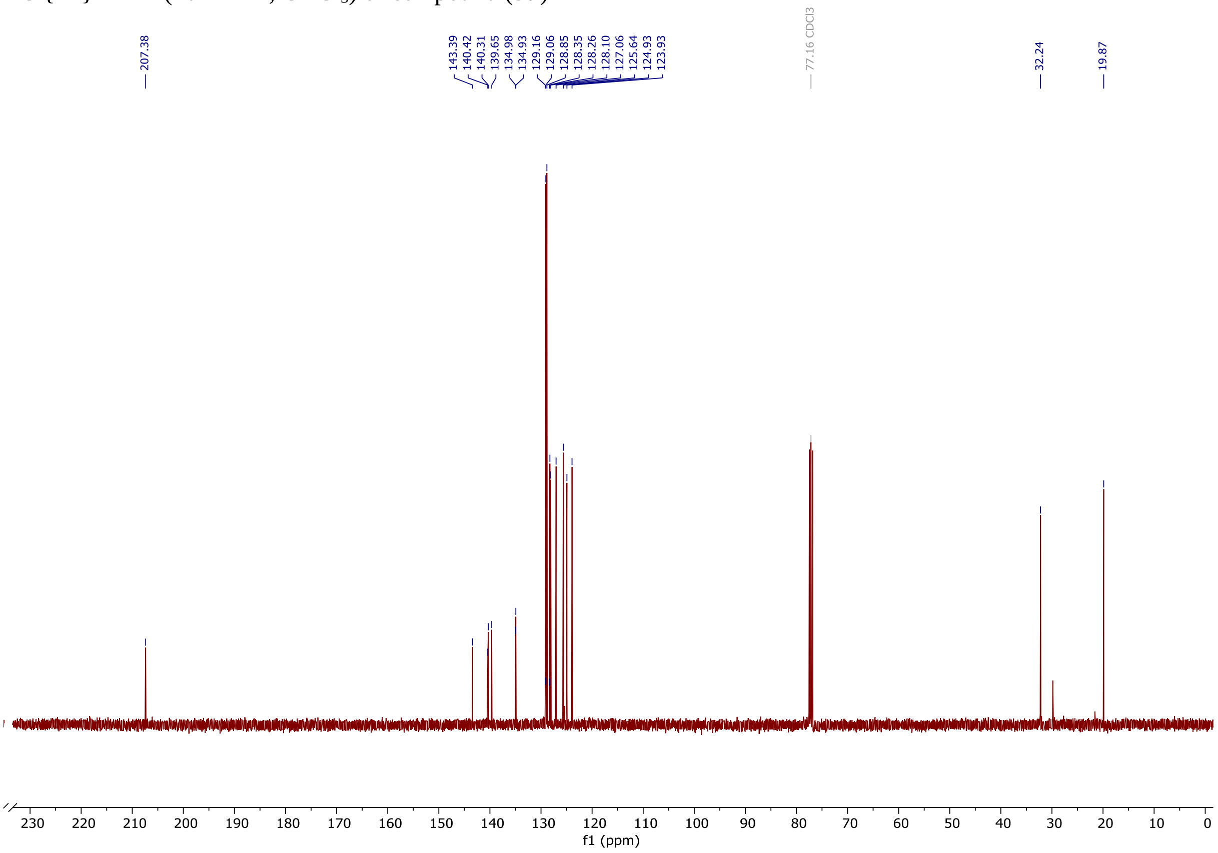

^{1}H NMR (400 MHz, $CDCl_3$) of compound (**3v**)

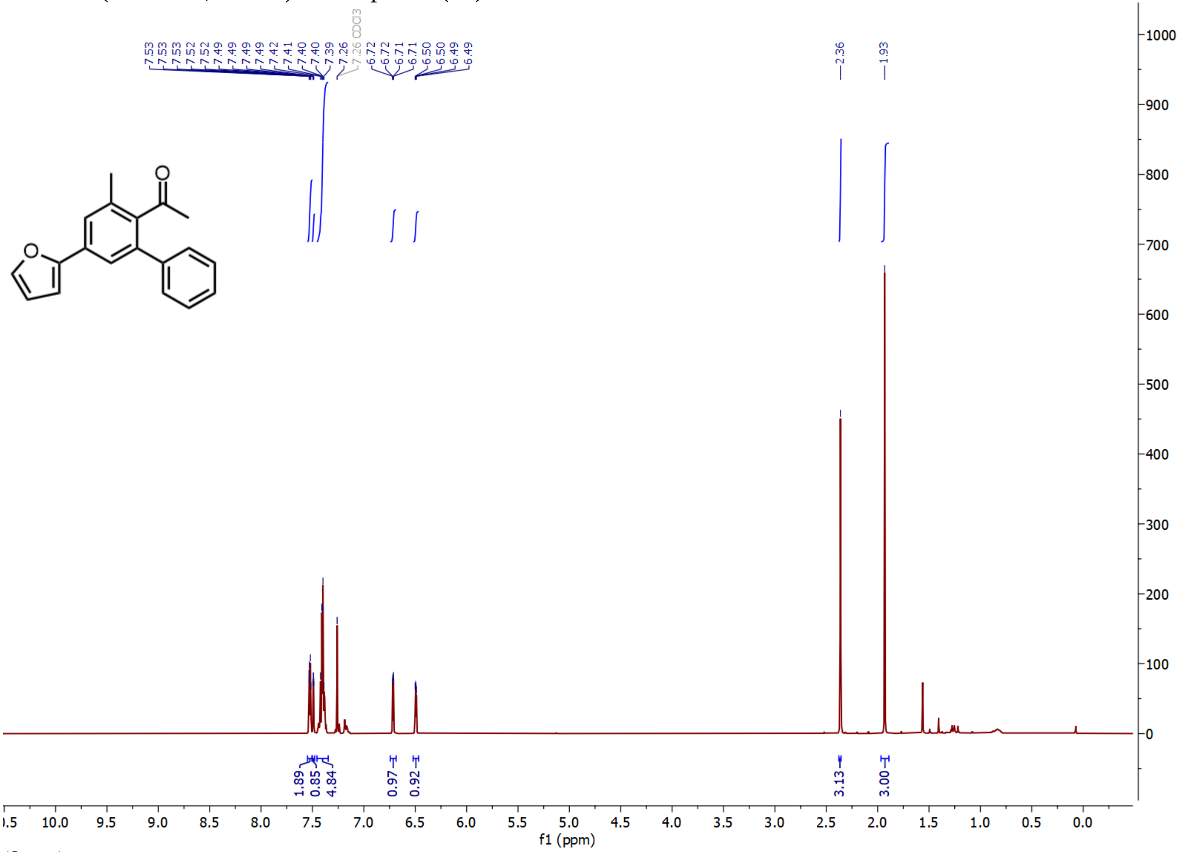

^{13}C {^{1}H} NMR (101 MHz, $CDCl_3$) of compound (**3v**)

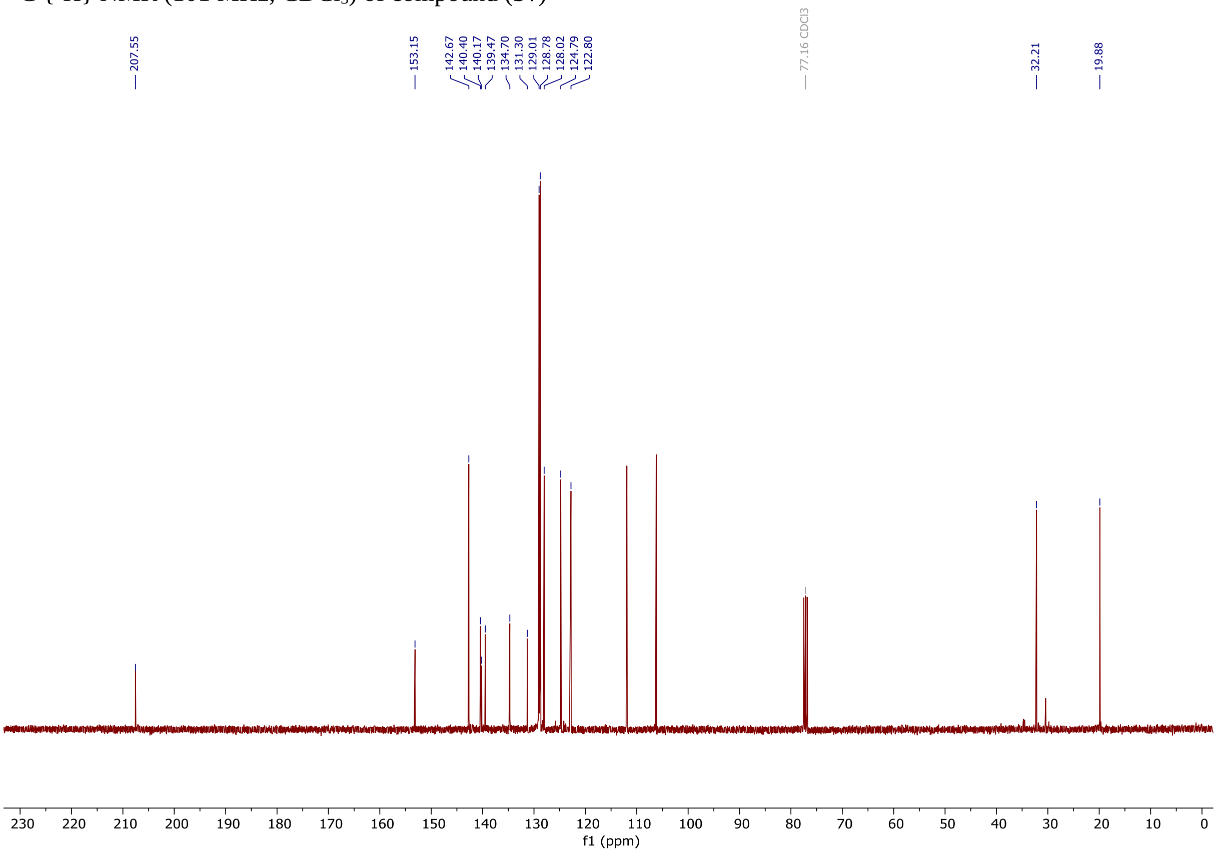

^{1}H NMR (400 MHz, $CDCl_3$) of compound (**3w**)

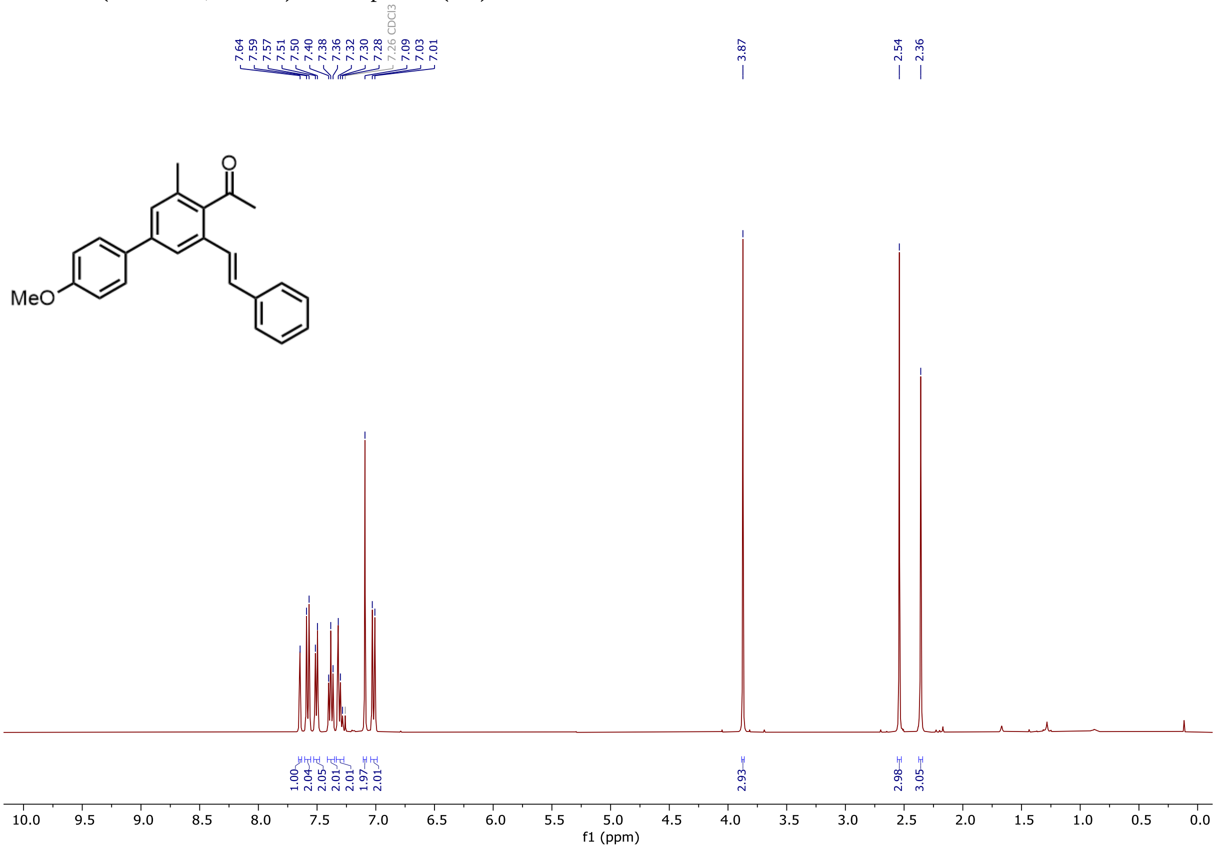

^{13}C {^{1}H} NMR (101 MHz, $CDCl_3$) of compound (**3w**)

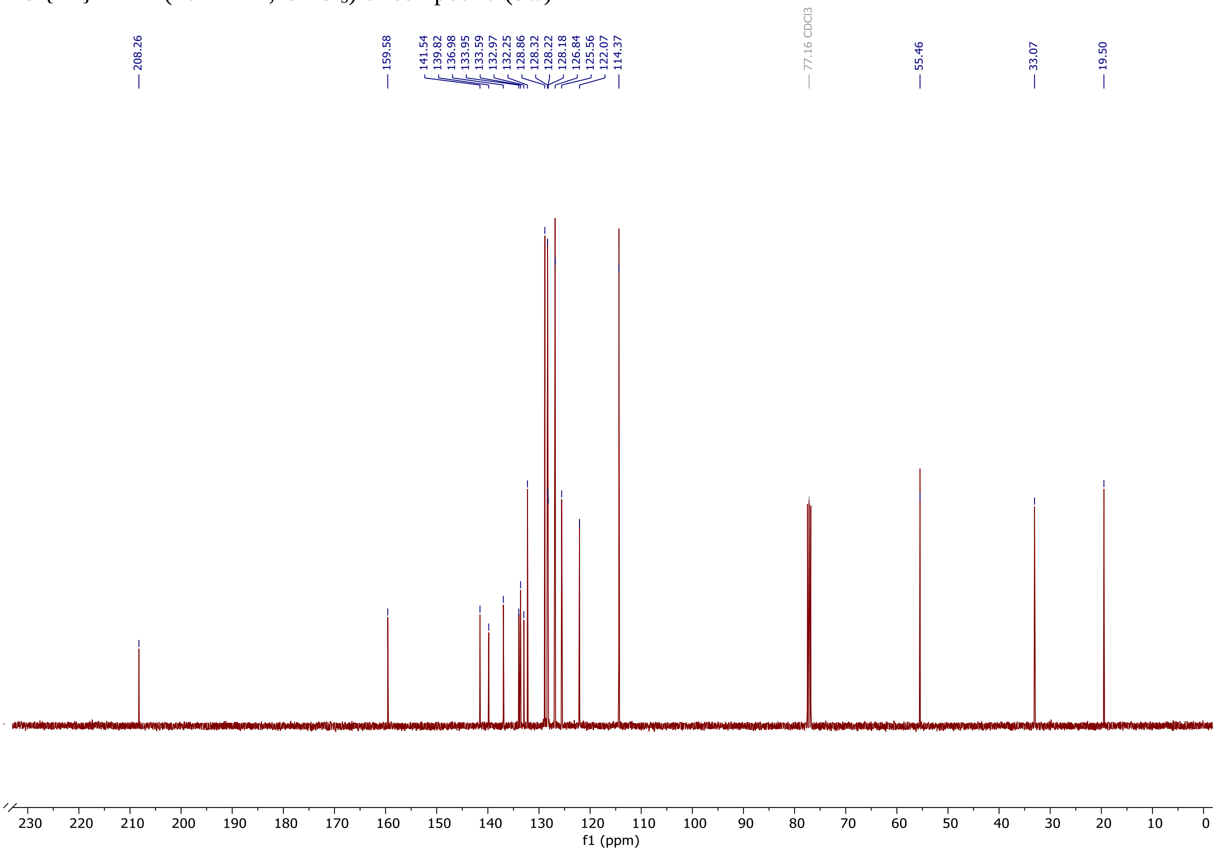

^{1}H NMR (400 MHz, $CDCl_3$) of compound (**3x**)

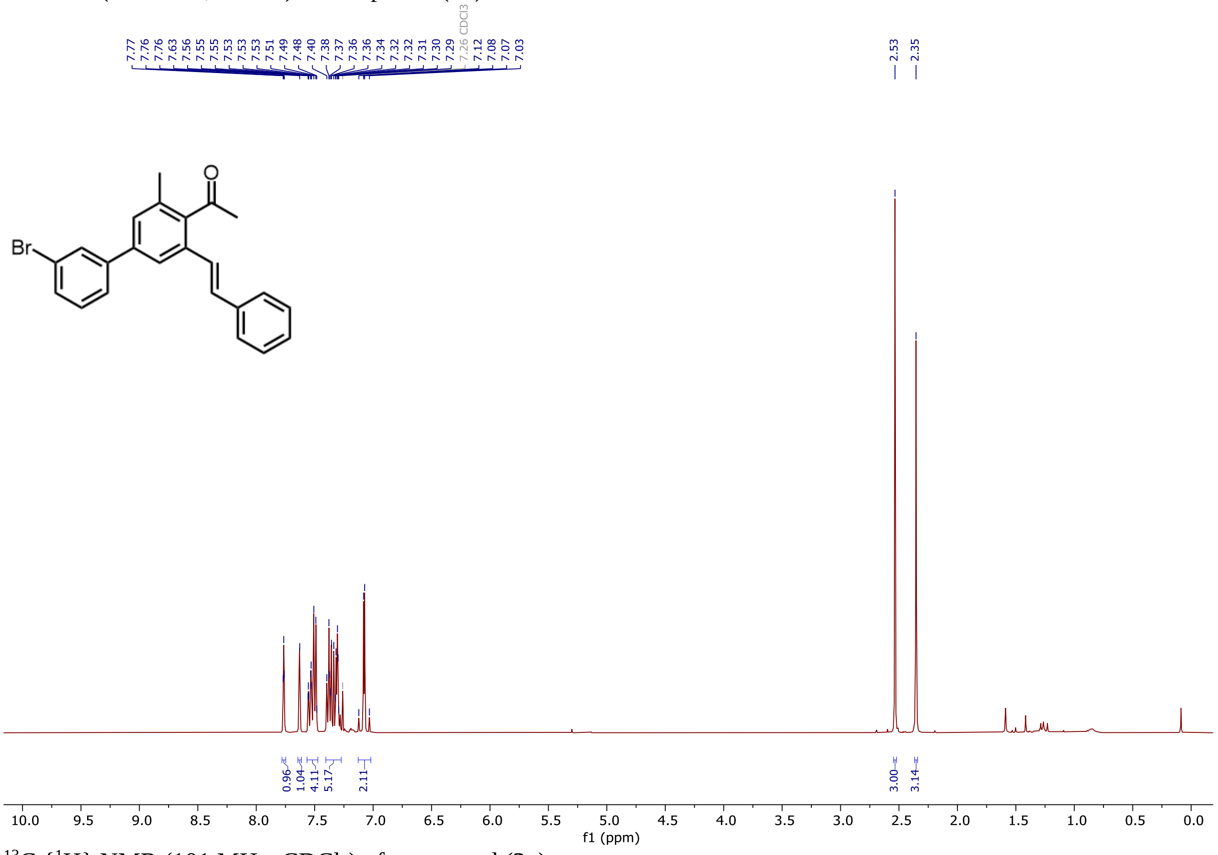

^{13}C {^{1}H} NMR (101 MHz, $CDCl_3$) of compound (**3x**)

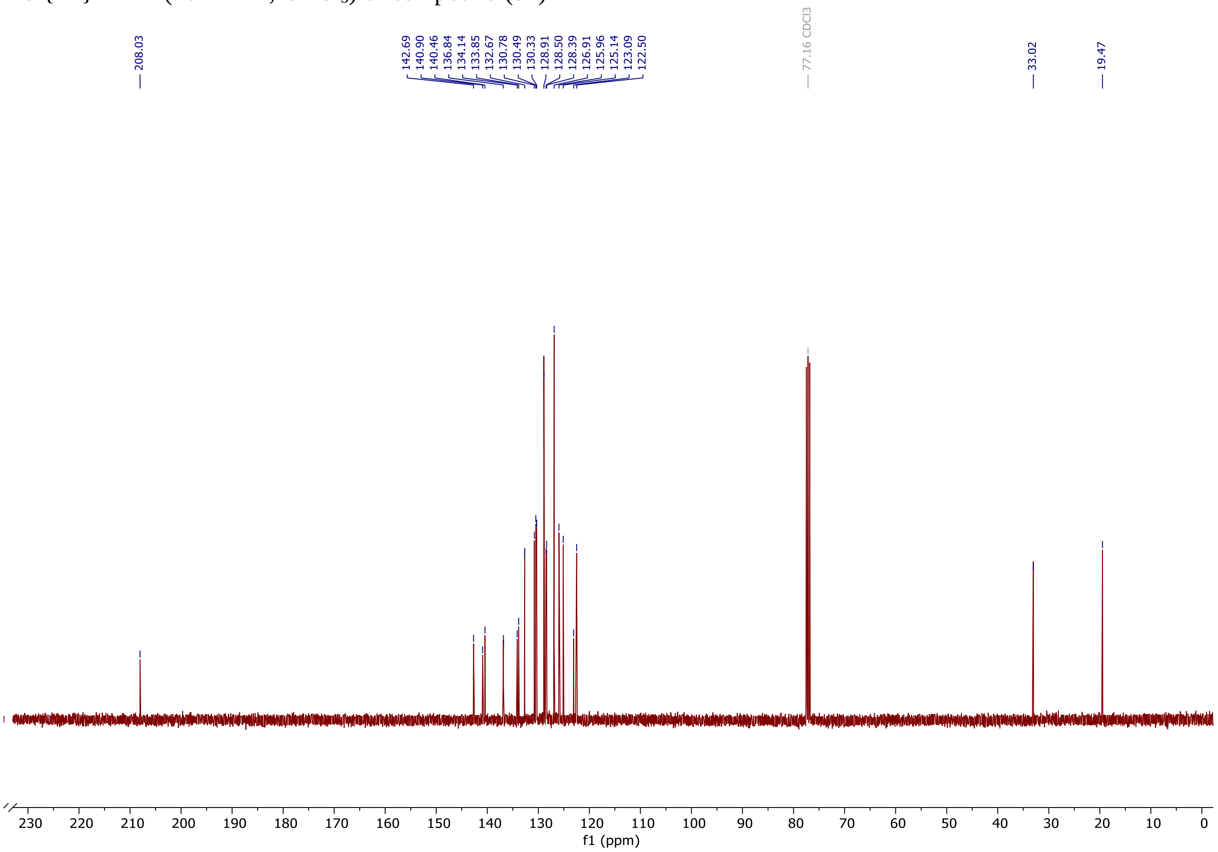

^{1}H NMR (400 MHz, $CDCl_3$) of compound (**3y**)

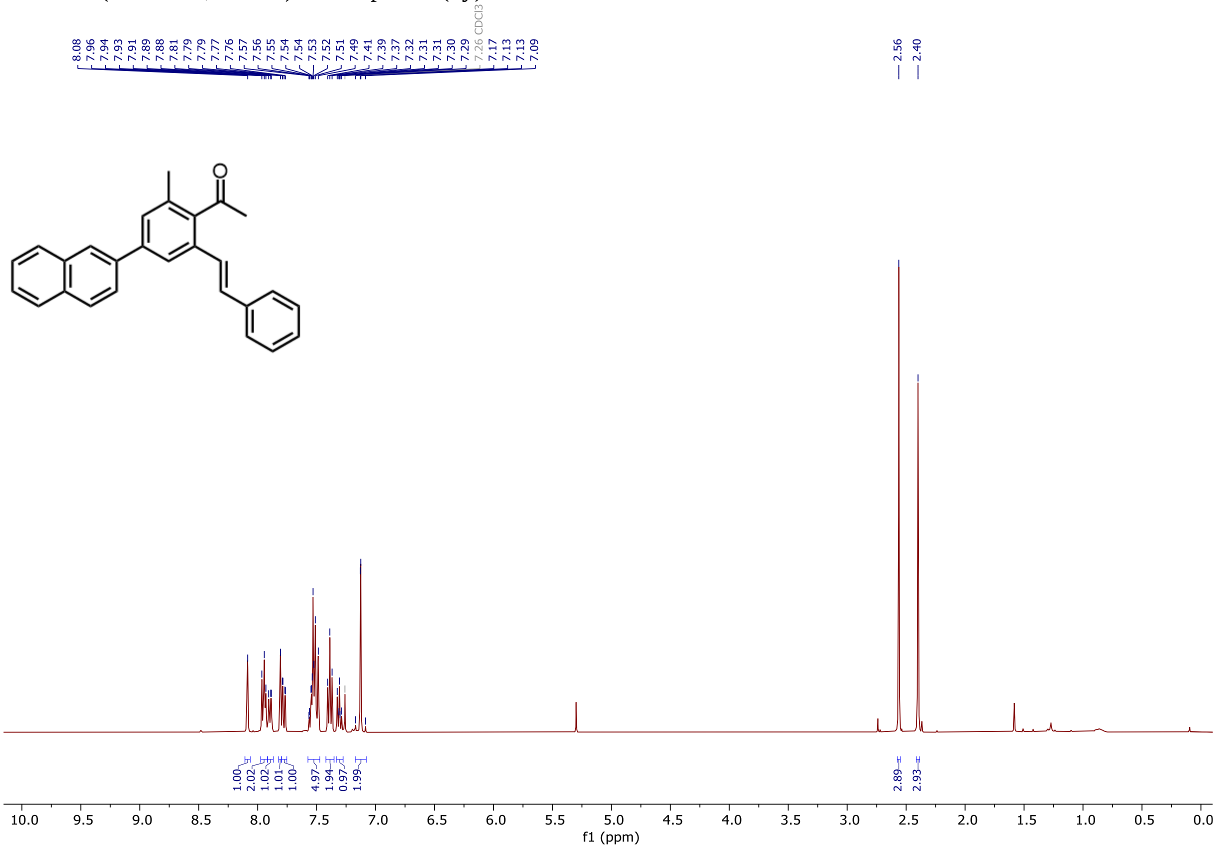

^{13}C {^{1}H} NMR (101 MHz, $CDCl_3$) of compound (**3y**)

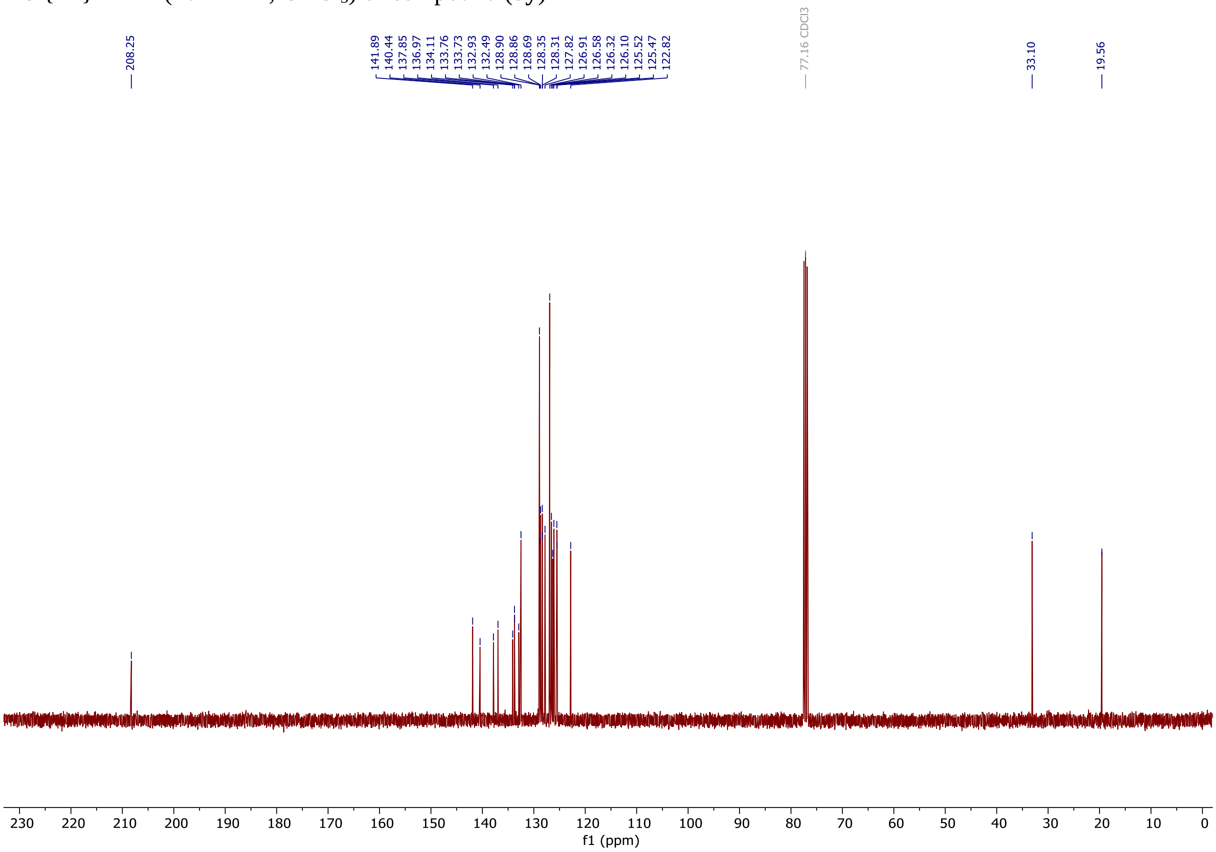

^{1}H NMR (400 MHz, $CDCl_3$) of compound (**3z**)

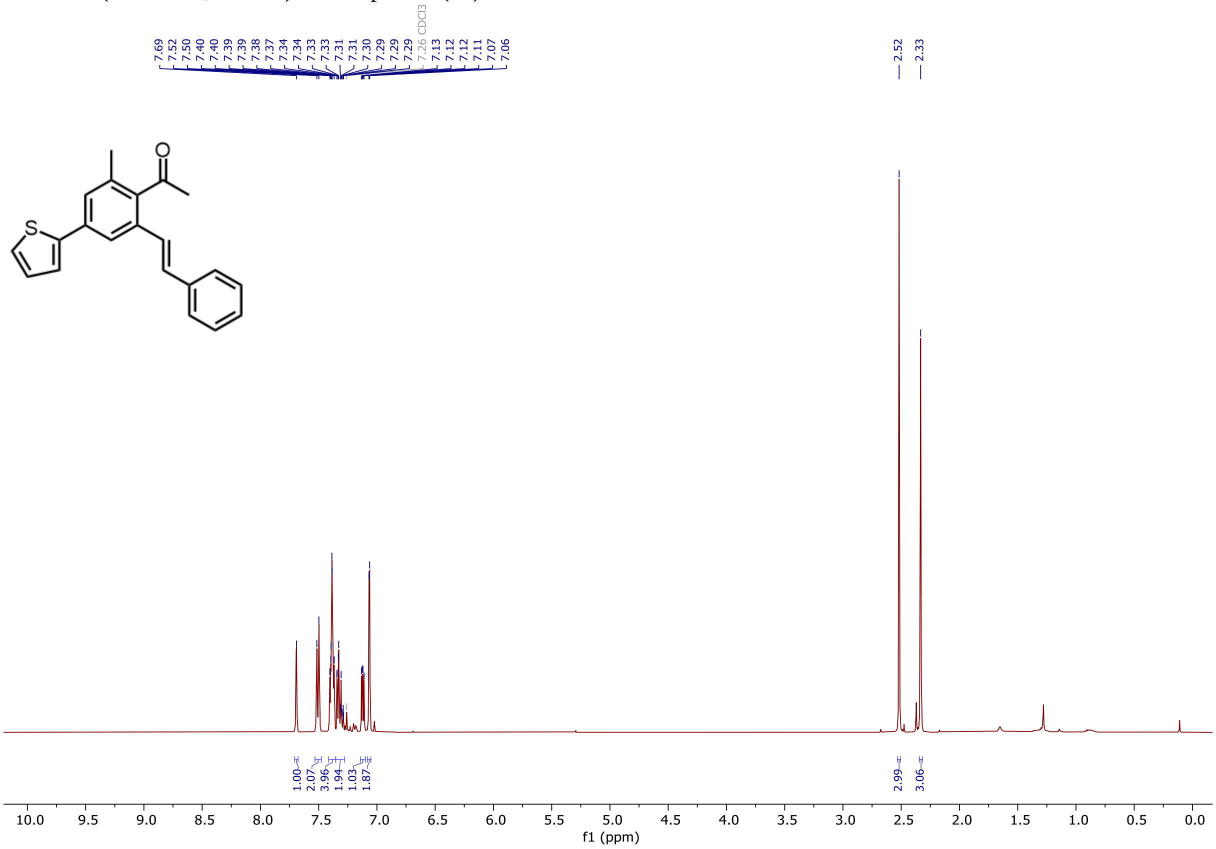

^{13}C {^{1}H} NMR (101 MHz, $CDCl_3$) of compound (**3z**)

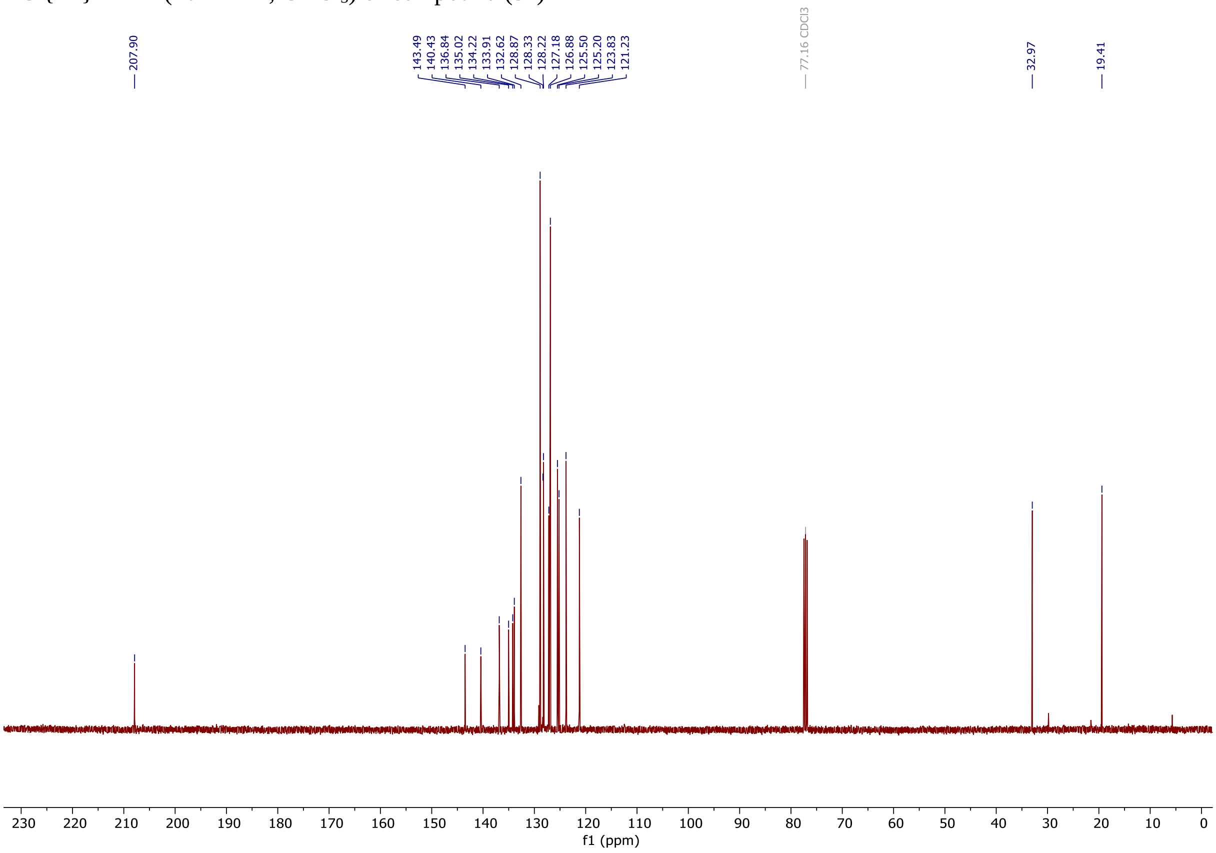

^{1}H NMR (400 MHz, $CDCl_3$) of compound (**7**)

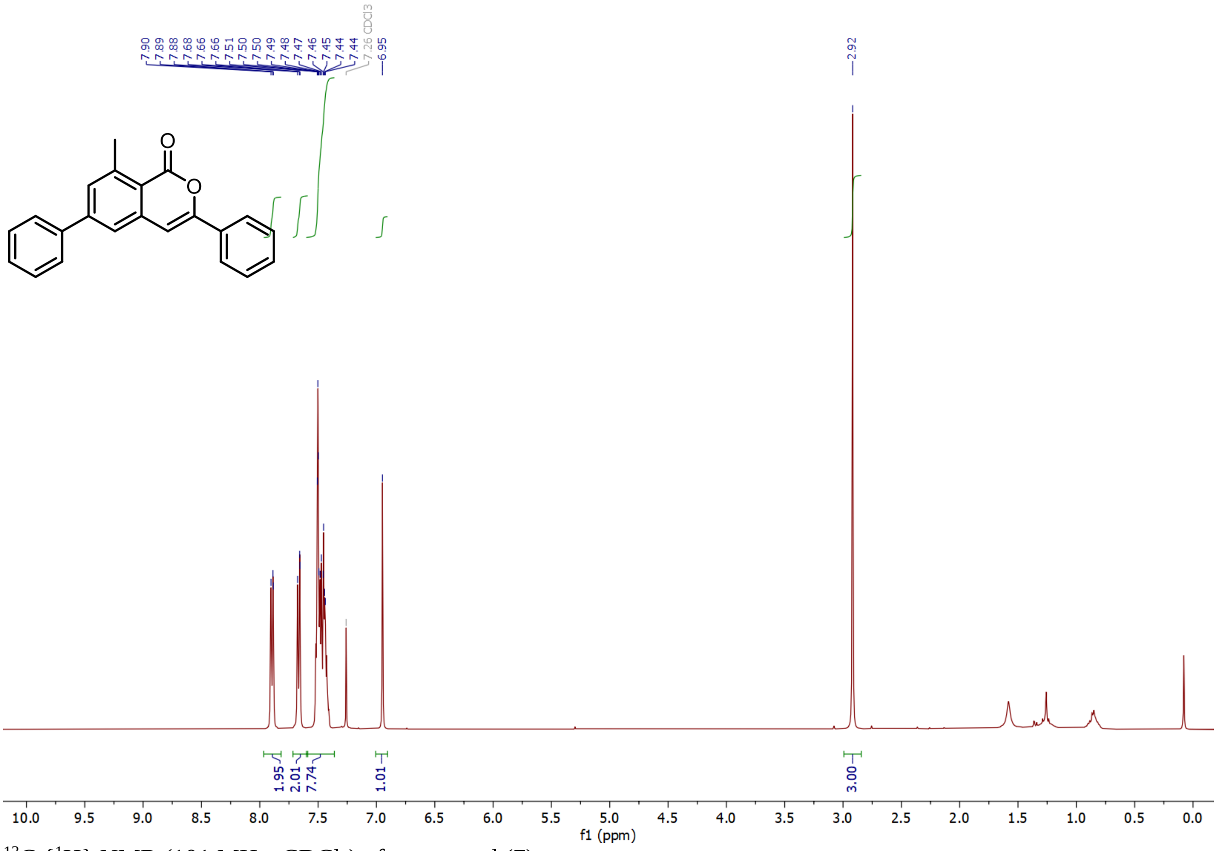

^{13}C {^{1}H} NMR (101 MHz, $CDCl_3$) of compound (**7**)

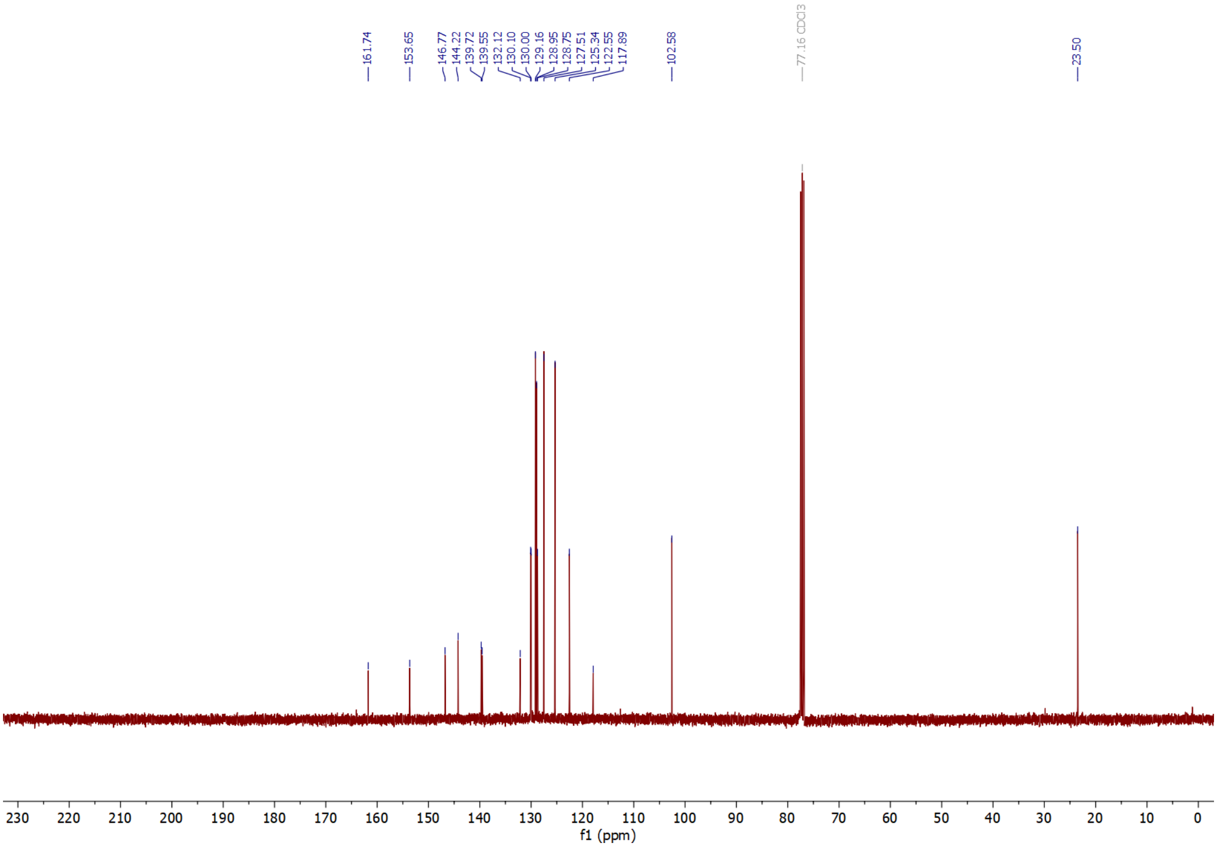

^{1}H NMR (400 MHz, $CDCl_3$) of compound (**8**)

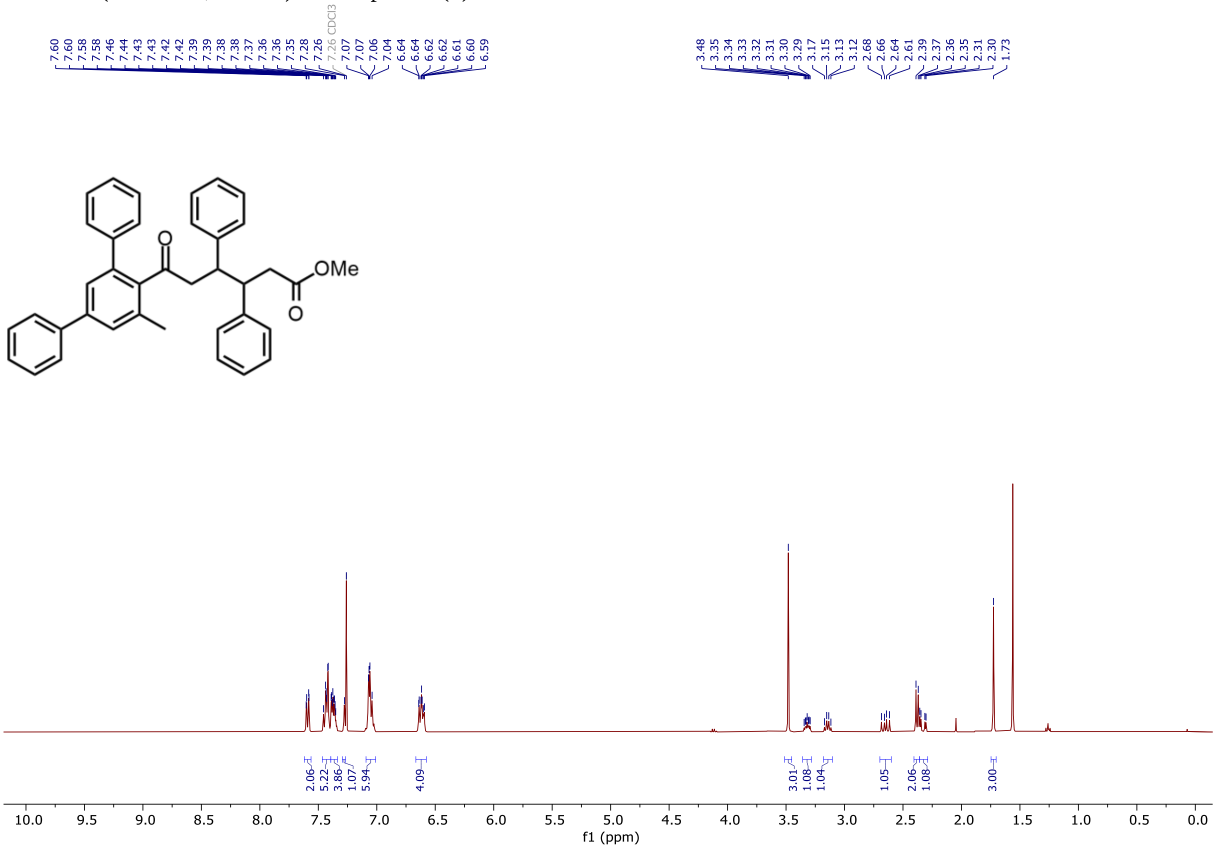

^{13}C {^{1}H} NMR (101 MHz, $CDCl_3$) of compound (**8**)

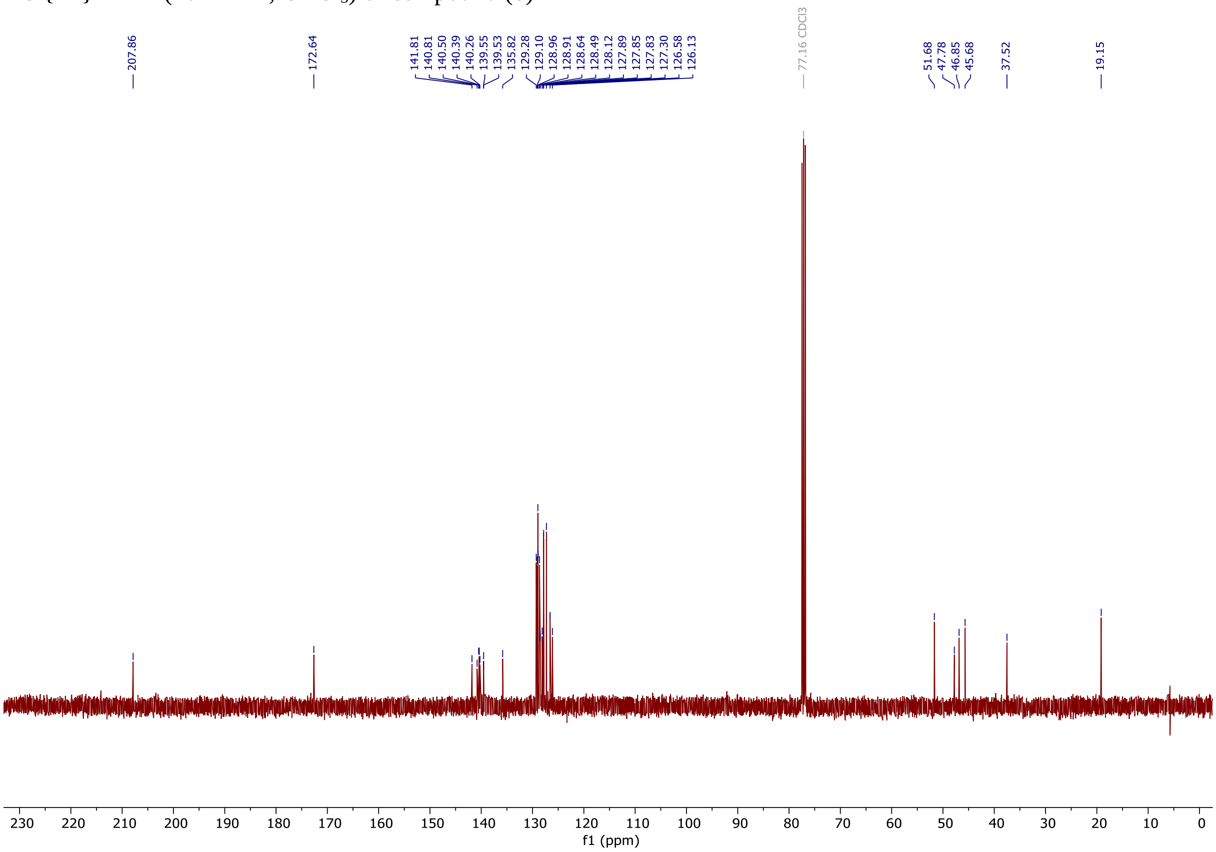

Supplement: Supplementary file 1 — ol2c03879_si_001.pdf [file ol2c03879_si_001.pdf]
